# Genome-wide analysis of Cushion willow provides insights into alpine plant divergence in a biodiversity hotspot

**DOI:** 10.1038/s41467-019-13128-y

**Published:** 2019-11-19

**Authors:** Jia-hui Chen, Yuan Huang, Benjamin Brachi, Quan-zheng Yun, Wei Zhang, Wei Lu, Hong-na Li, Wen-qing Li, Xu-dong Sun, Guang-yan Wang, Jun He, Zhuo Zhou, Kai-yun Chen, Yun-heng Ji, Ming-ming Shi, Wen-guang Sun, Yong-ping Yang, Ren-gang Zhang, Richard J. Abbott, Hang Sun

**Affiliations:** 1 CAS Key Laboratory for Plant Diversity and Biogeography of East Asia, Kunming Institute of Botany, Chinese Academy of Sciences, 650201 Kunming, Yunnan P. R. China; 2 School of Life Sciences, Yunnan Normal University, 650092 Kunming, Yunnan P. R. China; 3 BIOGECO, INRA, Université de Bordeaux, Cestas, France; 4 Beijing Ori-Gene Science and Technology Co., Ltd, 102206 Beijing, P.R. China; 5 State Key Laboratory of Protein and Plant Gene Research, Peking-Tsinghua Center for Life Sciences, and School of Life Sciences, Peking University, 100871 Beijing, P.R. China; 6 School of Life Sciences, Peking University, 100871 Beijing, P.R. China; 7 Institute of Tibetan Plateau Research at Kunming, Kunming Institute of Botany, Chinese Academy of Sciences, 650201 Kunming, Yunnan P. R. China; 8 The Germplasm Bank of Wild Species, Kunming Institute of Botany, Chinese Academy of Sciences, 650201 Kunming, Yunnan P. R. China; 9 School of Biology, University of St. Andrews, St. Andrews, Fife KY16 9TH UK

**Keywords:** Biogeography, Phylogenomics, Genetic variation, Plant evolution

## Abstract

The Hengduan Mountains (HDM) biodiversity hotspot exhibits exceptional alpine plant diversity. Here, we investigate factors driving intraspecific divergence within a HDM alpine species *Salix brachista* (Cushion willow), a common component of subnival assemblages. We produce a high**-**quality genome assembly for this species and characterize its genetic diversity, population structure and pattern of evolution by resequencing individuals collected across its distribution. We detect population divergence that has been shaped by a landscape of isolated sky island-like habitats displaying strong environmental heterogeneity across elevational gradients, combined with population size fluctuations that have occurred since approximately the late Miocene. These factors are likely important drivers of intraspecific divergence within Cushion willow and possibly other alpine plants with a similar distribution. Since intraspecific divergence is often the first step toward speciation, the same factors can be important contributors to the high alpine species diversity in the HDM.

## Abstract (web summary)

Exceptional alpine plant diversity exists in the Hengduan Mountains. Here, through genome assembly and population genomics studies, the authors find notable intraspecific divergence among Cushion willow populations isolated by the sky island-like habitats and consider it contributes to speciation and biodiversity.

## Introduction

**Fig. 1:**
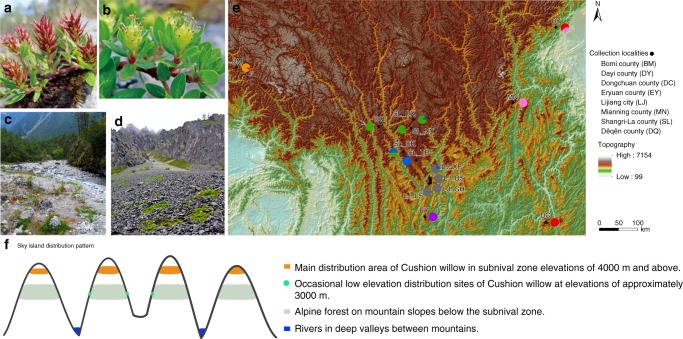
Cushion willow distribution pattern and locations of population samples. **a** Female individual with flowers. **b** Male individual with flowers. **c** Low elevation Cushion willow population located on open river bank at an elevation of 2950 m above sea level. **d** High elevation Cushion willow population on a scree slope at an elevation of 4000 m. **e** Sample locations of the 14 Cushion willow populations in HDM and nearby area of the eastern Himalaya. Pie charts at sampling locations indicate the distribution of genetic groups identified by a Structure analysis of the population. **f** Scheme of sky island distribution pattern

The Hengduan Mountains (HDM) biodiversity hotspot is part of the Tibeto-Himalayan region (THR), which also comprises the Qinghai-Tibetan Plateau (QTP) and the Himalayas^1,2^. Within the HDM a series of parallel, mostly north-south oriented high mountains separated by deep valleys occurs, with elevations ranging from 1000 m on valley floors to >6000 m on mountain peaks (Fig. 1). The region possesses exceptional species richness (~12,800 seed plant species are recognized^3^), with many different alpine plants present in the subnival zone^2,4^. This has motivated recent interest in understanding the origin and evolution of biodiversity in the QTP and within the HDM in particular^2,5^.

One hypothesis for the rich biodiversity found in mountainous regions like the HDM is uplift-driven diversification^6^; that is, orogeny produced topographical features creating diverse habitats and isolated populations, facilitating rapid intraspecific divergence leading to in situ speciation. In addition, the connectivity of populations could have been influenced by Quaternary climate fluctuations, contributing also to population divergence and speciation^1,7,8^. However, the relatively few studies aimed at testing these hypotheses have employed a limited number of molecular markers^2,4,5,7,8^. Here we report a population genomics analysis to examine the genetic structure, demographic history and genetic basis of adaptation to subnival conditions of a typical subnival species of *Salix* (willow) that occurs in the HDM. Our aim was to determine which factors shaped the genetic structure of this species, providing insights into the factors that could have also potentially driven intraspecific divergence within other alpine species in the HDM, and thus contributed to the high alpine plant diversity within this region.

The dioecious genus *Salix* (Salicaceae) exhibits high species richness in the HDM, with approximately 152 species recognized, accounting for 57% and 27% of total *Salix* species distributed in China and worldwide, respectively^9^. Members of the genus are important components of alpine and temperate vegetation. *Salix brachista* Schneid is a typical alpine willow mainly distributed in the subnival zones of mountains in the HDM, and also occasionally in neighboring areas (i.e., the eastern Himalaya and middle Yunnan Plateau), where it is a dominant component of many subnival assemblages. Morphologically, it is a cushion plant (hereafter referred to as Cushion willow) with a creeping stem and lateral branches growing to a height of usually no more than 5 cm (Fig. 1a–d). It is recognized as a sky island^10^ plant in the HDM, with populations distributed at elevations of around 4000 m on mountain peaks isolated by forests and low-lying terrain below. Occasionally, it is also found at lower elevations, for example in open habitats on river banks at ~3000 m, amidst high altitude forest (Fig. 1c, e, f).

Here, we report a high**-**quality genome sequence of Cushion willow, which is used as a reference for a population genomics study of 77 individuals collected from 14 populations across the species’ distribution. We then characterize the species’ genetic diversity, population structure, demographic history, and footprints of adaptive evolution to the alpine environment. We interpret the results in the context of explaining rapid intraspecific divergence leading to high plant species richness within the HDM.

## Results

### Genome assembly

We sequenced the genome of a female Cushion willow plant with a nuclear genome size estimated to be ~421 Mb and ~400 Mb by flow cytometry (Supplementary Table 1) and *K*-mer analysis (Supplementary Fig. 1), respectively. This plant was originally collected from the Tianbao Mountain in Shangri-La County, Yunnan, China, and subsequently maintained within the Kunming Botanical Garden. Cytogenetic studies detected 38 chromosomes, indicating that the sequenced individual is diploid (2*n* = 2*x* = 38) (Supplementary Fig. 2). We generated 17.5 Gb (~119 million reads) of Illumina PCR-free short reads, 50 Gb (~6.8 million reads, average 7.3 kb) of PacBio single-molecule long reads, and 29.69 Gb of Oxford Nanopore Technologies (ONT) long reads (for statistics of ONT reads see Supplementary Table 2), corresponding to approximately 43×, 125×, and 74× coverage of the whole genome, respectively (Supplementary Table 3). We used Canu^11^ pre-corrected ONT reads for genome assembly by SMARTdenovo. This assembly was polished for two and five rounds based on PacBio long reads and Illumina short reads, respectively, to generate a 351.67 Mb genome assembly with 245 contigs (contig N50 = 5.34 Mb) (Supplementary Table 4). The ~50 Mb of unassembled genomic sequence consisted mainly of highly repetitive sequences, possibly from centromeres and telomeres (see Methods for details). In contrast, protein-coding regions were assembled to near completeness as shown by BUSCO evaluation below.

**Fig. 2:**
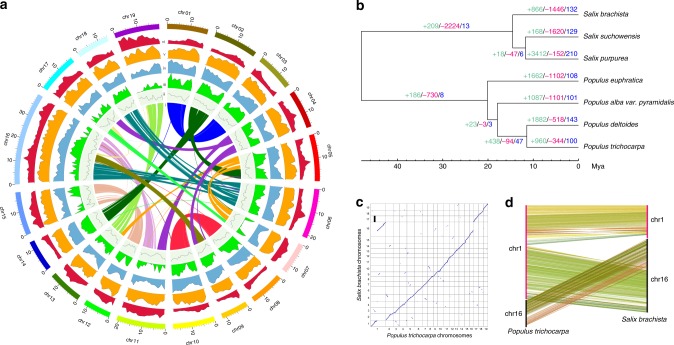
Cushion willow evolution. **a** Circos plot showing the genomic features of *Salix brachista*. The features highlighted from innermost to outermost circle are (i) intra-genome collinear blocks connected by curved lines, (ii) GC content (30%–50%), (iii) SNP density, (iv) gene density (0–50 per window), (v) distribution of Class II TE (scale: 0%–5%), (vi) distribution of Class I TE (scale: 0%–5%). All statistics are computed for windows of 200 kb. **b** Gene family evolution of Cushion willow. The phylogenetic tree was constructed from sequences of 3040 single-copy genes shared by ingroup Sal-SP species. The number of gene families that expanded (turquoise), contracted (magenta) and were rapidly evolving (blue) in each lineage after speciation are indicated above the corresponding branch. **c** Gene collinearity between *S. brachista* and *P. trichocarpa*. The *x*-axis and *y*-axis correspond to the *P. trichocarpa* and *S. brachista* chromosomes, respectively. **d** Collinearity between chromosome 1 and 16 of *S. brachista* and *P. trichocarpa*

Using Hi-C (in vitro fixation of chromosomes) data, 337.28 Mb (99.32%) of the contig sequences were anchored to 19 pseudo-chromosomes (Fig. 2a, Supplementary Fig. 3), which corresponds to the haploid chromosome number of the species. We proceeded to running gap closure and removing redundancy, before further polishing the assembly with two additional runs of Illumina short reads. The final reference assembly was 339.59 Mb in length and included 78 contigs (contig N50 = 9.52 Mb) and 19 pseudo-chromosomes (scaffold N50 = 17.92 Mb). The length of pseudo-chromosomes ranged from 11.69 Mb to 39.69 Mb (Table 1, Supplementary Table 4, 5). The chloroplast and mitochondrial genomes were assembled into circular DNA molecules of 155,604 bp and 608,983 bp, respectively (Supplementary Table 5). We detected a total of 2,419,503 heterozygous sites (comprising 2,133,443 SNPs and 286,060 indels), which corresponds to a heterozygosity rate of approximately 0.71%. About 98.49% of Illumina short reads were mapped successfully back to the genome assembly, and about 98.82% of the assembly was covered by at least 10× reads, indicating that the current assembly covered most unique genomic regions and is highly accurate at the single-nucleotide level (Supplementary Table 6).

**Table 1:** Statistics of the Cushion willow genome assembly

Total assembly size (Mb)	339.588
Total number of contigs	78
Total anchored size (Mb)	337.27
Maximum contig length (Mb)	21.273
Minimum contig length (Kb)	57.08
Contig N50 length (Mb)	9.522
Contig L50 count	13
Contig N90 length (Mb)	2.775
Contig L90 count	38
Total number of scaffolds	30
Maximum scaffold length (Mb)	39.689
Minimum scaffold length (Kb)	57.08
Scaffold N50 length (Mb)	17.922
Scaffold L50 count	8
Scaffold N90 length (Mb)	13.388
Scaffold L90 count	17
Gap number	48
Gap length (bp)	3408
GC content (%)	34.15
Gene number	30,209
Repeat content (%)	41.65

### Gene and repeat annotations

Our assembly indicated that 141.4 Mb (41.65%) of the assembled genome consisted of repeated regions (Table 1). Long terminal repeats (LTRs) were the most abundant annotations, making up 18.76% of the genome, with Gypsy and Copia elements accounting for 9.44% and 9.15% of the genome, respectively, (Supplementary Data 1). *Copia* elements comprised a higher proportion of the genome of *S. brachista* relative to that found in other Salicaceae species examined (3.71–6.38%, Supplementary Table 7), indicating a considerable expansion of this element in the Cushion willow genome. For *Gypsy* the situation was more complex, with contraction occurring in the genomes of all three *Salix* species relative to estimates of genome representation in *Populus*, although this contraction was less marked in Cushion willow than in the other two *Salix* species examined. A phylogenetic analysis of *Copia* and *Gypsy* sequences across all six Salicaceae species revealed no evidence for any species-specific family members (Supplementary Figs. 4, 5), demonstrating that *Copia*/*Gypsy* sequence divergence predates species divergence.

We masked repeated regions and proceeded to annotate the genome using a comprehensive strategy combining evidence-based and *ab initio* gene prediction. Following the Maker pipeline^12^, we incorporated protein sequences from five Salicaceae species and *Arabidopsis thaliana*, and transcripts assembled from Cushion willow RNA-seq data (Supplementary Table 8**)**. In total, 30,209 gene models were identified, with an average CDS length of 1.31 kb and an average of 5.83 exons per transcript. The gene, exon and CDS region spanned 32.2% (109.3 Mb), 14.4% (49.0 Mb), and 11.7% (39.6 Mb) of the assembled genome, respectively. In addition, we identified 118 ribosomal RNA (rRNA) and 784 transfer RNA (tRNA) sequences. Most of the predicted proteins (97.4%) matched a predicted protein in public databases (Supplementary Table 9). The predominant biological processes among annotated gene models included cellular processes (7% of gene models) and responses to stimuli (5% of gene models) (Supplementary Fig. 6).

We used BUSCO^13^ to evaluate the quality of our gene annotation and found that 1383 out of the 1440 (96.1%) highly conserved core proteins in the Embryophyta lineage were present in our gene annotation, of which 1235 (85.8%) were single-copy genes and 148 (10.3%) were duplicated. For the remaining conserved genes, 143 (1.0%) had fragmented matches and 43 (2.9%) were missing.

### Comparative genomics and whole genome duplication events

To investigate the evolution of Cushion willow, we compared its genome to those of nine other Malpighiales species. These included six Salicaceae species – *Populus alba* var. *pyramidalis*, *P. trichocarpa, P. deltoides, P. euphratica, Salix purpurea, S. suchowensis*, and three other species - *Linum usitatissimum* (Linaceae), *Manihot esculenta* (Euphorbiaceae), and *Ricinus communis* (Euphorbiaceae).

We clustered the annotated genes of all ten Malpighiales species (Mlp-SP) into orthologous and paralogous groups using Orthfinder2^14^ and identified 13,793 and 25,467 orthologous clusters shared by the seven Salicaceae species (Sal-SP) and by the Mlp-SP group, respectively, of which 3040 and 518 were single-copy orthologs, respectively. Phylogenetic trees (using *Manihot esculenta* as outgroup) constructed from these single-copy orthologs confirmed that Salicaceae *s.str*., *Salix* and *Populus* are monophyletic clades (trees for Mlp-SP and Sal-SP groups are shown in Supplementary Figs. 7 and 2b, respectively), in accordance with our previous phylogenetic studies^15^.

Cushion willow predicted gene models were clustered into 19,032 gene families, with 12,163 singleton families and 6869 multi-copy families. Furthermore, we identified 13,793 gene families shared by Sal-SP species, 2262 species-specific families, and 5239 Cushion willow genes that did not cluster with orthologous clusters. Gene family analysis revealed that 866 of the 13,793 gene families shared among the Sal-SP species had undergone expansion in Cushion willow, 1446 gene families experienced contraction, and 132 gene families displayed signatures of rapid evolution (Fig. 2b).

To identify whole genome duplication (WGD) events, we used the density distribution of synonymous substitution rates per site (*K*s) between collinear paralogous genes, with the assumption that level of synonymous substitutions between two homologous sequences increases approximately linearly with time^16^. A total of 7464 syntenic blocks were identified in the Cushion willow genome (Fig. 2a). The total length of these blocks was 280.6 Mb (82.6% of the assembly), suggesting that the majority of the Cushion willow genome was duplicated during its evolution. The *K*s distribution of collinear gene pairs confirmed the common WGD event shared by both *Populus* and *Salix* genera^17^, indicated by a *K*s of around 0.3 and 0.25 in *Salix* and *Populus* species, respectively (Supplementary Fig. 8). The different *K*s values possibly reflect different nucleotide substitution rates in *Populus* and *Salix* lineages^18^.

We further performed synteny and collinearity analyses based on Salicaceae species with chromosomal scale genome assemblies. We found high synteny between *Salix* and *Populus* species (Supplementary Fig. 9a, b), supporting the hypothesis that these genera share a common WGD in their evolutionary history. Some distinct chromosomal rearrangement events were detected in chromosomes 4, 15, and 19 between Cushion willow and *S. purpurea* (Supplementary Fig. 9a). Chromosome 15 contains the sex-determination locus in *Salix*^19–21^, which raises the possibility of the sex determination region undergoing evolution in the *Salix* lineage. Interestingly, we found that chromosome 16 of Cushion willow involved conjunction of *P. trichocarpa* chromosome 16 and the lower portion of chromosome 1, while chromosome 1 of *P. trichocarpa* was a fusion of Cushion willow chromosome 1 and the lower portion of chromosome 16 (Fig. 2c, d). The same pattern of chromosomal restructuring was detected between Cushion willow and *P. deltoides* (Supplementary Fig. 9c) and was previously reported between *S. purpurea* and *P. trichocarpa*^19^, as well as between *S. suchowensis* and *P. trichocarpa*^22^. This indicates that the *Populus* and *Salix* lineages experienced different chromosomal fission and fusion events after they diverged from their common ancestor, as proposed by Hou et al.^22^.

### Temporal origin of Cushion willow

Divergence time estimation with fossil calibration based on 390 single-copy genes indicated when Cushion willow diverged from its close relative, *Salix souliei*, a dwarf willow (also placed in section *Lindleyanae*). *Salix souliei* is endemic to the THR and is confined to subnival regions above 4000 m, though is more widely distributed than Cushion willow, occurring in the QTP as well as the HDM and Himalaya. Our results indicate that the two species diverged ~11.73 Ma (6.83–18.34 Ma) (Supplementary Fig. 10), suggesting that Cushion willow must have evolved after the split with *S. souliei* and almost certainly less than 11.73 Ma. The Himalaya underwent progressive uplift during the middle Miocene (~13 Ma), with southern parts of the QTP likely reaching elevations comparable to those at present by mid-to-late Miocene (~5–15 Ma). In contrast, orogeny of the HDM occurred mainly after the late Miocene and reached peak elevation shortly before the Late Pliocene (~2.6 Ma, reviewed by Favre et al.^2^). It is feasible, therefore, that the ancestor of Cushion willow diverged from that of *S. souliei* in the Himalaya or QTP, and then dispersed to the eastern Himalaya or HDM, to give rise to Cushion willow. This kind of origin and diversification, possibly associated with tectonic events within the THR and the formation of climate-induced divergent habitats, has been suggested for several other species and species groups in the HDM, including *Saussurea* species, *Rhododendron* subg. *Hymenanthes*, and *Rhodiola* species (reviewed by Wen et al.^4^).

### Genomic footprints of alpine adaptation

We tested for enrichment of gene ontology terms (GO) and pathways (Kyoto Encyclopedia of Genes and Genomes, KEGG) by comparing the annotation content of expanded, rapidly evolved and species-specific genes in Cushion willow to the full complement of genes in its genome (Supplementary Table 10, Supplementary Data 2–4). Most notably, the top two enriched KEGG pathways among expanded gene families in *Salix brachista* were base excision repair and flavonoid biosynthesis (Supplementary Table 10). High elevations are characterized by high levels of UV radiation, which may cause DNA damage. Thus, given that base excision repair is a predominant DNA damage repair pathway^23^, it is possible that the expansion of gene families with functions related to DNA damage repair in Cushion willow were driven by natural selection in high elevation environments. This pathway was not enriched in expanded genes families in either of the two non-alpine *Salix* species examined (Supplementary Data 5). In addition, the finding that flavonoid biosynthesis, in particular anthocyanin synthesis, was over-represented is also consistent with adaptation to high elevation environments. Flavonoids, such as the common red, blue and purple anthocyanin pigments of plant tissues, are ubiquitous plant secondary products, which due to their UV-absorbing properties, have long been considered important in UV protection^24^. In Cushion willow, stems, branches and branchlets are typically purple in color, indicative of anthocyanin accumulation in these organs, which we confirmed by anthocyanin concentration measurements (Supplementary Fig. 11). Some GO-terms related to flavonoid biosynthesis were also enriched, including anthocyanidin 3-O-glucosyltransferase activity (GO:0047213) and anthocyanin-containing compound biosynthetic process (GO:0009718) (Supplementary Data 2). We further found that compared with the two non-alpine *Salix* species examined, Cushion willow tended to have a greater number of genes from gene families exhibiting the enriched terms mentioned above (Supplementary Data 6), thus reinforcing the idea that these functions may be involved in the adaptation of *S. brachista* to high elevations.

### Population diversification in Cushion willow

Cushion willow is mainly distributed in the HDM and its peripheral areas, including the eastern Himalaya (Bomi county) and two high mountains in the Central Yunnan Plateau (Eryuan county and Dongchuan county) (Fig. 1e). We collected and sequenced 77 individuals from 14 locations covering major habitats throughout the species distribution (Fig. 1e, Supplementary Data 7). From individuals of each population, we generated average 22.1-fold coverage (Supplementary Data 8 and 9). After stringent variant calling and filtering, we identified a total of 1,595,067 high-quality single-nucleotide polymorphisms (SNPs) with a minor allele frequency (MAF) >5% and missing frequency <20%, which were used for subsequent population-based analyses (Supplementary Table 11).

**Fig. 3:**
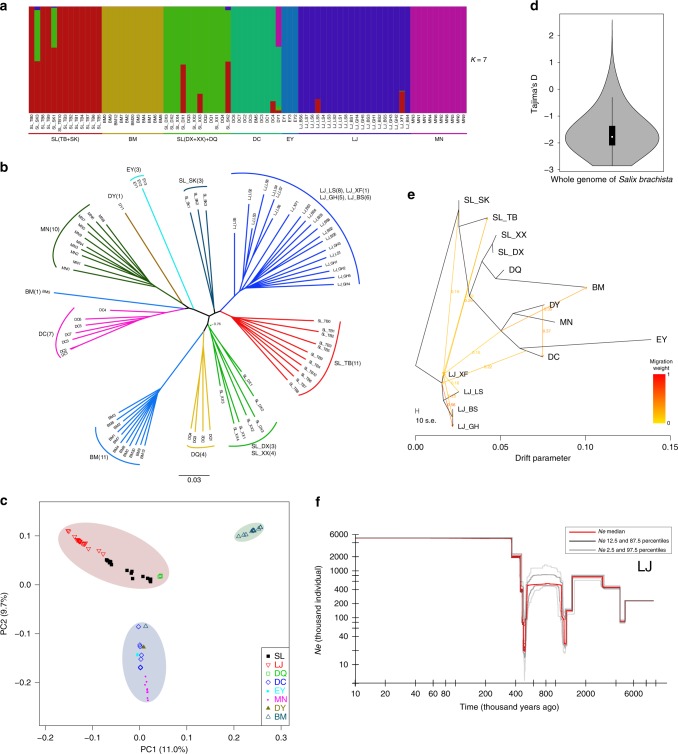
Population genetic strucure and demographic history. **a** Genetic structure of Cushion willow. The length of each colored segment represents the proportion of the individual’s genome from *K* = 7 ancestral genetic groups. The populations grouped by county individual identifiers are indicated along the *x*-axis. **b** Neighbor-joining phylogenetic tree of Cushion willow based on SNPs from whole-genome resequencing. Branch lengths are scaled to genetic similarity (*p* distance, see scale bar). **c** PCA plot of Cushion willow genetic variation. The fractions of the variance explained by eigenvector 1 and 2 are 11.0% and 9.7%, respectively. Each shaded area groups populations from the same region: pink for populations from northwest Yunnan at the center of the area sampled, blue for eastern peripheral populations, and green for the west most peripheral populations. **d** Violin plot of Tajima’s *D* in whole genome of *Salix brachista*. The width depicts a 90^0^-rotated kernel density trace and its reflection. Vertical black boxes denote the interquartile range between 25 and 75 percentiles, and the white point inside denotes the median. *n* = 168,546. **e** Detection of gene flow between Cushion willow populations. Lines represent gene flow, with arrows indicating the direction of gene flow. The horizontal scale bar at the bottom (drift parameter) shows a tenfold average standard error of the entries in the sample covariance matrix. The color scale shows the migration weight: red denotes strong gene flow, while yellow denotes weak gene flow. **f** Stairway plot showing historical changes in effective population size (*y*-axis) for LJ population with a generation time of 10 years. Red, dark gray, and light gray lines denote the medians, 12.5 and 87.5 percentiles, 2.5 and 97.5 percentiles of population sizes, respectively. The source data underlying Fig. 3d are provided as a Source Data file

Analysis of population structure revealed seven distinct clusters (*K* = 7) that reflected geographic divergence, and limited gene flow between certain populations (Figs. 1e and 3a, Supplementary Fig. 12). Only nine individuals were of mixed ancestry, representing possible hybrids between individuals assigned to different clusters. With *K* = 3, populations in the center of the distribution range (SL, LJ, and DQ) clustered together, indicating their very close relationship (Supplementary Fig. 12). Similar conclusions regarding genetic divergence within Cushion willow are drawn from a neighbor-joining (NJ) tree based on the ~1.6 million high-quality SNPs (Fig. 3b), and also from principal component analysis (PCA) (Fig. 3c). In the NJ tree, populations were resolved as robust monophyletic clades (bootstrap value of 100%), except for those located in Lijiang county (LJ_XF, LJ_LS, LS_BS and LJ_GH, Fig. 1b). These four populations, which occurred in close geographical proximity on the same mountain, formed one robust clade. They also formed a single cluster in structure analyses with *K* ranging from 4 to 10 (Supplementary Fig. 12), confirming their close relationship.

Interestingly, one individual from the western most population (BM5) clustered with the distant southeastern population (DC) some 792 km away, suggesting the possibility of long**-**distance dispersal (Fig. 3a, b). Willow seeds are covered in cottony hair and can be dispersed by wind over long distances^25^. Similarly, long-distance dispersal is feasible via pollen^26^. It is possible that long-distance wind dispersal of Cushion willow seeds and pollen is aided by the monsoon system in the HDM and the eastern Himalaya.

A population graph (Fig. 3e) inferred by Treemix^27^ indicated similar relationships between populations to those revealed in the NJ tree (Fig. 3b) and low levels of gene flow except between populations LJ_XF and LJ_GH (0.56) and DC and DY (0.37). The latter estimate should be treated with caution, given that the DY population comprised a single individual, possibly a hybrid of DC and MN parents, that may have established at the DY location following rare long-distance seed dispersal. Overall, gene flow tended to be more frequent between nearby populations than distant ones, as confirmed by a Pearson correlation coefficient analysis of population differentiation (fixation index, *F*_ST_) against geographical distance (*p* = 0.00000127) (Supplementary Data 10, Fig. 4a, b).

**Fig. 4:**
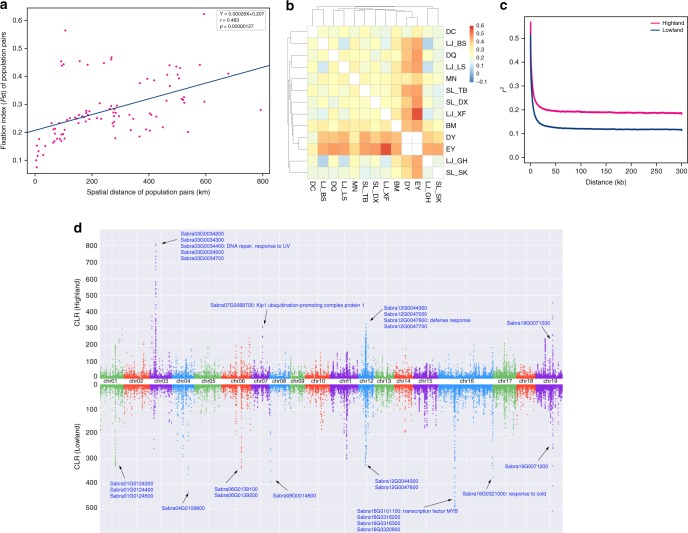
Signatures of selection in Cushion willow sampled from high and lower elevation sites. **a** Pairwise *F*_ST_ between populations (*y*-axis) as a function of the pairwise spatial distance between populations (*x*-axis). Each point represents one population pair. The solid blue line represents a significant linear relationship; parameter estimates for the linear regression, as well as Pearson’s correlation coefficient and associated *p*-value are given at the top-right corner of panel **b**. Heatmap of pairwise *F*_ST_ estimates between populations. **c** Patterns of linkage disequilibrium (LD). LD (*y*-axis) decays as a function of genomic distance between polymorphisms (*x*-axis) in high elevation (solid red line) and low elevation (solid blue line) Cushion willows populations. LD was measured by *r*^2^. **d** Manhattan plots of CLR among the high and low elevation Cushion willow plants of LJ populations. Dashed line indicates CLR value threshold. Genes located within the significant CLR peaks and corresponding annotations are denoted

### Divergence and demographic history related to climate change

To obtain an indication of the timing of intraspecific divergence within Cushion willow, we used fastsimcoal 2.6^28^, a coalescent simulation-based method, to infer the demographic histories and divergence times of six of the genetically divergent groups (populations) identified by structure analysis, including both centrally (DQ, SL, LJ) and peripherally (BM, MN, DC) distributed ones (Fig. 1e), based on their joint site frequency spectrum (SFS). Twenty-two alternative historical divergence models were fitted to the SFS (Supplementary Fig. 13) with a mutation rate of 1.01×10^−8^ mutations per site per generation assumed. Divergence times estimated for generation times of 3, 10, and 15 years (see Methods) approximated 0.42–2.1 Ma between peripherally and centrally distributed populations, which were older than those estimated between centrally distributed populations, which approximated 0.18–1.1 Ma (Supplementary Data 11). Thus, all splits of these populations (between which gene flow is very weak, regardless of geographical distance, Supplementary Data 11) were estimated to have occurred after the uplift of the HDM. Their divergence was most likely driven by isolation on different mountain summits (sky islands), and possibly adaptation to different subnival habitats and/or recurrent cycles of mixing, isolation and mixing (see below).

We employed the stairway plot method based on SNP frequency spectra to infer changes in population size (*Ne*) over time for six of the genetic groups identified by our structure analysis of Cushion willow. Again, a mutation rate of 1.01 × 10^−8^ mutations per generation for generation times of 3, 10, and 15 years was assumed. Similar curves for changes in population size within each genetic group were obtained across the three generation times (Fig. 3f, Supplementary Figs. 14–16); however, the number and timing of changes in population size differed among genetic groups. Such variation in demographic history between these groups is not unexpected given that glaciation of the THR region was highly variable, with extensive micro-climatic variation within individual mountain ranges^1,29^.

Importantly, all population bottlenecks were estimated to have occurred since the late Miocene and mostly since the beginning of the Quaternary except for two estimated to have occurred around 8 Ma with a generation time of 15 years. Each bottleneck event was usually followed by a population expansion, reflecting major climatic fluctuations since the late Miocene and particularly over the last 3 Ma^1^ (Fig. 3f; Supplementary Figs. 14–16). During glaciations, populations may have moved downslope to lower elevations, but nonetheless suffered marked reductions in effective size. During these periods, secondary contacts could have occurred between previously isolated sky island populations^30^, particularly neighboring ones, providing opportunities for hybridization and gene flow. This mixing would have ceased during interglacial periods when populations moved upslope and became isolated again on different sky islands. Recently, it has been shown that recurrent cycles of admixture and geographic isolation before complete reproductive isolation is achieved can potentially lead to high rates of speciation^31,32^. This process, termed the Mixing-Isolation-Mixing (MIM) model of speciation^31^, could have been particularly important in mountainous regions, such as the HDM, acting as a species pump to generate high alpine plant diversity^1^. Our finding that gene flow is not rare between neighboring populations of Cushion willow (Figs. 1e and 3a, e) suggests that recurrent cycles of admixture followed by isolation during the Pleistocene may have contributed to the notable genetic divergence recorded between populations of this species.

Despite fluctuations in population size, our stairway plot analysis indicated that most populations of Cushion willow expanded markedly during the Pleistocene (Supplementary Figs. 14–16). This result is also supported by negative values of Tajima’s *D* (−1.63, Fig. 3d) across the genome, a common signature of demographic expansion. There is only one population (SL_TB) that has smaller population size in most of the Pleistocene than now. This population increased in size early in the Pleistocene, but underwent significant reductions in size twice i.e. during the LGM and more recently. The DC population, which suffered a marked reduction in population size ~30 kya, nonetheless has a current size larger than the one it had at the start of the Pleistocene (Supplementary Figs. 14–16). Taken overall, our results suggest that the species generally coped well with environmental changes caused by HDM uplift and the glacial-interglacial cycles that have occurred since the late Miocene, and that the HDM has been a long-term refugium for Cushion willow.

### Selection on Cushion willow at high and lower elevations

Cushion willow generally grows at high elevations ~4000 m, although it can also be found in open sites on river banks at lower elevations (2760–3260 m) where forest is absent (Fig. 1). Significant environmental differences are evident between high and lower elevation sites (Supplementary Data 12) and plants occupying these different sites are, consequently, likely to be subject to different biotic and abiotic selective pressures. To examine this possibility, we scanned the genomes of plants from high and lower elevation LJ populations located on the same mountain, using the composite likelihood ratio (CLR) test implemented in SweepFinder2 based on unfolded SFS.

Focusing on regions of the genome with CLR scores higher than thresholds estimated using neutral coalescent simulations, we identified 2119 genomic regions (2 kb sliding windows) that encompassed ~4.2–4.6 MB, containing 381 genes, and 3545 genomic regions that encompassed ~7.09 MB, containing 544 genes, that were significantly selected in high and lower elevation plants, respectively (Fig. 4d, Supplementary Data 13). The selective role of these regions is also supported by significantly different Tajima’s *D* values (Supplementary Fig. 17) and different linkage disequilibrium patterns (Fig. 4c). Although we did not detect significant GO terms with corrected *p* value <0.05 for these selected genes, some notable peaks were detected across the genome characterized by consecutive blocks with very high CLR, indicating putative selective sweeps (Fig. 4d). Some of the genes within these peaks could, therefore, possibly be important in alpine adaptation. For example, the *Sabra3G0034400* gene located in the very notable peak of chromosome 3 in high elevation LJ populations has GO annotations that include DNA repair (GO:0006281), DNA damage induced protein phosphorylation (GO:0006975), while the *Sabra12G0047600* gene within the peak of chromosome 12 has GO annotations related to defense responses, such as defense response to bacterium (GO:0042742), and regulation of salicylic acid mediated signaling pathway (GO:2000031). In addition, of four genes located in the highest peak for lower elevation plants, *Sabra16G0101100* is a transcription factor MYB with GO annotations that include cellular response to DNA damage stimulus (GO:0006974) and response to cold (GO:0009409).

## Discussion

We present here a high-quality, chromosome-scale reference genome for Cushion willow (*Salix brachista*). Whole-genome resequencing-based population genetic analyses revealed that sky island populations of Cushion willow in the HDM located on high mountains isolated from others were genetically divergent and poorly connected by gene flow, despite occasional long-distance dispersal events. Population differentiation was positively correlated with spatial distance. Genomic analysis further indicated that high and lower elevation populations of Cushion willow face different types of natural selection, and detected some genes inferred to be important to adaptation to the different environmental conditions experienced at these sites. Genetic differentiation of geographically isolated populations in combination with the different strengths of natural selection occurring between high and lower elevation populations are likely to have contributed to the divergence of Cushion willow populations in the HDM. Our results are consistent with the hypothesis that diverse isolated heterogeneous habitats on sky islands, together with climatic fluctuations that have possibly caused recurrent cycles of admixture and isolation between some populations (MIM cycles), were important drivers of intraspecific population diversification in Cushion willow, and by extension, other alpine species with similar distributions and histories in the HDM. We therefore speculate from our results that geographic isolation and environmental variation associated with elevation resulting from the uplift of the THR, in combination with the effects of climate fluctuation since the late Miocene, have contributed to the high species richness observed in the HDM by driving intraspecific population divergence, a precursor of speciation.

## Methods

### Plant material

The sequenced individual of Cushion willow was a female plant collected from Tianbao Mountain, Shangri-La Country, Yunnan Province, China (N27.60521º, E99.89144º), growing on a scree slope at an elevation of 4102m. Using tissue culture, we raised plantlets from this individual to facilitate isolation of long fragments of DNA.

### Estimation of genome size and ploidy

The genome size was estimated to be ~400 Mb by 17-*k*-mer analysis (organelle-derived reads were removed) based on PCR-free Illumina short reads. We found large amount of high frequency (>1 × 10^5^) *k*-mers in the sequenced reads (1705 kinds of 17-*k*-mers sum to ~1.626 Gb), which might originate from highly repetitive regions within centromeres and telomeres, for example. These high frequency *k*-mers amounted to ~47.8 Mb which, if excluded, resulted in a genome size estimated to be ~350 Mb. The genome size estimated by 17 flow cytometry measurements was 420.83 ± 8.26 Mb using *Zea mays* L. as a standard (Supplementary Table 1, Supplementary Fig. 18). The sequenced individual of Cushion willow was determined as diploid with 38 chromosomes, using a cytological method described previously^33^ (Supplementary Note 1).

### Genome sequencing

High-quality genomic DNA was extracted from plantlets and fresh leaves using a modified CTAB method^34^ for SMRT cell and Illumina sequencing, respectively. For Nanopore sequencing, PromethION libraries were prepared following the Oxford Nanopore 1D Genomic DNA by ligation (SQK-LSK109) - PromethION (version GDE_9063_v109_revD_04Jun2018) protocol, and sequenced on a Nanopore PromethION platform. For SMRT sequencing, genomic DNA was fragmented by g-Tube (Covaris, USA) and 18–22 kb DNA fragments were further purified using AMPure magnetic beads (Beckman Coulter, USA). We obtained 50 µg of high-quality genomic DNA with a median fragment size of 20 kb, from which we prepared sequencing libraries using the DNA Template Prep Kit 4.0 V2 (Pacific Biosciences, USA) following the manufacturer’s protocols (Pacific Biosciences, USA). We sequenced libraries on a PacBio RS II platform. For Illumina sequencing of whole-genome resequencing of individuals, short-insert libraries were constructed following the manufacture’s protocol (Illumina Inc., USA). Illumina PCR-free sequencing was also performed for the whole-genome sequenced individual, and library preparation was performed following the original Illumina TruSeq DNA PCR-free LPP (revision A, January 2013, low sample with 550 bps insert size) and sequenced on an Illumina Hiseq X Ten platform.

### RNA extraction and library preparation

To assist gene annotation, we performed RNA sequencing. We harvested whole plants of the whole-genome sequenced individual from tissue culture plantlets, including leaf, stem, and root tissues, and froze them in liquid nitrogen upon collection. Fresh leaves of four *Salix* species (*S. tetrasperma*, *S. babylonica*, *S. souliei* and *S. wallichiana*) were also collected for the purpose of divergence time estimation (Supplementary Table 12). We performed RNA isolation immediately after collection. Total RNA was extracted with Trizol reagents and mRNAs were purified using a NEBNext Ultra RNA Library Prep Kit for Illumina (New England Biolabs Inc.). In total we pooled ca. in all, 2 μg of RNA for each sample to prepare the RNA-seq libraries, which we sequenced on the Illumina Hiseq X Ten platform.

### Hi-C library preparation and sequencing

The Hi-C library was prepared following a standard procedure^35^. In brief, in situ cross-linked DNA from 700 ng of high molecular-weight genomic DNA was extracted and digested with a restriction enzyme. The sticky ends of the digested fragments were biotinylated, diluted, and ligated to each other randomly to form chimeric junctions. Biotinylated DNA fragments were enriched and sheared to a size of 300–500 bp for preparing the sequencing library. Libraries were sequenced on a HiSeq X Ten platform (Illumina).

### Genome assembly

We used SMARTdenovo v1.0 (https://github.com/ruanjue/smartdenovo), wtdbg2 (https://github.com/ruanjue/wtdbg2) assemblers with ONT reads corrected by Canu^11^ for de novo assembly. We used the following options as inputs to SMARTdenovo: -c l to generate a consensus sequence, -J 5000 to remove sequences <5 kb and -k 20 to use 20-mers, as advised by the developers for large genomes. We then selected the ‘best’ assembly, which was SMARTdenovo with Canu correction, based on contiguity metrics, including N50 or cumulative size (Supplementary Table 13). Since ONT reads contain systematic errors in homopolymeric regions, we mapped PacBio long reads to the assembled genome using pbmm2 (https://github.com/PacificBiosciences/pbmm2) and polished two iterations by arrow (https://github.com/PacificBiosciences/GenomicConsensus). We then mapped Illumina reads to the polished genome assembly by BWA-MEM (https://github.com/lh3/bwa) and polished five times using Pilon^36^.

### Scaffolding with Hi-C data

We corrected mis-joins, order and orientation of a candidate chromosome-length assembly automatically generated by 3d-DNA pipeline (https://github.com/theaidenlab/3d-dna), followed by anchoring contigs in the assembly. We then mapped the clean Hi-C reads to the assembly with Juicer (https://github.com/aidenlab/juicer). The assembly was then manually reviewed and refined for quality control and interactive correction using Juicebox Assembly Tools (https://github.com/aidenlab/Juicebox). In order to decrease the influence of chromosome interactions and improve the chromosome-scale assembly further, we re-scaffolded each chromosome with 3d-DNA separately, followed by manual refinement with Juicebox. We therefore anchored 19 chromosomes (337.28 Mb, 99.32%).

### Optimization of genome assembly

The genome assembly was gap closed twice using LR_Gapcloser (https://github.com/CAFS-bioinformatics/LR_Gapcloser). We then compared the un-clustered contigs, as well as clustered contigs in Hi-C scaffolding. Contigs with identity of more than 90% (and 99% coverage for shorter contigs) were regarded as redundant sequences and were removed. Redundant sequences were mainly repeated sequences or sequences from different haplotypes. We also used the un-clustered contigs in Hi-C scaffolding to blast against the NCBI-NT database and found no contaminated contigs. Overall, we removed 80 redundant contigs, representing a total of 12.8 Mb. Illumina short reads were then mapped to the genome assembly using BWA-MEM before polishing twice. The final assembly of Cushion willow was 339.59 Mb in size with a contig N50 of 9.52 Mb, including 19 chromosome-length scaffolds, 9 un-clustered contigs, as well as chloroplast and mitochondrial genomes.

### Transcriptome assembly

RNA-seq reads were preprocessed by Cutadapt^37^ to remove contaminating sequences from adapters and low base quality sequences. We mapped the RNA-seq reads to genome by HiSat2 (https://github.com/infphilo/hisat2), and used StringTie (https://github.com/gpertea/stringtie) for referencing-guided assembly and Trinity^38^ or genome-guided de novo assembly. Assembled transcripts were combined, with redundant ones removed (i.e. transcripts with identity of 99% and coverage of 99%), using CD-HIT (https://github.com/weizhongli/cdhit).

### Characterization of repetitive sequences

Repeat families found in the genome assemblies of Cushion willow were independently identified de novo and classified initially using the software package RepeatModeler^39^. RepeatModerler depends on the programs RECON and RepeatScout for de novo identification of repeats within the genome assemblies. The repeat library produced by RepeatModeler was then used in the program RepeatMasker^39^ to discover and identify repeats in the genome assemblies.

### Gene annotation

The Augustus^40^ ab initio gene finder was used to identify gene models for Cushion willow genome assemblies. The gene finder was trained using protein models from six species: *Arabidopsis thaliana, Populus trichocarpa, P. euphratica*, *P. deltoides, Salix suchowensis*, and *S. purpurea* v1.0 (sources of genome datasets used are listed in Supplementary Table 14). The package MAKER^12^ with Augustus was then used to annotate genes in the repeat-masked reference genome. Finally, predicted gene models with abnormal frame (no start or stop codon, or an inside stop codon) were excluded. The programs tRNAScan-SE^41^, RNAmmer^42^ were used to predict tRNA and rRNA, respectively, and other ncRNAs were identified by searching against the Rfam database (http://eggnogdb.embl.de/). Pseudogene identification was conducted using the pipeline described by Zou et al.^43^. Predicted genes were aligned to proteins in SwissProt and TrEMBL databases (https://www.ebi.ac.uk/uniprot) using BLASTp (best hit with E <10^−5^) to determine the best matching alignments. Motifs and functional domains were identified using InterProScan (http://www.ebi.ac.uk/InterProScan) by searching against publicly available protein databases including ProDom, PROSITE, Pfam, PRINTS, and SMAR. We also mapped the predicted Cushion willow genes to their corresponding KEGG pathways, with the best hits for each gene used to establish GO and KEGG pathway.

### Ortholog identification and WGD analysis

We used Orthofinder2^14^ to identify homologous gene clusters (including ortholog and paralog clusters, sources of genome datasets used are listed in Supplementary Table 14). MCScanX^44^ was then used for collinear analysis. We identified homologous clusters for the aforementioned Mlp-SP and Sal-SP species groups and constructed two phylogenetic trees (using *Manihot esculenta* as outgroup) based on 518 and 3040 single-copy orthologs shared by Mlp-SP and Sal-SP groups, respectively. MUSCLE^45^ was used to align homologous gene pairs, before transforming aligned protein sequences into codon alignment by PAL2NAL^46^. The *K*a, *K*s values of homologous gene pairs were calculated by *K*a*K*s Calculator^47^ with Yang-Nielsen (YN) model. WGD events were inferred from the distribution of *K*s.

### Divergence time estimation

Divergence times were estimated between 11 Salicaceae species, including a closely related species of Cushion willow, *Salix souliei* based on the amino acid sequences of 390 single-copy orthologous genes shared by these species. A maximum-likelihood tree was constructed by RaxML with the GAMMA BLOSUM62 model. Divergence times were estimated by MCMCtree of the PAML 4.0 package^48^ using the approximate likelihood method with independent substitution rate (clock = 2) and GTR substitution model, with samples drawn every 10 iterations until completion of 10^7^ iterations. Overall, we ran 1.1 × 10^8^ iterations and discarded 10^7^ iterations as burn-in. To check for convergence to the stationary distribution, each analysis (including the codeml step) was run in duplicate with results compared between runs. Reliable fossils of *Populus tidwellii* (most likely representing a member of the stem lineage leading to *Populus* and *Salix*) and *Pseudosalix* (an extinct, early divergent member of the stem lineage of *Populus* and *Salix*)^49^, both occurred in early Middle Eocene (~48 Ma). Because this divergence time has also been estimated to be 52 Ma^18^, the root age of the tree was calibrated to 48–52 Ma.

### Gene family analysis

Gene families were identified based on protein-coding sequences of *Populus trichocarpa, P. alba* var. *pyramidalis*, *P. euphratica, P. deltoides, Salix suchowensis*, and *S. purpurea*. We generated a maximum-likelihood gene tree using RaxML^50^ with the GTRGAMMA model based on 3040 single-copy orthologous genes from the aforementioned Sal-SP. We then determined expanded and rapidly evolved orthologous gene families using CAFE^51^ with default parameters based on the gene tree inferred among the 13,793 shared gene families of the Salicaceae species. The lineage/species-specific gene families were determined according to the presence or absence of genes for a given lineage/species.

### GO and KEGG enrichment analysis

GO and KEGG enrichment analyses were performed using the *R* package clusterProfiler^52^ to identify significantly enriched terms. We used expanded/rapidly evolved/species-specific genes as input and all genes of the whole genome annotated with GO and KEGG terms as background for each species examined. The resulting *p* values were corrected for multiple comparisons using the method of Benjamini and Hochberg.

### Anthocyanin measurement

Anthocyanin content of Cushion willow branchlets of greenhouse LJ_BS (elevation: 2950 m) and LJ_LS (elevation: 3950 m) plants (6 individuals for each group) was measured according to Teng et al.^53^ (Supplementary Note 2). Two measures of absorbance of extracts were made at 535 and 650 nm by spectrophotometry with anthocyanin quantity equaling (A535-A650) per gram fresh weight.

### Whole-genome resequencing and SNP calling

We obtained plant material of individuals from 14 wild populations in localities across the distribution area of Cushion willow (Supplementary Data 7, Fig. 1). One of these individuals was from Tianbao Mountain, Shangri-La Country, used to produce the assembled reference genome sequence as described above. The genomes of the remaining 77 individuals were resequenced using the Illumina HiSeq X Ten platform. Paired-end reads from each of these individuals were aligned to the assembled Cushion willow reference genome using BWA with default parameters. To minimize the influence of mapping bias, we further discarded the following sites: (1) sites with extremely low (<300× across all samples, i.e. less than an average of 4× per sample) or extremely high coverage (>3000×, or approximately twice the mean depth at variant sites) across all samples after investigating the coverage distribution empirically; (2) sites with a high number of reads (on average >8 reads per sample) with mapping score equaling zero; (3) sites located within repetitive sequences as identified using RepeatMasker^39^; and (4) sites belonging to organelle genomes, and the 9 contigs that were not anchored on the 19 super-scaffolds. 196,214,630 sites were retained after the above steps. SNP calling was then conducted using Freebayes^54^ to call sites with Quality >20. We performed several filtering steps to minimize SNP calling bias using VCFtools^55^ and BCFtools^56^ and to retain only high-quality SNPs. These included: (1) removal of SNPs at sites not passing all aforementioned filtering criteria; (2) retention of only bi-allelic SNPs with a distance of >5 bp away from any indels; and (3) treating genotypes with quality score <20 and depth <5 as missing. Finally, we removed SNPs with missing rate >20%, which resulted in 6,524,760 SNPs. The 1,595,067 SNPs with minor allele frequency <5% were deemed as high**-**quality SNPs and were used for downstream analysis. SNPs were annotated using ANNOVAR (https://vatlab.github.io/vat-docs/documentation/tutorials/annovar/).

### Genetic structure and gene flow analysis

Genetic structure based on SNP variation among the sequenced 78 genotypes was analyzed using fastSTRUCTURE^57^ with 2–15 ancestral clusters (*K*) and the value *K* = 7 was selected using the chooseK.py function. A neighbor-joining tree was constructed using MEGA7^58^ with *p*-distance method and the clade supports were calculated using 1000 bootstraps. Principal component analysis (PCA) was performed with GCTA^59^. To estimate gene flow between populations of Cushion willow, we used Treemix v1.13^27^ conducted on the ~1.6 M high-quality SNP dataset, with the settings -se -bootstrap -k 500 -m, and values for (−m) ranging from 1 to 10.

### LD analysis and calculation of population parameters

We estimated LD decay based on the coefficient of determination (*r*^2^) between any two loci using PopLDdecay (https://github.com/BGI-shenzhen/PopLDdecay). Tajima’s *D*, *F*st, and *θ*_*π*_ were calculated from sample allele frequency likelihoods in ANGSD over non-overlapping 2-kb windows. A summary of results is listed in Supplementary Data 14.

### Site ancestral state estimation

We inferred the site ancestral state (SAS) of Cushion willow following the Enredo-Pecan-Ortheus pipeline^60^, a probabilistic alignment-based method. We constructed a NJ tree using MEGA, including Cushion willow, *S. purpurea* [https://www.ncbi.nlm.nih.gov/sra/?term=SRR3927001], *S. suchowensis* [https://www.ncbi.nlm.nih.gov/sra/?term=SRR8255666], and *S. viminalis* [https://www.ncbi.nlm.nih.gov/sra/?term=ERR1558611], with *Populus trichocarpa* as outgroup to define the root. We identified 249,212,838 high confidence and 53,624,778 low confidence sites, yielding 302,837,616 (89.2%) sites as ancestral state in the assembled Cushion willow genome, which was used to estimate unfolded SFS and polarize SNPs.

### Divergence and demographic history of populations

We inferred the divergence times of six population pairs of Cushion willow (Supplementary Data 11) using a coalescent simulation-based method implemented in fastsimcoal 2.6, which compared 22 demographic models (Supplementary Fig. 13) using SFS^61^. Two-dimensional joint SFS (2D-SFS) was constructed from posterior probabilities of sample allele frequencies by realSFS implemented in ANGSD^62^. Only reads with mapping quality >30 and bases with quality score >20 were used in the analysis. To minimize the influence of mapping bias, sites not passing the aforementioned filtering criteria were removed. Sites within gene regions were also removed. 102,494,073 sites were therefore retained. A total of 100,000 coalescent simulations were used for the estimation of the expected 2D-SFS and log-likelihood for a set of demographic parameters in each model. Mutation rate (*μ*), shown to be related to genome size in a variety of organisms including *Populus trichocarpa*, was calculated following Lynch (2010)^63^ and estimated to be 1.01 × 10^−8^ mutations per site per generation. All parameter estimates were global ML estimates from 50 independent fastsimcoal2 runs, with 100,000 simulations per likelihood estimation and 40 cycles of the likelihood maximization algorithm. The best model was identified through the Akaike’s information criterion; simulated datasets were compared with the observed site frequency spectra to evaluate the fit of the best demographic model. Parameter confidence intervals of the best model were obtained by 100 parametric bootstraps, with 50 independent runs in each bootstrap. Divergence times were estimated for generation times of 3, 10, and 15 years^64–66^.

The demographic history of six of the genetic groups identified by structure analysis was examined using a stairway plot with 200 bootstrap iterations, to infer changes in population size over time using SFS^61^. The EY group was excluded because it comprised only three individuals. Ten individuals, which did not cluster according to geography and were admixed, were also excluded from analysis. SFS was estimated using ANGSD based on SAS as above. The same mutation rate as above was used in analyses, with analyses repeated across the same range of generation times, i.e. 3, 10, and 15 years.

### Environment heterogeneity analysis

We obtained four bioclimatic factors, annual mean air temperature, air temperature annual range, annual precipitation, and precipitation seasonality, from Worldclim (https://www.worldclim.org/) with spatial resolution of 1 km^67^ (Supplementary Data 15). Terrestrial ecosystem heterogeneity data of global habitat with resolution of 1 km based on textural features of Enhanced Vegetation Index (EVI) imagery^68^ was obtained. We chose eight environmental factors, coefficient of variation (CV), dissimilarity, evenness, homogeneity, range, Shannon, Simpson, and standard deviation (Std) from the dataset, and conducted independent-samples *t* tests on these variables to examine environmental differences between lower (2760–3230 m) and high (3800–4430 m) elevation sites occupied by Cushion willow (Supplementary Data 12).

### Selective sweep analysis

We used SweepFinder2^69^, a composite likelihood ratio statistic (CLR) to search for signs of selective sweeps in populations from high (LJ_XF and LJ_LS, nine individuals) versus lower (LJ_GH and LJ_BS, nine individuals) elevations on the same mountain in Lijiang County (LJ) based on SAS. The small fraction of SNPs that could not be polarized was excluded from further analysis. We only used sites that were polymorphic or that represented fixed substitutions in each group of populations to scan for sweeps^70^. Sweepfinder2 was run with a grid size of 2 kb. CLR scores were merged into sweep regions if neighboring scores exceeded CLR scores of 100. To examine the threshold for CLR scores, we used neutral simulations obtained from the best demographic model between the high and lower elevation LJ populations, which was model 18 (Supplementary Fig. 13) as inferred by fastsimcoal2.6^28^. We then simulated 100 datasets for each of the high and lower elevation populations by fastsimcoal2.6 for sweep analysis using SweepFinder2. The 95th quantile of values composed by maximum CLR score of each simulation dataset was used as CLR threshold for identifying sweep region, which was 38.9 and 30.9 in high and lower elevation populations, respectively.

### Reporting summary

Further information on research design is available in the Nature Research Reporting Summary linked to this article.

## Supplementary information

Supplementary Information

Peer Review

Reporting summary

Description of Additional Supplementary Files

Supplementary Data 1

Supplementary Data 2

Supplementary Data 3

Supplementary Data 4

Supplementary Data 5

Supplementary Data 6

Supplementary Data 7

Supplementary Data 8

Supplementary Data 9

Supplementary Data 10

Supplementary Data 11

Supplementary Data 12

Supplementary Data 13

Supplementary Data 14

Supplementary Data 15

Source data

## Data Availability

Data supporting the findings of this work are available within the paper and associated Supplementary Information files. A reporting summary for this Article is available as a Supplementary Information file. The datasets generated and analyzed during the current study are available from the corresponding author upon request. The whole genome shotgun project for Cushion willow is deposited in DDBJ/ENA/GenBank and GWH (Genome Warehouse) under the accessions VDCV00000000 and GWHAAZH00000000, respectively. All raw reads generated in this study are deposited in the NCBI database under BioProject accession PRJNA472210 (Cushion willow related raw reads) and PRJNA545026 (RNAseq raw reads for *Salix souliei, S. tetrasperma, S. wallichiana*, and *S. babylonica*). The source data underlying Fig. 3d, Supplementary Figs. 11d and 17, and Supplementary Table 1 are provided as a Source Data file.
