# Supplementary material for: Genome-wide analysis of Cushion willow provides insights into alpine plant divergence in a biodiversity hotspot
Source: Nat Commun. 2019 Nov 19;10:5230. doi: 10.1038/s41467-019-13128-y (PMC6864086; doi:10.1038/s41467-019-13128-y)
Supplement: Supplementary file 1 — Supplementary Information [file 41467_2019_13128_MOESM1_ESM.pdf]

Genome-wide analysis of Cushion willow provides insights into alpine plant divergence in a biodiversity hotspot

Chen *et al.*

Supplementary Note 1: Cytogenetic studies

Root tips of the whole-genome sequenced individual of Cushion willow were collected and fixed in Carnoy's solution (ethanol and acetic acid, 3:1 v/v). Fixed root tips were digested in 1% pectolytic enzyme mixture: 0.3% (w/v) cellulase, 0.3% (w/v) pectolyase, 0.3% (w/v) cytohelicase. Root tips were hydrolyzed in 1 mol/L HCl at 60 ºC for 10-15 min, and then washed with distilled water, dyed with carbolfuchsin and squashed preparations were made in a drop of 45% acetic acid for observation.

Supplementary Note 2: Anthocyanin measurement

Branchlets of greenhouse LJ_BS (elevation: 2950 m) and LJ_LS (elevation: 3950 m) Cushion willow (6 individuals for each group) were collected and stored in liquid nitrogen. Frozen, homogenized branchlets (20 mg) were extracted for 1 d at 4°C in 1 mL of 1% (v/v) HCl in methanol. The mixture was centrifuged at 13,000 xg for 15 min and the absorbance of the supernatant was measured at 535 and 650 nm. The amount of anthocyanins was defined as the (A535-A650) per gram fresh weight (FW).

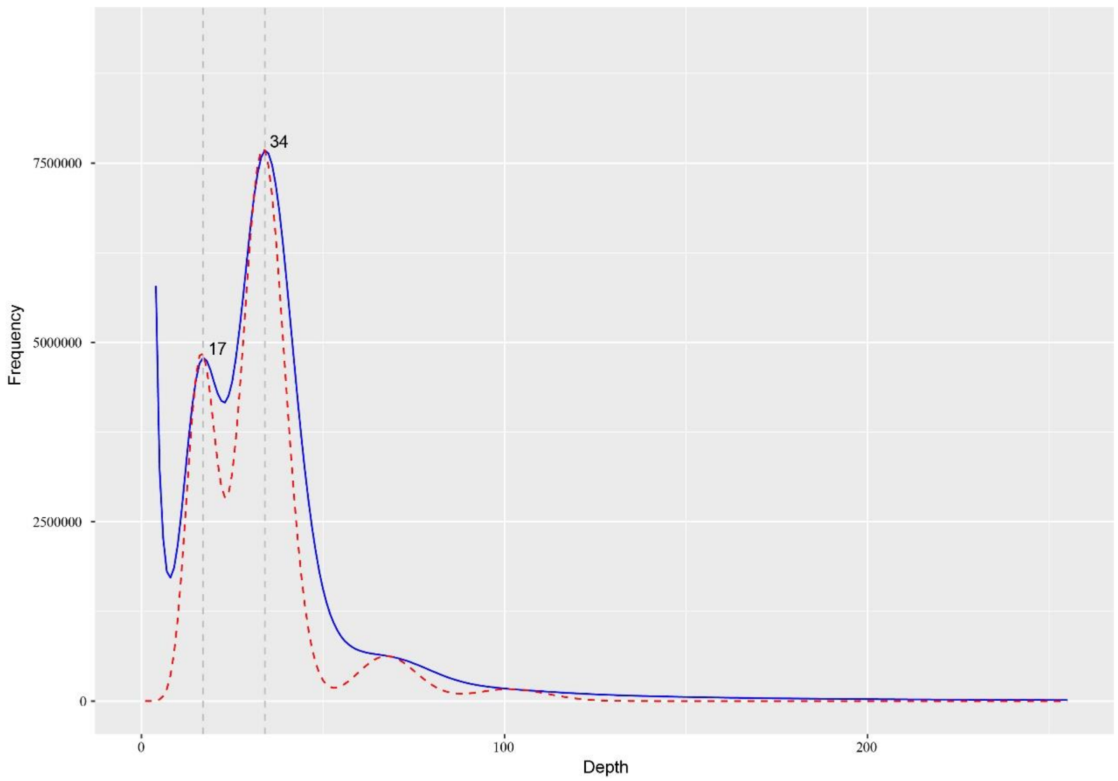

Supplementary Figure 1. The 17-mer distribution of Illumina short-read data. The x-axis shows *K*-mer abundance. The y-axis shows the number of *K*-mer at a given abundance.

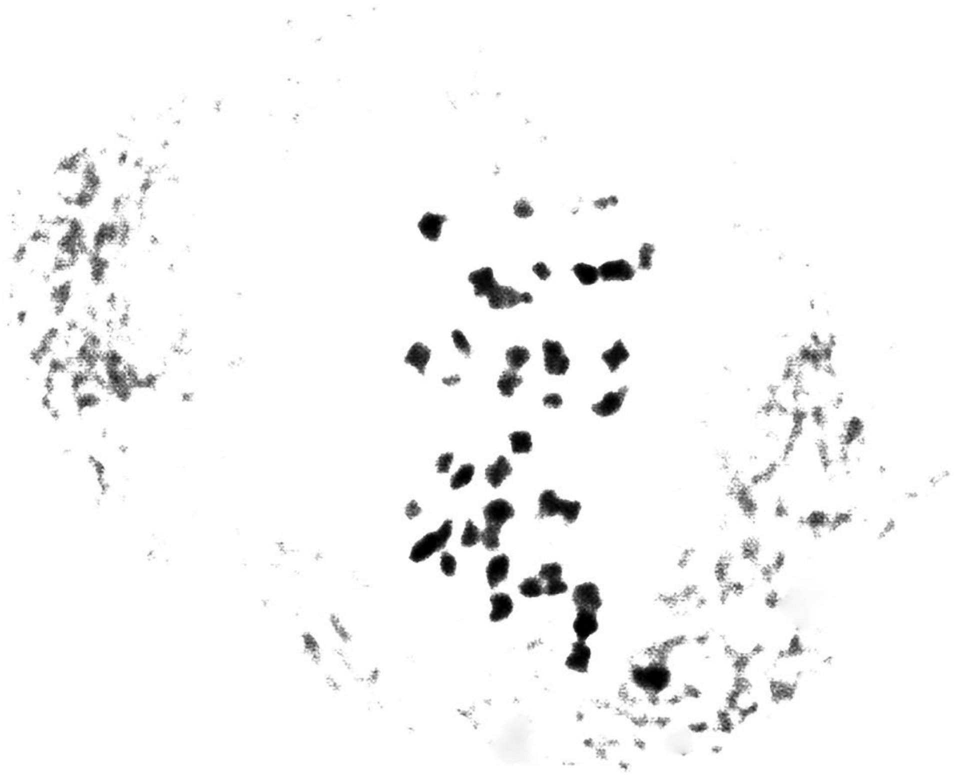

Supplementary Figure 2. Mitotic metaphase chromosomes of the sequenced individual of *Salix brachista*. The chromosome number is 38, indicating the sequenced individual is diploid.

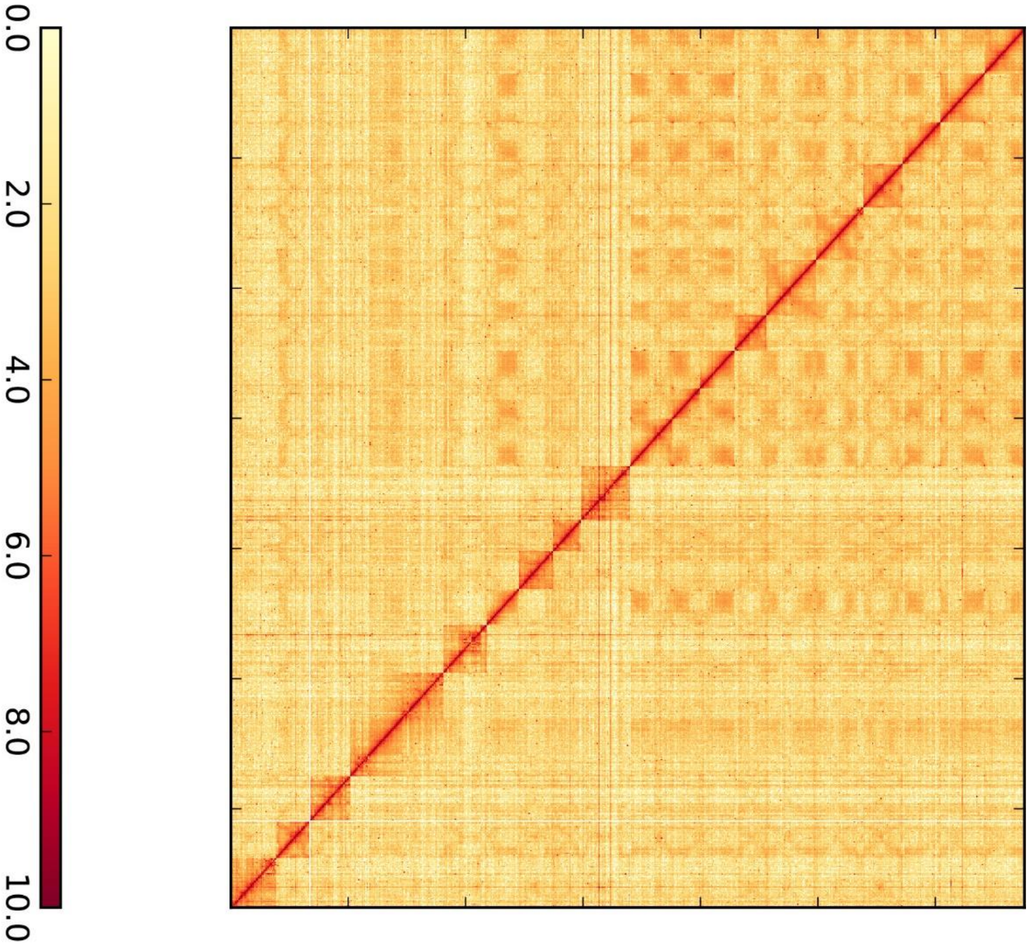

Supplementary Figure 3. Hi-C linkage density heat map of assembled contigs. Showing the the contigs were clustered into 19 pseudo-chromosomes (2n=38).

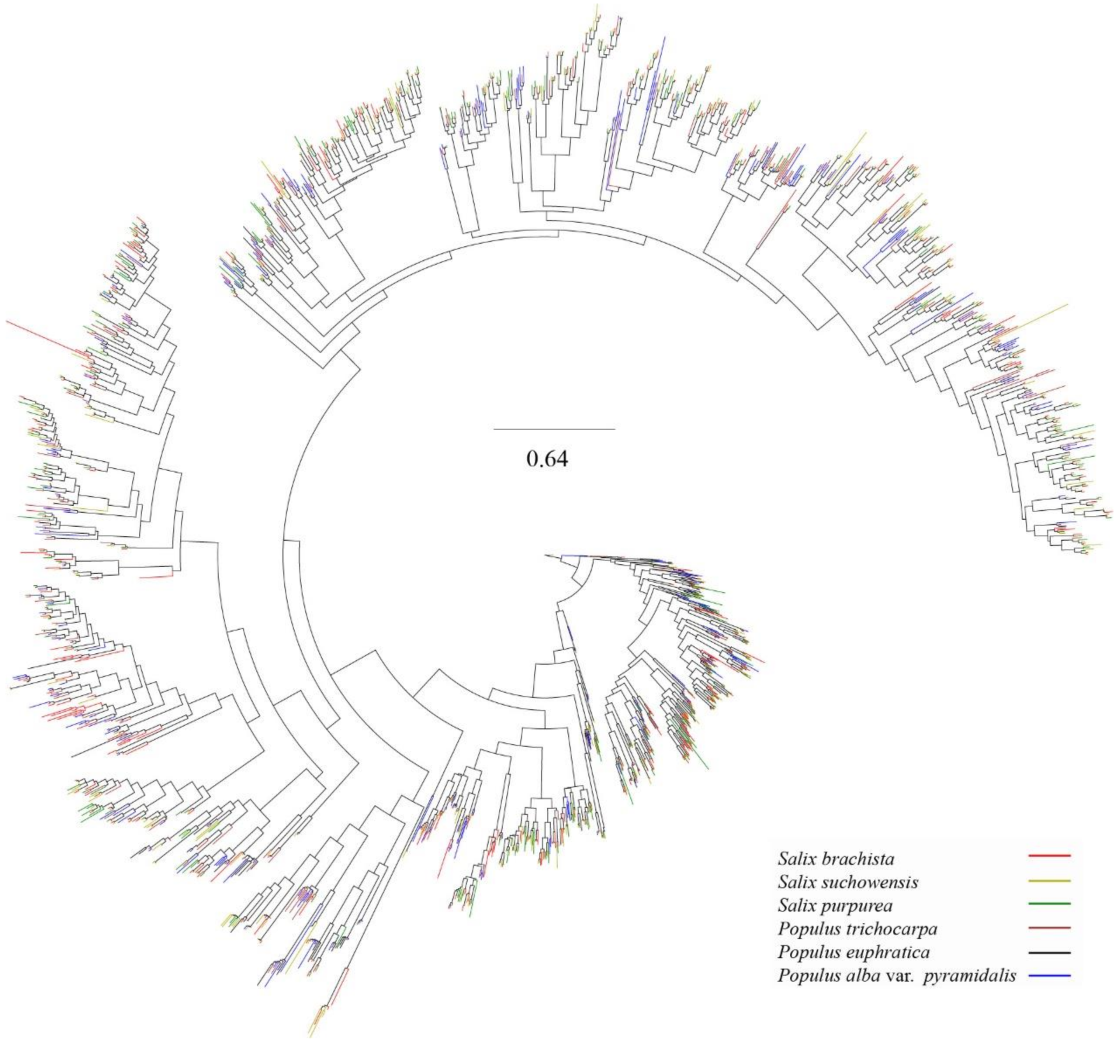

Supplementary Figure 4. Neighbor joining tree of LTR/Copia retrotransposon repeat family.

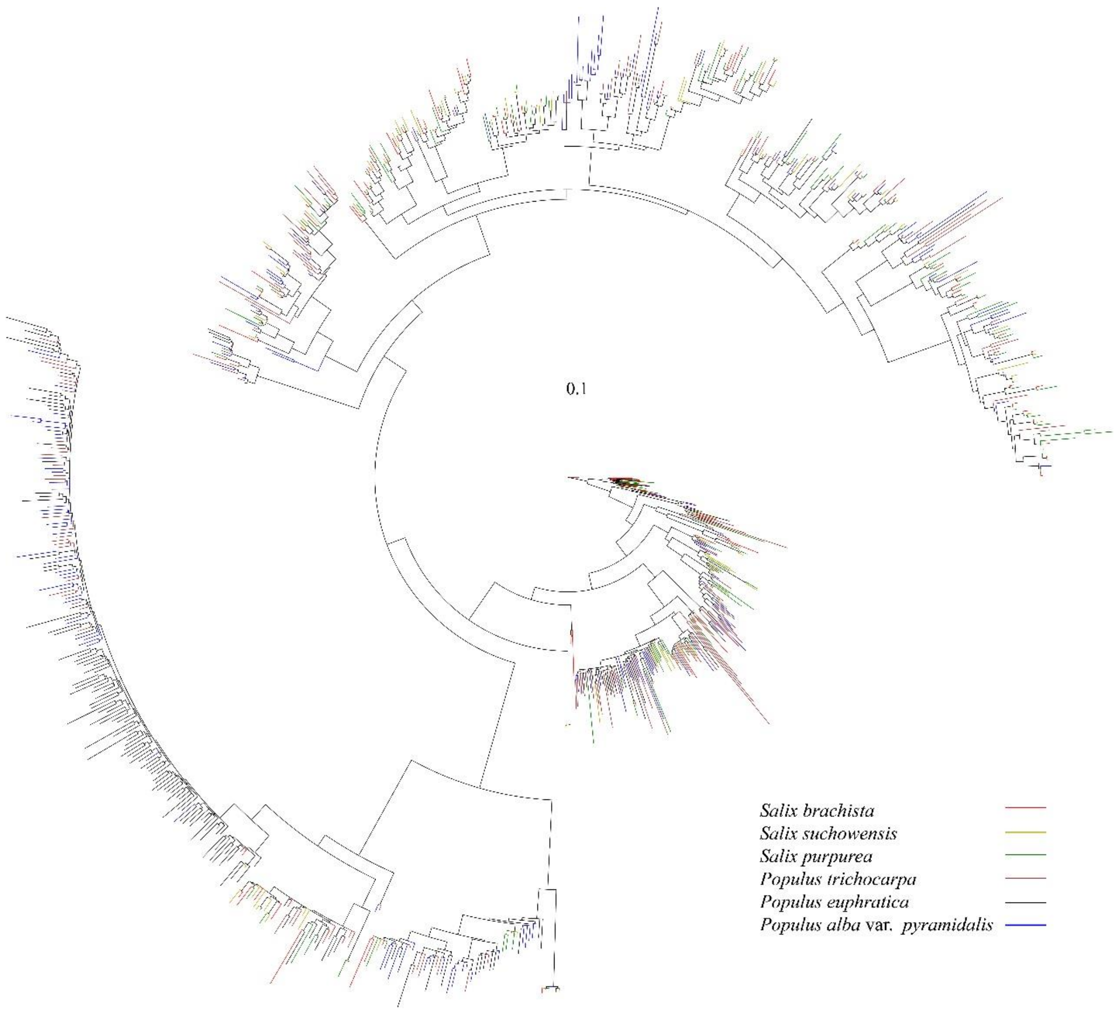

Supplementary Figure 5. Neighbor joining tree of LTR/Gypsy retrotransposon repeat family.

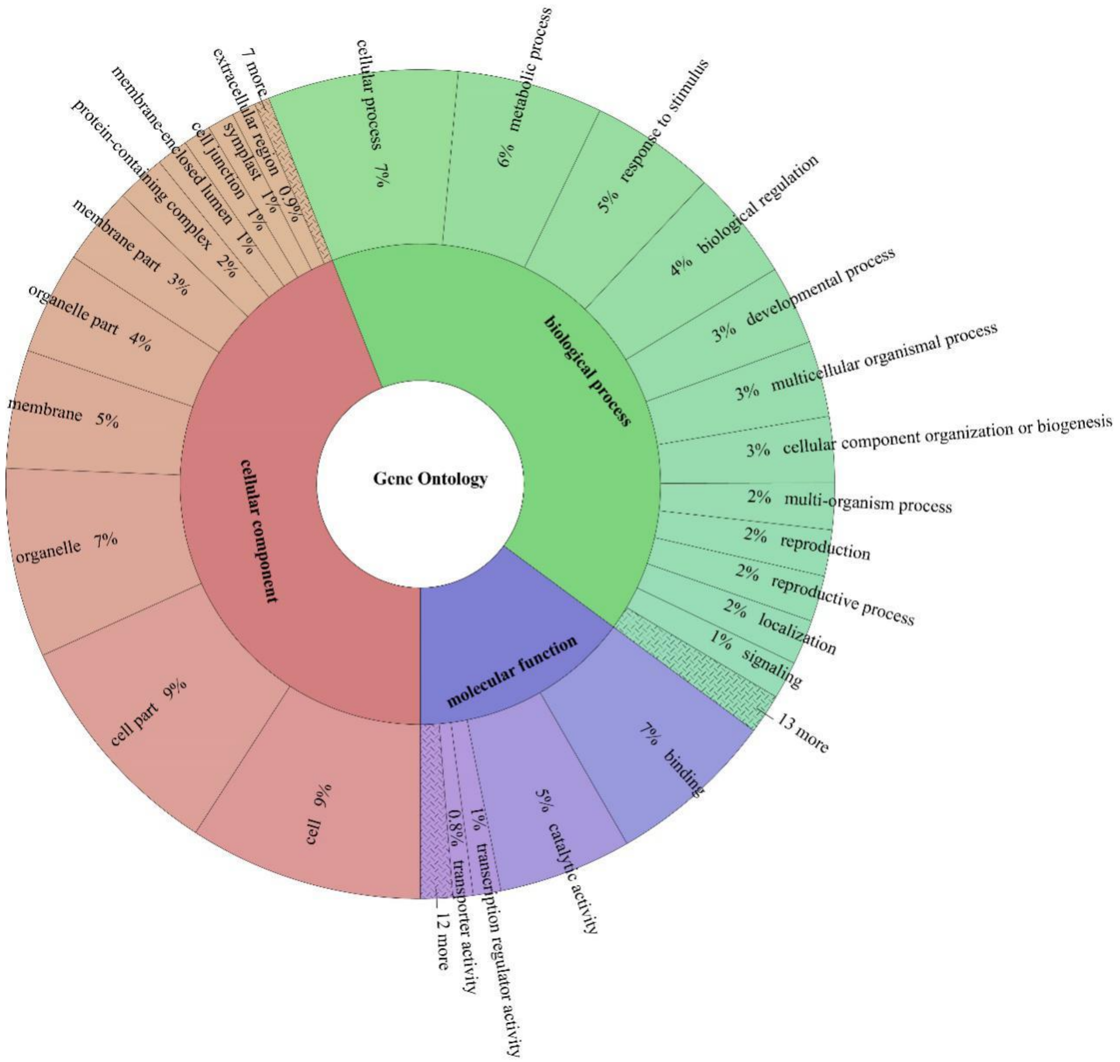

Supplementary Figure 6. Distribution of predicted genes of the cushion willow genome among different Gene Ontology terms.

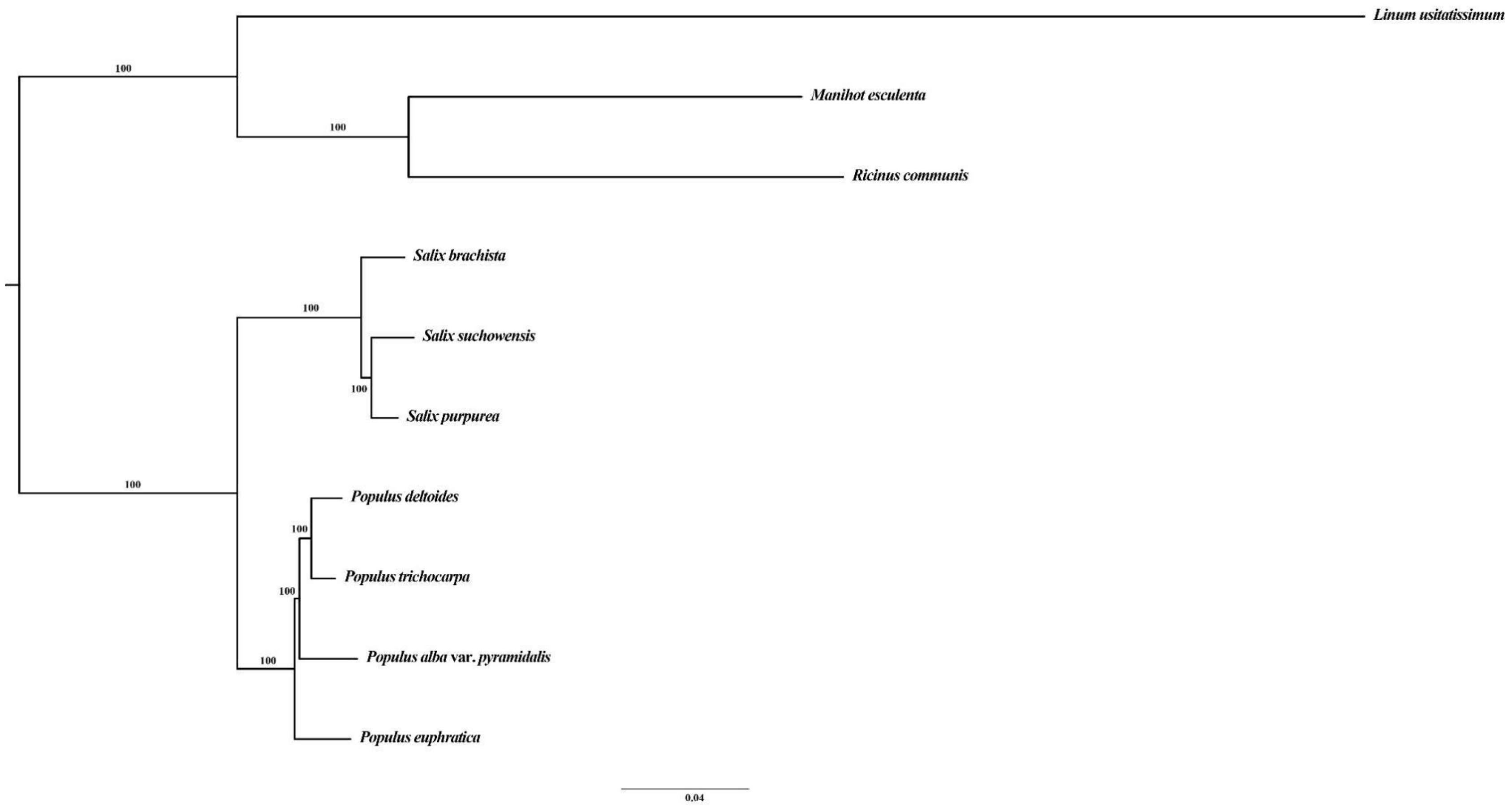

Supplementary Figure 7. Maximum likelihood phylogenetic tree constructed by RaxML with GTRGAMMA model based on 518 single copy genes of ten Malpighiales species. Clade supports were reported as bootstrap support value near the branch.

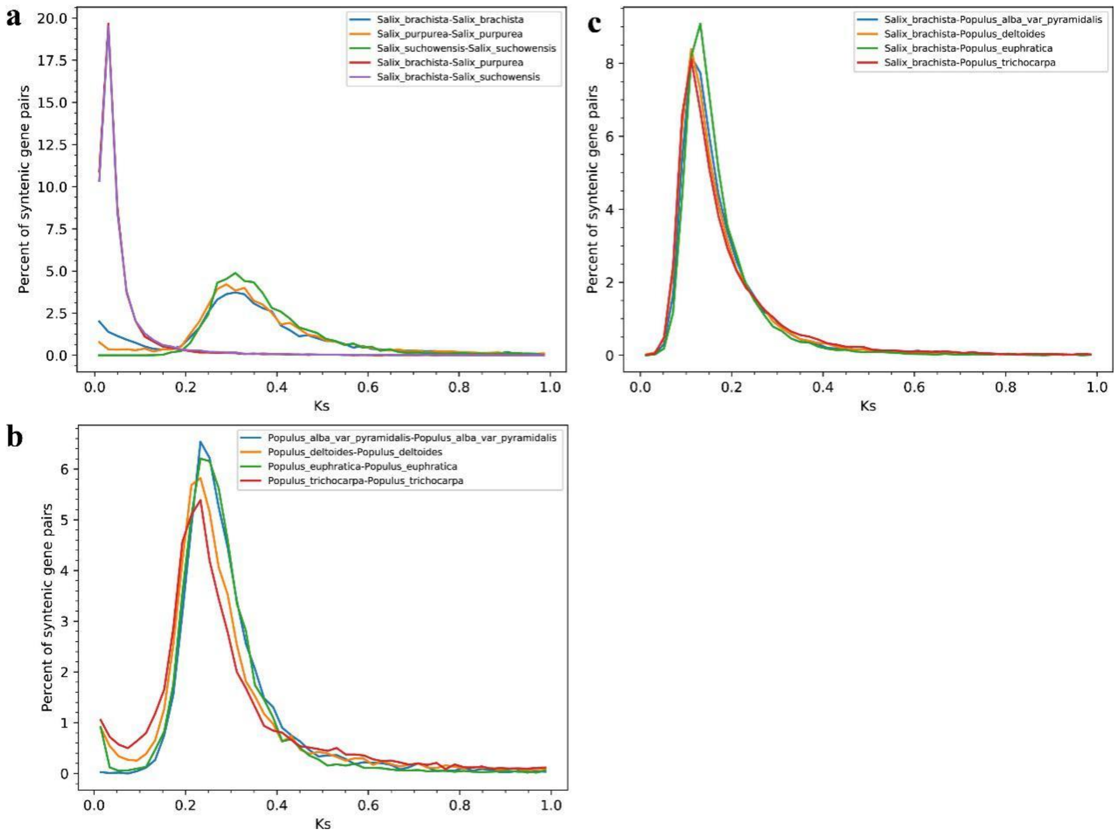

Supplementary Figure 8. *K*s values distributions. (**a**) three *Salix* species paris; (**b**) four *Populus* species; (**c**) between *Populus* species and *Salix brachista. K*s value around 0.3 is the common WGD shared by all *Salix* species as well as *Populus* species, which is around the *K*s value of 0.25. The peaks of divergence of lineage *Populus* and *Salix* is around the *K*s value of 0.13.

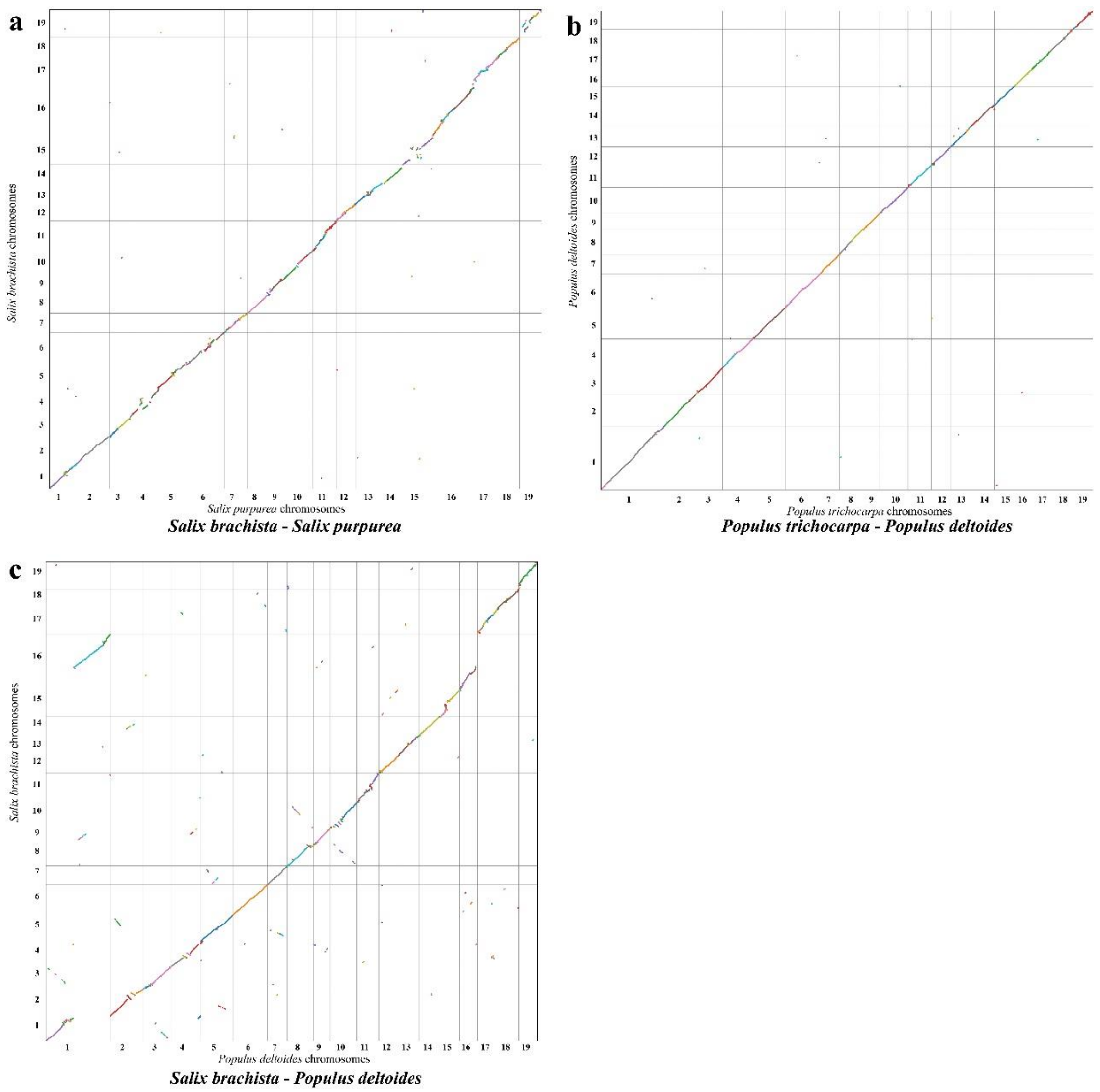

Supplementary Figure 9. Gene collinearity. (**a**) Between *S. brachista* and *S. suchowensis;* (**b**) Between *P. trichocarpa* and *P. deltoides;* (**c**) Between *S. brachista* and *P. deltoides*. The x-axis and y-axis corresponds to chromosomes.

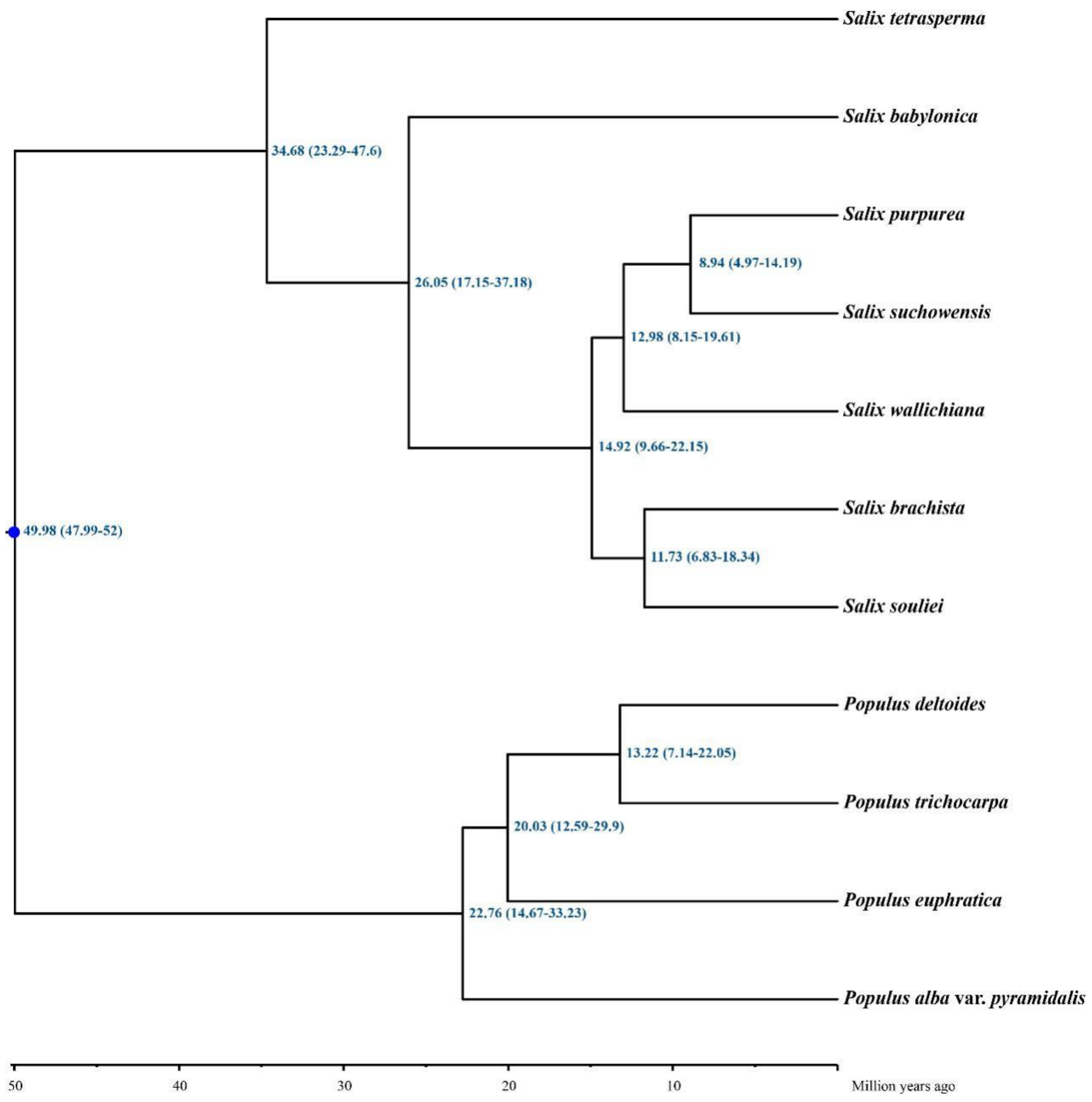

Supplementary Figure 10. Divergence time estimation. The phylogenetic tree was constructed by 390 single-copy genes shared by six Salicaceae species. The numbers below the branches refer to the divergence time (Ma) and its 95% HPD.

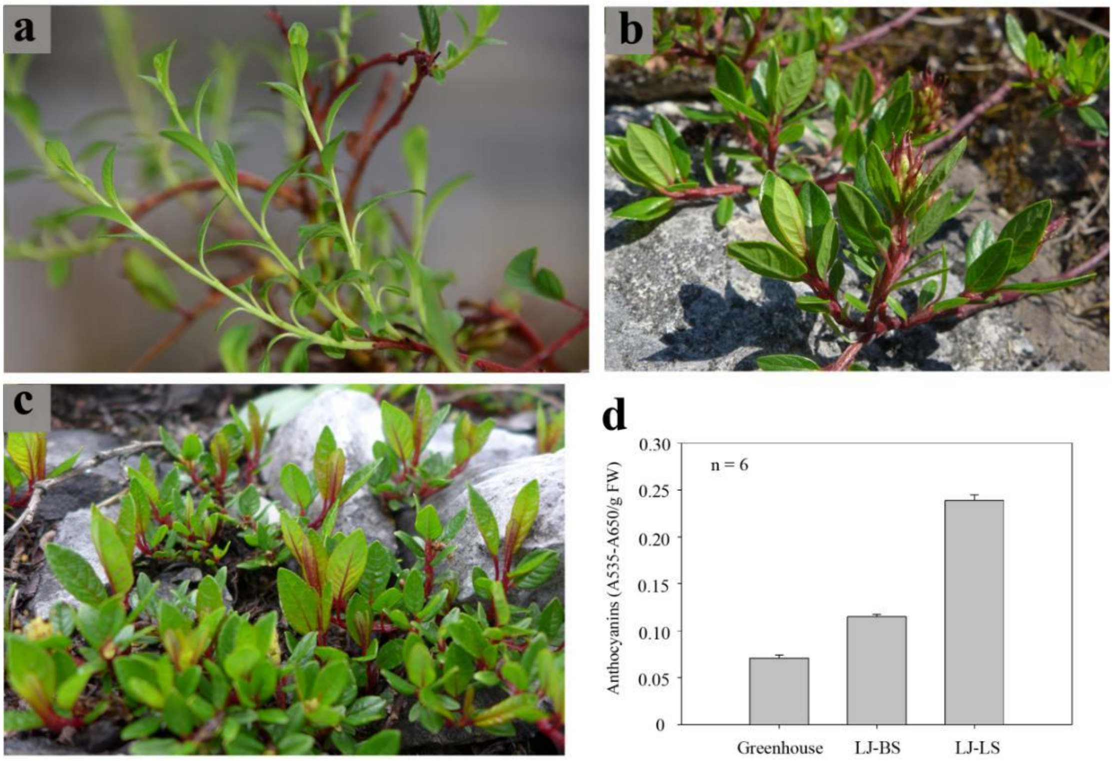

Supplementary Figure 11. Cushion willow individuals in difference localities which differs in anthocyanins concentration. (a) An individual maintained in greenhouse; (b) An individual of population of LJ_BS with elevation of 2950 m; (c) An individual of population of LJ_LS with elevation of 3950 m; (d) Branchlet anthocyanins concentration of the above individuals, error bars indicate standard deviation (SEM), i.e., mean + SEM. of anthocyanin concentration of six independent experiments. The source data underlying Supplementary Figure 11d are provided as a Source Data file.

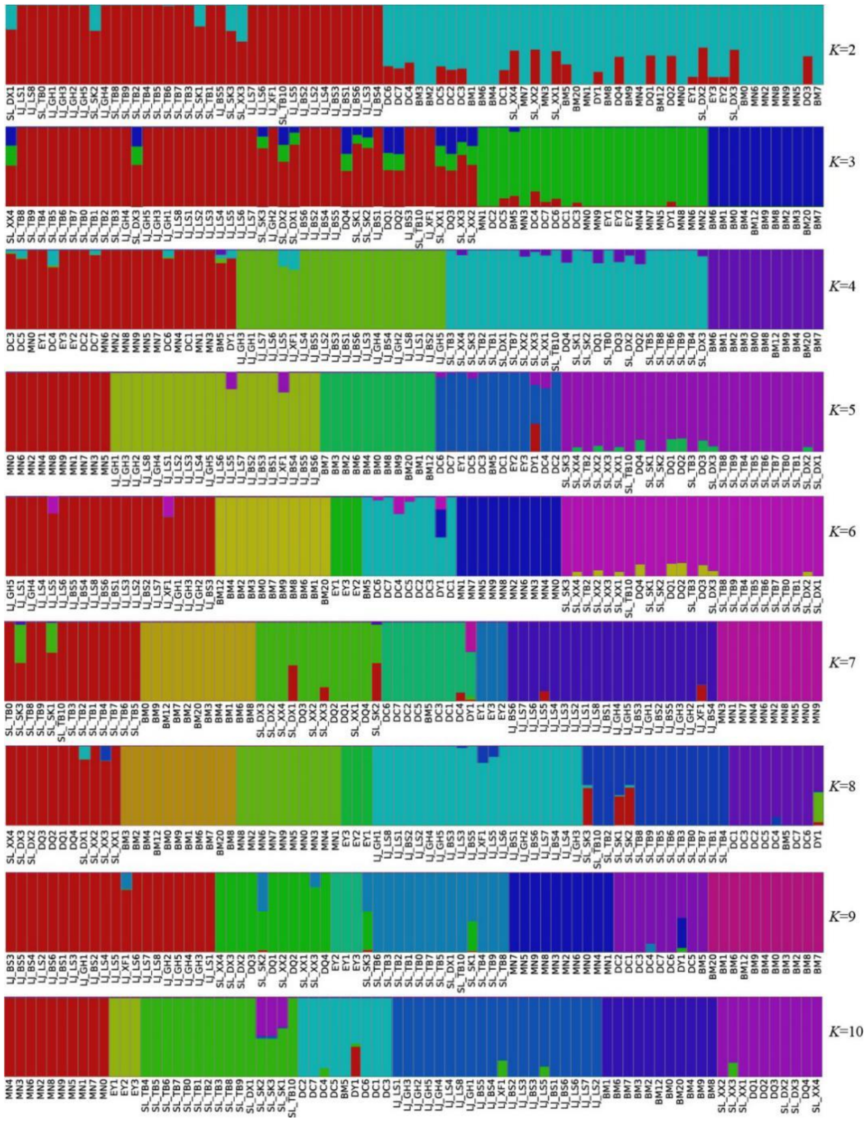

Supplementary Figure 12. Genetic structure analysis for 78 sequenced individuals using fastSTRUCTURE with *K* = 2 to 10. Each individual is represented by a stacked column, which is partitioned into 2 to 10 colored segments with the length of each segment representing the proportion of the individual's genome from *K* = 2 to 10 ancestral populations. The samples are sorted by population only after the analysis. The first level of clustering (*K* = 2) primary isolates populations of the distribution center (Shangri-La (SL) and Lijinag (LJ) county) of cushion willow from other peripheral population. At *K* = 3, the most west population in Bomi county (BM) become separated from the peripheral populations. At *K* = 4 the east Mianning county population (EY) was separated. At *K* = 5, the Lijiang county populations were separated from the Shangri-La county populations. At *K* = 6, the Eryuan county population was separated. At *K* = 7, the Tianbao mountain population (SL_TB) was separated from other Shangri-La county populations.

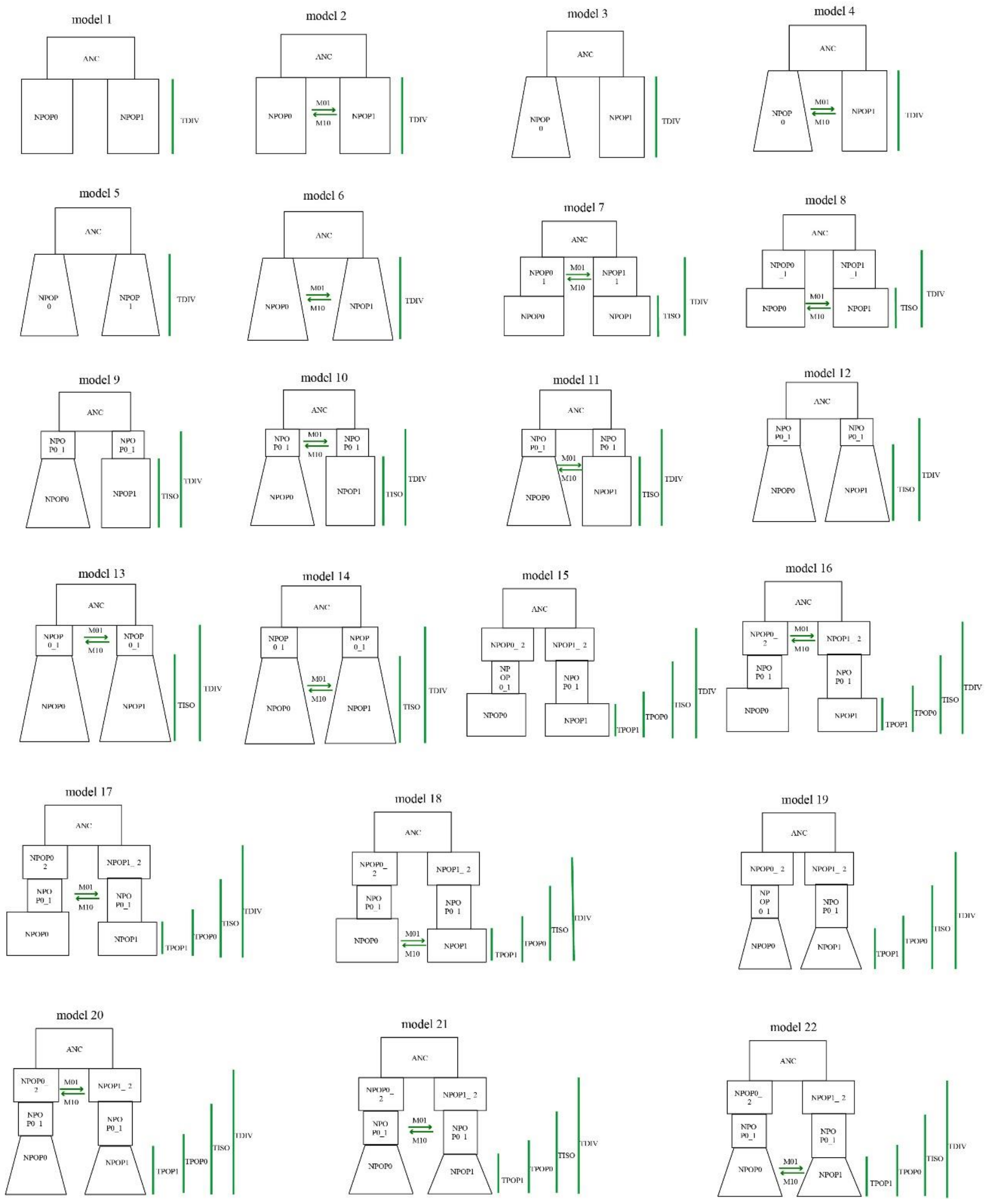

Supplementary Figure 13. Tested demographic models. Model1, isolation of two populations without gene flow; model2, isolation of two populations with asymmetric gene flow; model3, isolation of two populations with exponential population size change in one *population* and stepwise population size change in another population, no gene flow; model4, isolation of two populations with exponential population size change in one *population* and stepwise population size change in another population, with asymmetric gene flow; model5, isolation of two populations with exponential

population size changes in both populations, no gene flow; model6, isolation of two populations with exponential population size changes in both populations, with asymmetric gene flow; model7, isolation of two populations with stepwise population size changes in both populations, asymmetric gene flow in the early stage of population divergence until the time of TISO, no gene flow afterwards; model8, isolation of two populations with stepwise population size changes in both populations, no gene flow in the early stage of population divergence until the time of TISO, asymmetric gene flow afterwards; model9-model11, isolation of two populations with two steps of population size changes in both populations, both species experienced stepwise population size changes until the time of TISO, afterwards, *one* population experiencedexponential population size change, and another population experienced another stepwise change, the difference between models is the occurrence and the time of gene flow between populations; model12-model14, isolation of two populations with two steps of population size changes in both populations, both populations experienced stepwise population size changes until the time of TISO, afterwards, both populations experienced exponential population size changes, the difference between models is the occurrence and the time of gene flow between populations; model15-model18, isolation of two populations with three steps of stepwise population size changes in both populations, the difference between models is the occurrence and the time of gene flow between populations; model19-model22, same as model15-model18 but both populations experience exponential population size changes at the last step.

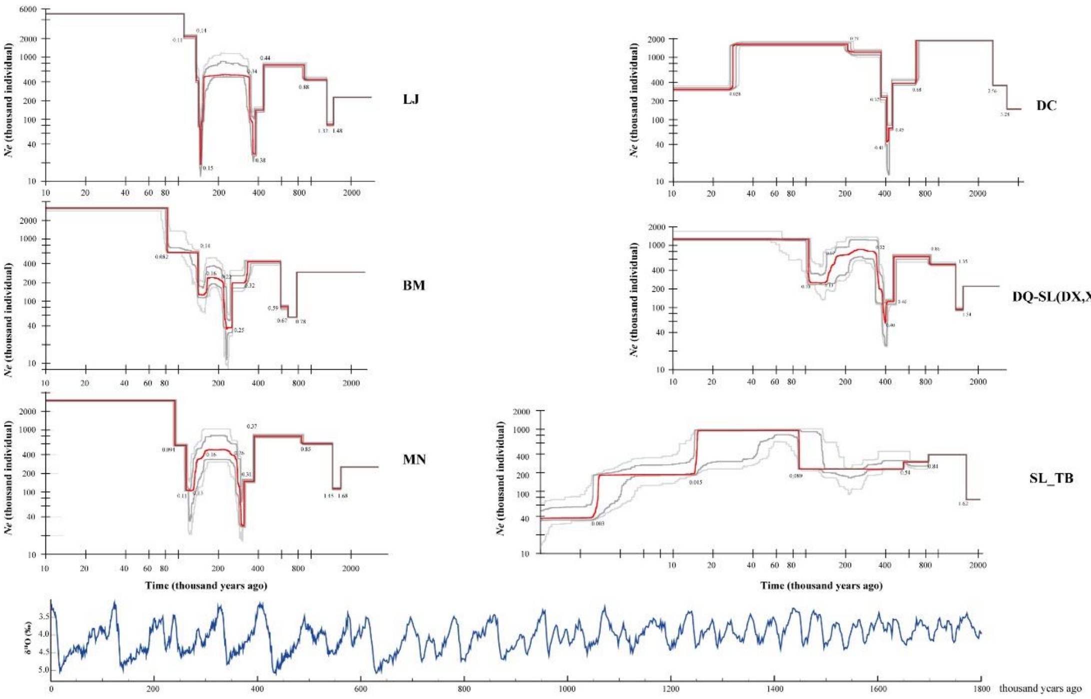

Supplementary Figure 14. Historical effective population size estimation. Based on six genetic group identified by structure analysis in Cushion willow, using generation time of 3 years. Numbers along the curves indicate the estimated times in million years. The $\delta^{18}O$ curve, based on composite stable oxygen isotope ratios from benthic foraminifera, and is an indicator of global ice volume and temperature[1].

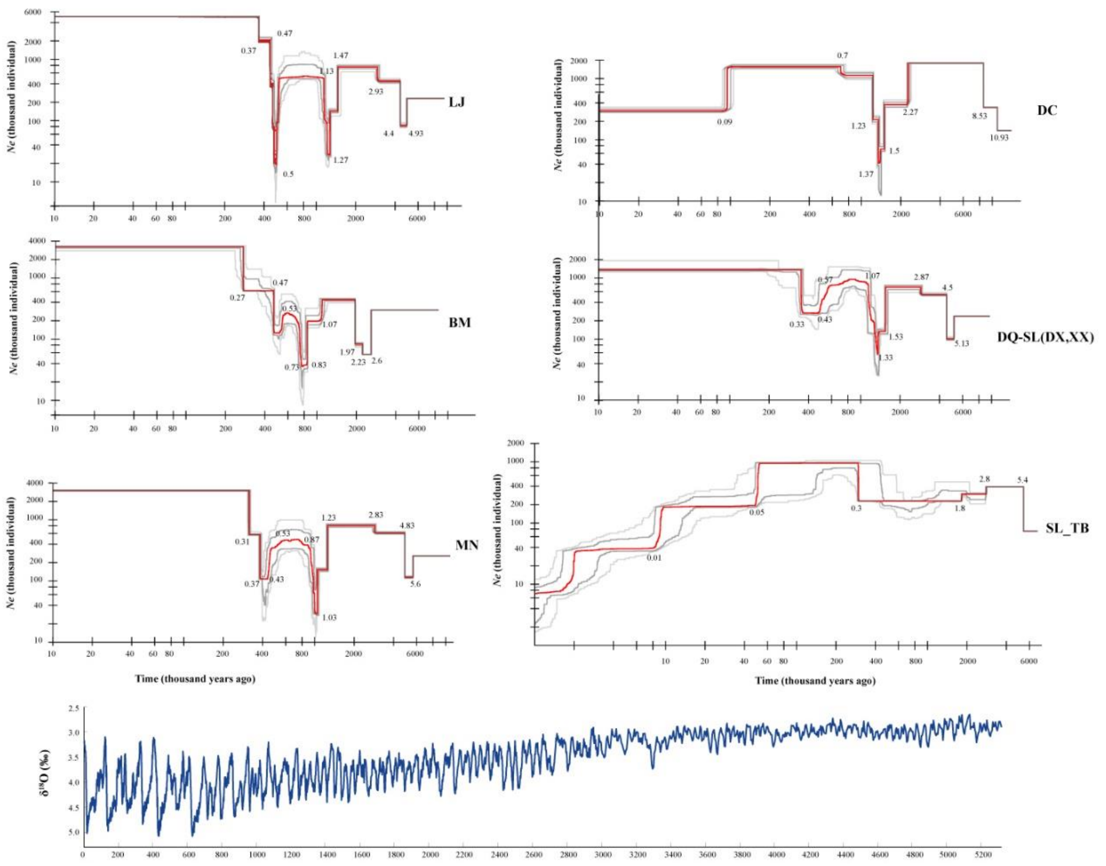

Supplementary Figure 15. Historical effective population size estimation. Based on six genetic group identified by structure analysis in Cushion willow, using generation time of 10 years. Numbers along the curves indicate the estimated times in million years. The $\delta^{18}O$ curve, based on composite stable oxygen isotope ratios from benthic foraminifera, and is an indicator of global ice volume and temperature[1].

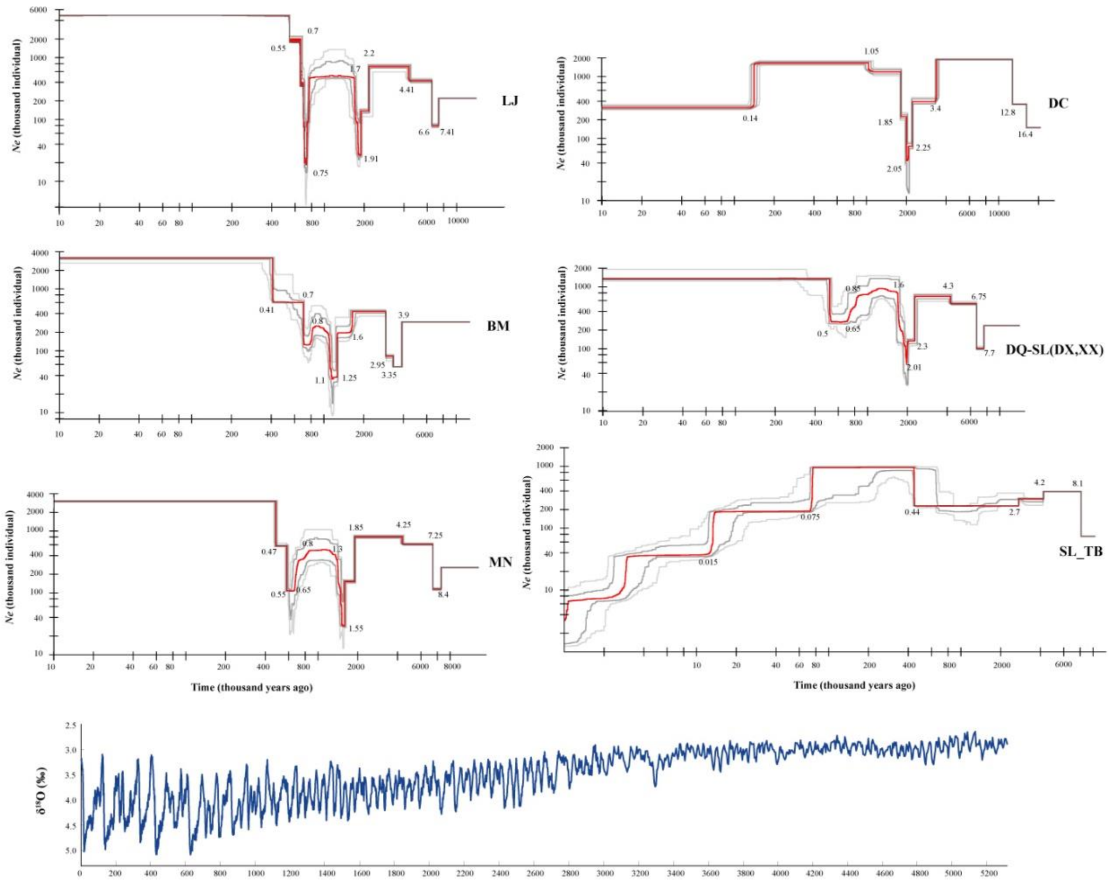

Supplementary Figure 16. Historical effective population size estimation. Based on six genetic group identified by structure analysis in Cushion willow, using generation time of 15 years. Numbers along the curves indicate the estimated times in million years. The δ^{18}O curve, based on composite stable oxygen isotope ratios from benthic foraminifera, and is an indicator of global ice volume and temperature[1].

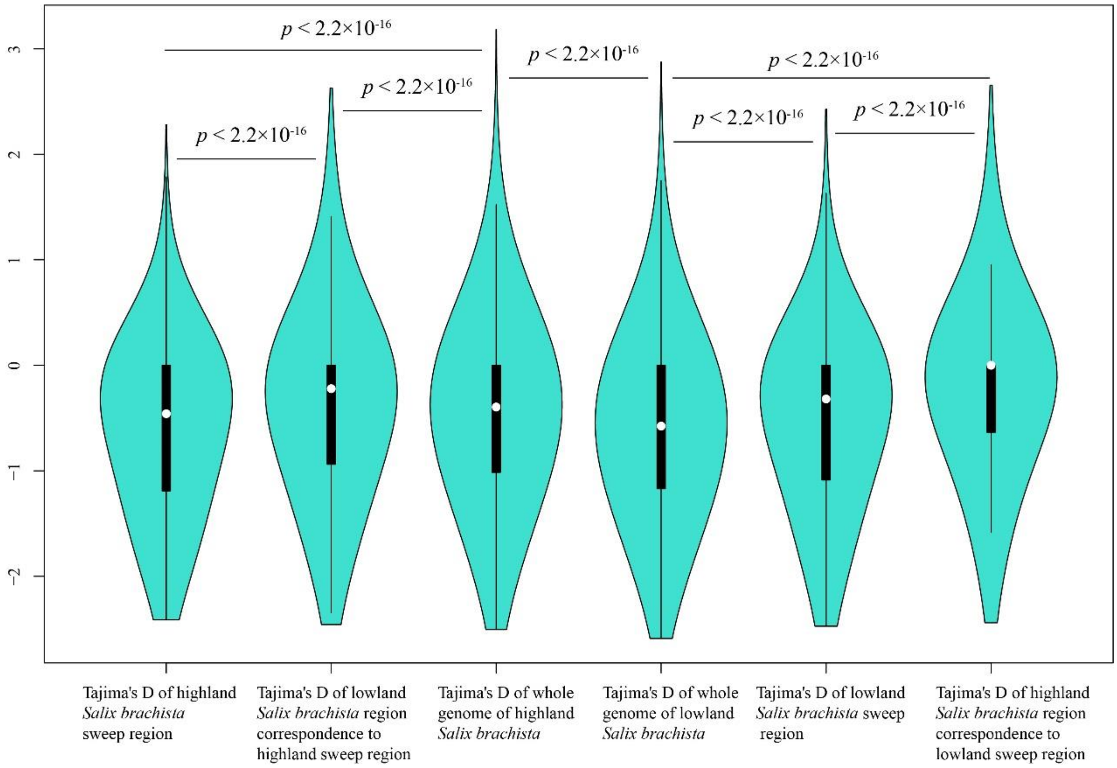

Supplementary Figure 17. Violin plot of Tajimas' *D* in highland and lowland LJ poulations of *Salix brachista* selective sweep region. Each "violin"with the width depicting a 90^0-rotated kernel density trace and its reflection. Vertical black boxes denote the interquartile range (IQR) between the first and third quartiles (25th and 75th percentiles, respectively) and the white point inside denotes the median. (a) Tajimas' *D* values of highland, whole genome and lowland in highland S. brachista selective sweep region defined by CLR; (b) Tajimas' *D* values of highland, whole genome and lowland in lowland S. brachista selective sweep region defined by CLR. The statistical significance was calculated by the Mann-Whitney *U* test, n = 4,237, 4,237 168,543, 168,543, 7,088 7,088 for the six plots from left to right, respectively. Source data are provided as a Source Data file.

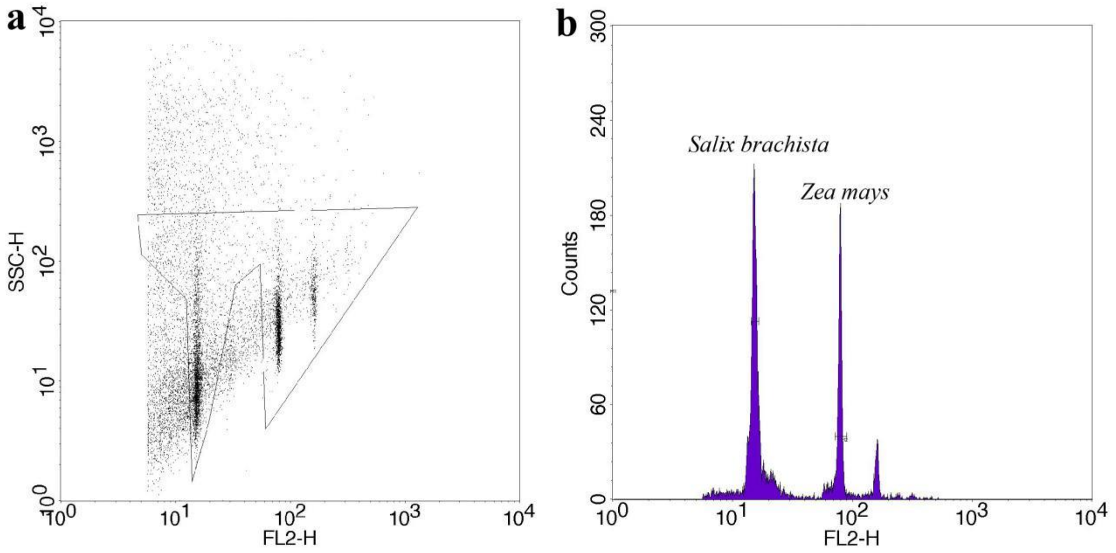

Supplementary Figure 18. Flow cytometry estimation of the genome size for the whole-genome sequenced *Salix brachista* individual. (a) The gating made in the dot-plot of side scatter (SSC) versus fluorescence intensity channel 2 (FL2) to exclude as much as possible partial nuclei and other types of debris; (b) Flow cytometric histograms of relative fluorescence intensities of propidium iodide-stained nuclei simultaneously isolated from *Salix brachista* and the plant DNA reference standard, *Zea mays* (maize).

Supplementary Table 1. Flow cytometry results of the *Salix brachista* indivudual sequenced.

Replicates	Estimated genome size (MB)	Material
Replicate 1	422.37	Leaves of tissue culture seedlings
Replicate 2	410.42	Leaves of tissue culture seedlings
Replicate 3	412.74	Leaves of tissue culture seedlings
Replicate 4	410.52	Leaves of tissue culture seedlings
Replicate 5	420.05	Leaves of tissue culture seedlings
Replicate 6	415.33	Leaves of tissue culture seedlings
Replicate 7	424.07	Leaves of tissue culture seedlings
Replicate 8	428.82	Leaves of tissue culture seedlings
Replicate 9	434.62	Leaves of tissue culture seedlings
Replicate 10	405.96	Leaves of tissue culture seedlings
Replicate 11	432.57	Leaves of tissue culture seedlings
Replicate 12	419.23	Leaves of wild plant
Replicate 13	415.84	Leaves of wild plant
Replicate 14	422.81	Leaves of wild plant
Replicate 15	421.83	Leaves of wild plant
Replicate 16	426.70	Leaves of wild plant
Replicate 17	430.24	Leaves of wild plant
**Mean estimated genome size (MB)**		**420.83**
**Standard deviation (MB)**		**8.26**

Source data are provided as a Source Data file.

Supplementary Table 2. **Statistics of the ONT datasets.**

	Raw data	Read length more than 35 kb
**Base number**	29,685,918,796 bp	17,391,540,559 bp
**Read number**	3,051,540	341,325 bp
**Maximum length**	426,676 bp	274,665 bp
**Minimum length**	5 bp	35,001 bp
**Mean length**	9,728 bp	50,953 bp
**Median length**	3856 bp	
**N50**	25,814 bp	50,110 bp
**Number of reads > 1Kb**	2,256,686	
**Number of reads > 5Kb**	1,439,030	
**Number of reads > 10Kb**	963,258	
**Number of reads > 20Kb**	503,908	
**Number of reads > 50Kb**	81,954	
**N10**	61,372 bp	
**L10**	38287	
**L50**	349507	
**N90**	6,285 bp	
**L90**	1,229,220	

Supplementary Table 3. DNA-seq and RNA-seq read information of *Salix brachista*.

Data	Purpose	Tissue	Platform	Library insert size	Read length (average)	Clean reads (million)	Clean Bases(G)	Base coverage
Hi-C DNA-seq (scaffolding)	Genome assembly scaffolding	Leaves and branches of tissue culture seedlings	Illumina Hiseq X Ten	300-500 bp	149.7 bp	230.929	34.573	~86 ×
WGS DNA-seq (PCR-free sequencing)	Assist Whole-genome assembly	Leaves and branches of tissue culture seedlings	Illumina Hiseq X Ten	300 bp	147.2 bp	118.97	17.514	~43×
WGS DNA-seq (genome assembly)	Assist Whole-genome assembly	Leaves and branches of tissue culture seedlings	PacBio RSII	20 kb	7300 bp	6.863	50.097	~125 ×
WGS DNA-seq (genome assembly)	Whole-genome assembly	Leaves and branches of tissue culture seedlings	Oxford Nanopore Technologies PromethION	/	25,814 bp (N50)	0.341	29.69	~74 ×
RNA-seq	Assit annotation	Whole plant of tissue culture seedlings	Illumina Hiseq X Ten	300-500 bp	140 bp	230.929	32.33	/

Supplementary Table 4. Assembly Statistics by initial assembly and postprocessing.

	Canu correction+SMARTdenovo	Polishing by PacBio (2 rounds)	Polishing by Illumina (5 rounds)	Hi-C scaffolding	Gap closure	Redundency and contamination remover	Final assembly (polishing by Illumina for 2 rounds)
**Assembled genome size**	343,569,608 bp	351,674,479 bp	351,661,322 bp	352,440,809 bp	352,441,780 bp	339,587,529 bp	339,587,529 bp
**Assembled contig number**	245	245	245	259	158	78	78
**Contig N50 (L50)**	5,216,507 bp (21)	5,341,994 bp (21)	5,340,722 bp (21)	5,340,722 bp (21)	9,522,530 bp (13)	9,522,514bp (13)	9,522,514bp (13)
**Congtig N90 (L90)**	742,640 bp (95)	759,813 bp (95)	706,485 (96)	660,606 bp (98)	2,120,086 bp (42)	2,775,137 bp (38)	2,775,137 bp (38)
**Maximum contig length**	13,769,798 bp	14,111,614 bp	14,108,333 bp	14,108,333 bp	21,274,067 bp	21,274,067 bp	21,274,067 bp
**Scaffold number**	/	/	/	110	110	30	30
**Scaffold N50 (L50)**	/	/	/	17,921,935 bp (8)	17,922,049 bp (8)	17,922,059 bp (8)	17,922,059 bp (8)
**Scaffold N90 (L90)**	/	/	/	11,704,698 bp (18)	11,704,698 bp (18)	13,388,179 bp (17)	13,388,179 bp (17)
**Maximum scaffold length**	/	/	/	39,689,041 bp	39,689,241bp	39,688,537 bp	39,688,537 bp
**No. of N**	0	0	0	14900	3434	3434	3434
**BUSCO**	59.80%	95.60%	96.00%	96.00%	96.00%	96.00%	96.10%
**Genome-wide quality***	99% (Q20)	99.95% (Q33)	99.9943% (Q42)	99.9943% (Q42)	99.9943% (Q42)	99.9943% (Q42)	99.995% (Q43)
**Gap number**				149	48	48	48
**Heterozygosity****	0.58%	0.66%	0.65%	0.65%	0.65%	0.65%	0.71%

*Genome-wide quality was estimated by number of SNPs called by Samtools and Bcftools that inconsistent with the genome assembly according to by Michael *et al.*[2].

**Heterozygosity was estimated by het-total (total heterozygosity sites)/assemblyed length.

Supplementary Table 5. **Length statistics of the final reference genome of *Salix brachista*.**

Pseudo-chromosomes (chromosome-scale scaffolds)	Length (bp)
chr01	17,456,518
chr02	18,886,152
chr03	16,097,183
chr04	16,415,100
chr05	19,994,850
chr06	21,273,787
chr07	13,388,179
chr08	14,644,405
chr09	11,696,614
chr10	17,922,059
chr11	20,884,143
chr12	11,704,711
chr13	14,579,692
chr14	13,581,586
chr15	18,554,468
chr16	39,688,537
chr17	17,101,791
chr18	14,058,352
chr19	19,345,208
**Organelles**	**Length (bp)**
Mitochondrial	608,983
Chloroplast	155,604
**Contigs**	**Length (bp)**
ctg41	109,304
ctg46	64,030
ctg51	434,949
ctg55	57,080
ctg68	102,464
ctg72	317,584
ctg76	232,646
ctg82	94,927
ctg108	136,623

Supplementary Table 6. Mapping statistics of Illummina PCR-free short read, PacBio and ONT long reads on the *Salix brachista* genome assembly.

	**Illumina**	**PacBio**	**ONT (all reads)**	**ONT (reads length > 10 kb)**	**ONT (reads length > 20 kb)**	**ONT (reads length > 50 kb)**
**Total reads**	118,971,528	7,300,146	4,046,360	963,258	503,908	81,954
**Reads mapped (percent)**	117,170,055 (98.49%)	6,425,006 (88.01%)	2,056,720 (50.82%)	891,250 (92.52%)	475,504 (94.36%)	79,758 (97.32%)
**Reads mapped and paired (percent)**	116,975,292 (98.32%)	/	/	/	/	/
**Total bases**	17,513,893,116	51,645,304,243	31,030,589,089	25,059,007,736	18,484,049,642	5,378,202,363
**Bases mapped (percent)**	17,247,730,588 (98.48%)	48,549,383,351 (94.01%)	28,341,248,503 (91.33%)	23,536,462,850 (93.92%)	17,574,980,300 (95.08%)	5,238,427,825 (97.40%)
**Pairs on different chromosomes**	990321 (1.66%)	0	0	/	/	/
**Genome assembly mapped bases with at least 10× (percent)**	335,601,106 (98.83%)	338,117,569 (99.57%)	338,425,071 (99.66%)	/	/	/
**Average depth**	37.1	75.9	59	/	/	/

Supplementary Table 7. **Classification of repetitive DNA in the genomes of six Salicaceae species.**

**Repeat elements**		***Salix brachista***		***Salix purpurea***		***Salix suchowensis***		***Populus trichocarpa***		***Populus euphratica***		***Populus alba* var. *pyramidalis***	
**Order**	**Superfamily**	length(bp)	percent(%)	length(bp)	percent(%)	length(bp)	percent(%)	length(bp)	percent(%)	length(bp)	percent(%)	length(bp)	percent(%)
**LTR**		63693559	18.76	48941710	10.29	43716562	14.39	85457426	19.68	135303555	27.28	84917956	18.28
	**Copia**	31068388	9.15	21324367	4.49	19378042	6.38	19381828	4.46	18420281	3.71	22812755	4.91
	**Gypsy**	32060166	9.44	27181033	5.72	23694577	7.80	64946615	14.96	115880136	23.36	61571369	13.25
**LINE**		2519123	0.74	3457315	0.73	2744685	0.90	4452603	1.03	2862100	0.58	5113574	1.10
**SINE**		4609088	1.36	3834045	0.81	2928596	0.96	2628482	0.61	1373979	0.28	1976196	0.43
**DNA**		16303643	4.80	10493322	2.21	10090550	3.32	29843825	6.87	22006242	4.44	23213605	5.00
**RC**		17124852	5.04	20432059	4.30	17494484	5.76	32030714	7.38	22805557	4.60	21176430	4.56
**Unknown**		27658576	8.14	27262985	5.73	31063511	10.23	26381221	6.08	26211600	5.28	48106918	10.36
**rRNA**		151917	0.04	41584	0.01	86070	0.03	190109	0.04	804464	0.16	1656992	0.36
**Satellite**		24047	0.01					2886975	0.66	141683	0.03	428045	0.09
**Simple repeat**		8402707	2.47	9413723	1.98	4911254	1.62	5272722	1.21	6286613	1.27	5395848	1.16
**Low complexity**		959019	0.28	1128609	0.24	868069	0.29	1222533	0.28	1550246	0.31	1500089	0.32
**Total**		141446531	41.65	125005352	26.29	113919728	37.50	190366610	43.85	219346039	44.22	193485653	41.65

Supplementary Table 8. **RNAseq assembly and mapping using the *Salix brachista* genome assembly.**

**Assembly method**	**No. of transcripts assembled**	**No. of transcripts mapped to *Salix brachista* genome assembly**	**Percent of transcripts mapped to *Salix brachista* genome assembly (%)**
Trinity denovo	89,391	88,687	99.21
hisat2+stringtie*	70,585	70,527	99.92
hisat2+Trinity genome-guided*	169,942	169,809	99.92
hisat2+cufflinks	52,182	52,149	99.94
tophat2(refgene)+cufflinks	53,332	53,292	99.92
tophat2+cufflinks	49,538	49,498	99.92

*These two RNA-Seq assemblies were used in genome annotation, and other RNA-Seq assemblies were used for genome assembly assessment.

Supplementary Table 9. **Functional annotation of the predicted *Salix brachista* genes.**

	**Number**	**Percent**	**Databasea website**
Total gene number	30209	100.00%	
Swiss_Prot	19830	65.60%	http://www.ebi.ac.uk/swissprot/
TrEMBL	29159	96.50%	https://www.ebi.ac.uk/uniprot
NR	29254	96.80%	https://www.ncbi.nlm.nih.gov/protein
Pfam	25063	83.00%	https://pfam.xfam.org/
eggNOG	28342	93.80%	http://eggnogdb.embl.de/
GO	19600	64.90%	http://geneontology.org/
KEGG	2076	6.87%	https://www.genome.jp/kegg/pathway.html
KO	11957	39.60%	https://www.genome.jp/kegg/ko.html
PANTHER	27887	92.31%	http://pantherdb.org/
**Unannotated**	**785**	**2.60%**	
**Total annotated**	**29424**	**97.40%**	

Supplementary Table 10. **KEGG enrichment of *Salix brachista* expanded gene families using *Salix brachista* genome as background.**

KEGG terms	KEGG ID	No of input genes	No of backgrond genes	*p* value	Adjust *p* value
Base excision repair	ko03410	27	66	9.11E-11	1.00E-08
Flavonoid biosynthesis	ko00941	29	94	3.92E-08	2.16E-06
Indole alkaloid biosynthesis	ko00901	19	47	7.09E-08	2.60E-06
Ribosome biogenesis in eukaryotes	ko03008	30	109	3.83E-07	1.05E-05
Phenylpropanoid biosynthesis	ko00940	47	227	2.57E-06	5.66E-05
Stilbenoid, diarylheptanoid and gingerol biosynthesis	ko00945	16	47	1.08E-05	0.00019797
Tyrosine metabolism	ko00350	23	86	1.44E-05	0.00022595
Phenylalanine metabolism	ko00360	21	88	0.000199225	0.002739339
Amino sugar and nucleotide sugar metabolism	ko00520	37	199	0.000301878	0.00368962
Protein processing in endoplasmic reticulum	ko04141	53	324	0.000511592	0.005627517
Cutin, suberine and wax biosynthesis	ko00073	17	71	0.00075251	0.007525096

P-values were calculated by Fisher's exact test.

Supplementary Table 11. **Summary of SNP calling on a population-scale.**

Variation Type	Total variations result from SNP calling	No. of SNP after filtering	High quality SNPs (MAF ≥ 0.05)
**Indels**	3,013,348	0	0
**SNPs**	21,254,233	6,524,760	1,595,067
**MNPs (multi-nucleotide polymorphisms)**	3,128,743	0	0
**Unknown**	1,134,529	0	0
**Multiallelic sites**	5,070,414	0	0
**Multiallelic SNP sites**	912,769	0	0
**Total**	**23,939,702**	**6,524,760**	**1,595,067**

Supplementary Table 12. Sample information and RNA assembly results by Trinity denovo of four *Salix* species used for divergence time estimation.

Organism	Sample information (Locality; elevation; cordinate)	Voucher and herbarium	Assembly method	No. of transcripts assembled	No. of CDS assembled	NCBI accession for raw RNA-Seqdata
Salix babylonica Linnaeus	Kunming,Yunnan,China;1930m; N25.138081, E102.745942	CJH7007, KUN*	Trinity denovo	64125	32390	PRJNA545026
Salix wallichiana Andersson	Deqin,Yunnan,China;3520m; N28.302237, E99.140324	WDC13002, KUN	Trinity denovo	93012	46432	
Salix souliei Seemen	Lijiang,Yunnan,China;4110m; N27.059558, E100.195036	WDC13004, KUN	Trinity denovo	80020	39349	
Salix tetrasperma (Roxburgh) N. Chao & G. T. Gong	Chengjiang,Yunnan,China;1720m; N24.605443, E102.841163	WDC13005, KUN	Trinity denovo	105314	47741	

*KUN: Herbarium of Kunming Institute of Botany.

Supplementary Table 13. Assembly statistics of different methods.

Assembly method	Assembled genome size	Assembled contig number	N50 length	L50 number	Maximum contig length
**wtdbg2**	336 Mb	3978	618 kb	136	4.5 Mb
**canu correction+wtdbg2**	360 Mb	2982	1.0 Mb	87	7.2 Mb
**canu correction+smartdenovo (k=20)**	344 Mb	245	5.2 Mb	21	13.8 Mb
**canu correctioin+smartdenovo (k=18-23)**	337-339 Mb	247-273	3.2-4.9 Mb	22-30	11.5-16.3 Mb

Supplementary Table 14. Sources of the genome assembly datasets used in this study.

Organism	Version	URL	Reference
Arabidopsis thaliana	TAIR10	https://phytozome.jgi.doe.gov/pz/portal.html#!info?alias=Org_Athaliana	Lamesch et al.[3]
Populus trichocarpa	v3.1	https://phytozome.jgi.doe.gov/pz/portal.html#!info?alias=Org_Ptrichocarpa_er	Tuskan et al.[4]
P. euphratica	v1.0	https://www.ncbi.nlm.nih.gov/genome/annotation_euk/Populus_euphratica/100/	Ma et al.[5]
P. deltoides	v2.1	https://phytozome.jgi.doe.gov/pz/portal.html#!info?alias=Org_PdeltoidesWV94_er	/
P. alba var. *pyramidalis*		http://bigd.big.ac.cn/gwh/	Ma et al.[6]
Salix suchowensis	v4.1	http://popgenie.org/	Dai *et al.*[7]
S. purpurea	v1.0	https://phytozome.jgi.doe.gov/pz/portal.html#!info?alias=Org_Spurpurea	Zhou et al.[8]
Linum usitatissimum	v1.0	https://phytozome.jgi.doe.gov/pz/portal.html#!info?alias=Org_Lusitatissimum	Wang et al.[9]
Manihot esculenta	v6.1	https://phytozome.jgi.doe.gov/pz/portal.html#!info?alias=Org_Mesculenta	Bredeson *et al.*[10]
Ricinus communis	v0.1	https://phytozome.jgi.doe.gov/pz/portal.html#!info?alias=Org_Rcommunis	Chan *et al.*[11]

Supplementary References

1. Lisiecki L. E., Raymo M. E. A Pliocene-Pleistocene stack of 57 globally distributed benthic delta O-18 records. *Paleoceanography* **20**, PA1003 (2005).

2. Michael T. P., et al. High contiguity *Arabidopsis thaliana* genome assembly with a single nanopore flow cell. *Nat. Commun.* **9**, 541 (2018).

3. Lamesch P., *et al.* The *Arabidopsis* Information Resource (TAIR): Improved gene annotation and new tools. *Nucleic Acids Res.* **40**, D1202-1210 (2012).

4. Tuskan G. A., *et al.* The genome of black cottonwood, *Populus trichocarpa* (Torr. & Gray). *Science* **313**, 1596-1604 (2006).

5. Ma T., *et al.* Genomic insights into salt adaptation in a desert poplar. *Nat. Commun.* **4**, 2797 (2013).

6. Ma J., *et al.* Genome sequence and genetic transformation of a widely distributed and cultivated poplar. *Plant Biotechnol. J.* **17**, 451-460 (2019).

7. Dai X. G., *et al.* The willow genome and divergent evolution from poplar after the common genome duplication. *Cell Res.* **24**, 1274-1277 (2014).

8. Zhou R., *et al.* Characterization of a large sex determination region in *Salix purpurea* L. (Salicaceae). *Mol. Genet. Genomics* **293**, 1437-1452 (2018).

9. Wang Z., *et al.* The genome of flax (*Linum usitatissimum*) assembled de novo from short shotgun sequence reads. *Plant J.* **72**, 461-473 (2012).

10. Bredeson J. V., *et al.* Sequencing wild and cultivated cassava and related species reveals extensive interspecific hybridization and genetic diversity. *Nat. Biotechnol.* **34**, 562-570 (2016).

11. Chan A. P., *et al.* Draft genome sequence of the oilseed species *Ricinus communis. Nat. Biotechnol.* **28**, 951-956 (2010).
