# Supplementary material for: Genome-wide analysis of Cushion willow provides insights into alpine plant divergence in a biodiversity hotspot
Source: Nat Commun. 2019 Nov 19;10:5230. doi: 10.1038/s41467-019-13128-y (PMC6864086; doi:10.1038/s41467-019-13128-y)
Supplement: Supplementary file 3 — Reporting summary [file 41467_2019_13128_MOESM3_ESM.pdf]

nature research

Corresponding author(s): Jiahui Chen

Last updated by author(s): Oct 9, 2019

Reporting Summary

Nature Research wishes to improve the reproducibility of the work that we publish. This form provides structure for consistency and transparency in reporting. For further information on Nature Research policies, see Authors & Referees and the Editorial Policy Checklist .

Statistics

For all statistical analyses, confirm that the following items are present in the figure legend, table legend, main text, or Methods section.

n/a	Confirmed	
[ ]	[x]	The exact sample size (*n*) for each experimental group/condition, given as a discrete number and unit of measurement
[ ]	[x]	A statement on whether measurements were taken from distinct samples or whether the same sample was measured repeatedly
[ ]	[x]	The statistical test(s) used AND whether they are one- or two-sided<br>*Only common tests should be described solely by name; describe more complex techniques in the Methods section.*
[x]	[ ]	A description of all covariates tested
[ ]	[x]	A description of any assumptions or corrections, such as tests of normality and adjustment for multiple comparisons
[ ]	[x]	A full description of the statistical parameters including central tendency (e.g. means) or other basic estimates (e.g. regression coefficient) AND variation (e.g. standard deviation) or associated estimates of uncertainty (e.g. confidence intervals)
[ ]	[x]	For null hypothesis testing, the test statistic (e.g. *F*, *t*, *r*) with confidence intervals, effect sizes, degrees of freedom and *P* value noted<br>*Give P values as exact values whenever suitable.*
[ ]	[x]	For Bayesian analysis, information on the choice of priors and Markov chain Monte Carlo settings
[x]	[ ]	For hierarchical and complex designs, identification of the appropriate level for tests and full reporting of outcomes
[ ]	[x]	Estimates of effect sizes (e.g. Cohen's *d*, Pearson's *r*), indicating how they were calculated

Our web collection on statistics for biologists contains articles on many of the points above.

Software and code

Policy information about availability of computer code

Data collection	Besides the data generated in this study, other genome data were directly download from online database such as GenBank and Phytozome.

Data analysis	SMARTdenovo v1.0 and wtdbg2 assemblers with ONT reads corrected by Canu was used for genome assembly. PacBio long reads was mapped to the assembled genome using pbmm2 and polished by arrow, Illumina short reads was mapped to the assembled genome using BWA-MEM and polished by pilon. Hi-C reads were mapped to the assembly with Juicer, and then a candidate chromosome-length assembly was generated automatically using the 3d-DNA pipeline to correct mis-joins, order, orient and anchor contigs from the assembly. Manual review and refinement of the candidate assembly was performed in Juicebox Assembly Tools (JBAT) for quality control and interactive correction. To reduce the influence of interactions of chromosomes and to further improve the chromosome-scale assembly, each chromosome was re-scaffolded with 3d-DNA separately, and then manually refined with Juicebox. With the modified 3d-DNA and JBAT workflow, 19 chromosomes (337.28 Mb, 99.32%) were anchored. LR_Gapcloser was used for gap closure. Repeat families found in the genome assemblies o f Cushion willow were independently identified de novo and classified initially using the software package RepeatModeler. The Augustus ab initio gene finder was used to identify gene models for Cushion willow genome assemblies. Maker pipeline was used for gene anotation. Orthofinder2 was used for orthologous identification. RNA-seq reads were preprocessed by Cutadapt to remove contaminating sequences from adaptors and low base quality sequences. And then mapped the RNA-seq read to genome assembly by HiSat2, and used StringTie for referencing-guided assembly and Trinity for genome-guild de novo assembly. Transcript redundants were removed, using CD-HIT. MCScanX was then use d for collinear analysis. CAFE was used for gene family analysis. RaxML 7.0.3 and Mega 7 were used for phylogenetic tree constructing. MCMCtree implemented in PAML 4.0 package was used for divergence time estimation. Pair ed-end reads from each accession were aligned to the assembled Cushion willow reference genome using BWA. After mapping, we called and filtered SNPs using VCFtools. The SNPs were annotated using ANNOVAR. Principal component analysis (PCA) was performed with GCTA on SNPs followed by an analysis of population structure using fast STRUCTURE version 1.0. Linkage-disequilibrium (LD) decay was estimated by PopLDdecay, polymorphism levels (θπ), genetic differentiation (FST) and selection statistics (Tajima's D) using ANGSD. and Stairway plot were used in demographic history inference. Site ancestral state (SAS) was estimated using Enredo-Pecan-Ortheus pipeline. Selective sweep analysis was performed using Sweepfinder2. Split time of populations pairs was estimated by fastsimcoal2. GO and KEGG enrichment analyses were performed using the R package clusterProfiler.

For manuscripts utilizing custom algorithms or software that are central to the research but not yet described in published literature, software must be made available to editors/reviewers. We strongly encourage code deposition in a community repository (e.g. GitHub). See the Nature Research guidelines for submitting code & software for further information.

Data

Policy information about availability of data

All manuscripts must include a data availability statement. This statement should provide the following information, where applicable:

- Accession codes, unique identifiers, or web links for publicly available datasets
- A list of figures that have associated raw data
- A description of any restrictions on data availability

Provide your data availability statement here.

Field-specific reporting

Please select the one below that is the best fit for your research. If you are not sure, read the appropriate sections before making your selection.

☐ Life sciences ☐ Behavioural & social sciences ☒ Ecological, evolutionary & environmental sciences

For a reference copy of the document with all sections, see nature.com/documents/nr-reporting-summary-flat.pdf

Ecological, evolutionary & environmental sciences study design

All studies must disclose on these points even when the disclosure is negative.

Study description	We explore the origins of the Hengduan Mountains biodiversity hotspot, we conducted a case study: report a high quality genome sequence of Cushion willow. We then use this as a reference when resequencing 77 individuals from 14 populations across the species'distribution, to characterize its genetic diversity, population structure, demographic history, and footprints of adaptive evolution to the subnival environment.
Research sample	We sequenced a female individual of Salix brachista (cushion willow), and resequenced 77 individuals from 14 populations across the species' distribution. Details of the research sample are present in Supplementary Data 3.
Sampling strategy	We chose a female individual in alpine zone for whole genome sequencing, and selected 77 individuals from 14 populations across the species' distribution for population genetics analysis.
Data collection	The material for DNA sequencing were collected from fresh leaves and then dried in silica gel; for material for RNA sequencing, fresh leaves or whole plant were collected and stored in liquid nitrogen.
Timing and spatial scale	The individuals of cushion willow were collected in growing season or from tissue culture plant.
Data exclusions	In demographic history analysis and population split time estimation, ten individuals not cluster according to geography and was mixed, were excluded from analysis were therefore excluded.
Reproducibility	The whole genome sequencing and resequencing of cushion willow were acquired in high coverage by Oxford Nanopore Techology and PacBio long reads and Illumina short read (Supplementary Table 3). And for resequencing an average depth of 22.1-fold were achieved (Supplementary Data 5). These ensure the reliablity of the whole genome sequencing and resequencing.

Randomization	The individuals selected for resequencing were at least 50 meters apart from each other.
Blinding	This study involve plant genome sequencing and resequencing, blinding was not relevant to this study.

Did the study involve field work? [x] Yes [ ] No

Field work, collection and transport

Field conditions	The field work of this study only involve field material collection, including 77 invididuals from 14 populations of alpine region with elevation range from 2760 to 4540 m of Hengduan Mountains and adjacent areas. Detail conditions are present in Supplementary Data 8.
Location	Details of the sample locations are present in Supplementary Data 3.
Access and import/export	None of the materials are collected from natural protection area, we only collected leaves from the plant and this will not damage the whole plant, thus did violated local laws and regulations and no need for permissions.
Disturbance	We only collected several leaves from the plant, therefore our field work did not disturbance habitats where the species occurred.

Reporting for specific materials, systems and methods

We require information from authors about some types of materials, experimental systems and methods used in many studies. Here, indicate whether each material, system or method listed is relevant to your study. If you are not sure if a list item applies to your research, read the appropriate section before selecting a response.

Materials & experimental systems

n/a	Involved in the study
[x]	[ ] Antibodies
[x]	[ ] Eukaryotic cell lines
[x]	[ ] Palaeontology
[x]	[ ] Animals and other organisms
[x]	[ ] Human research participants
[x]	[ ] Clinical data

Methods

n/a	Involved in the study
[x]	[ ] ChIP-seq
[ ]	[x] Flow cytometry
[x]	[ ] MRI-based neuroimaging

Flow Cytometry

Plots

Confirm that:

[x] The axis labels state the marker and fluorochrome used (e.g. CD4-FITC).

[x] The axis scales are clearly visible. Include numbers along axes only for bottom left plot of group (a 'group' is an analysis of identical markers).

[ ] All plots are contour plots with outliers or pseudocolor plots.

[ ] A numerical value for number of cells or percentage (with statistics) is provided.

Methodology

Sample preparation	To avoid the risk of error due to instrument drift, we simultaneously chopped the test samples and reference sample. Fresh leaves of tested samples were placed in plastic petri dish containing WPB for 30 minutes, and then were chopped using a razor blade, passed each sample through a 30-µm filter, and added staining solution for 15 minutes in dark.
Instrument	BD FACScalibur.
Software	Cytomics Expo32.
Cell population abundance	cell sorting not employed.
Gating strategy	Live gating is based on forward and side scatter . Following gating is placed around populations of cells with common characteristics based on staining marker with positive and negative controls.

[x] Tick this box to confirm that a figure exemplifying the gating strategy is provided in the Supplementary Information.
