# Supplementary material for: Genome-wide analysis of Cushion willow provides insights into alpine plant divergence in a biodiversity hotspot
Source: Nat Commun. 2019 Nov 19;10:5230. doi: 10.1038/s41467-019-13128-y (PMC6864086; doi:10.1038/s41467-019-13128-y)
Supplement: Supplementary file 4 — Description of Additional Supplementary Files [file 41467_2019_13128_MOESM4_ESM.docx]

**Description of Additional Supplementary Data**

File Name: Supplementary Data 1

Description: Classification of repetitive DNA in the *Salix brachista* genome.

File Name: Supplementary Data 2

Description: GO enrichment of *Salix brachista* expanded gene families.

File Name: Supplementary Data 3

Description: GO and KEGG enrichment of *Salix brachista* rapidly evolved gene families.

File Name: Supplementary Data 4

Description: GO and KEGG enrichment of *Salix brachista* species-specific gene families.

File Name: Supplementary Data 5

Description: GO and KEGG enrichment of *Salix purpurea* and *Salix suchowensis* expanded gene families.

File Name: Supplementary Data 6

Description: Gene numbers of gene families involved in the top enriched KEGG/GO term in three *Salix* species examined.

File Name: Supplementary Data 7

Description: Information on populations and individuals of *Salix brachista* used in genome sequencing and re-sequencing.

File Name: Supplementary Data 8

Description: Whole genome resequencing statistics and quality control results.

File Name: Supplementary Data 9

Description: Summary and mapping statistics of sampled *Salix brachista* individuals.

File Name: Supplementary Data 10

Description: Mean population fixation index and corresponding spatial distance.

File Name: Supplementary Data 11

Description: Inferred demographic parameters of the best-fitting demographic model of six population pairs of *Salix brachista* based on 100 bootstrap replicates.

File Name: Supplementary Data 12

Description: Independent-samples t test for environment variable and measure of highland and lowland *Salix brachista* as well as highland and lowland LJ populations.

File Name: Supplementary Data 13

Description: Significant selected region for highland and lowland LJ populations of *Salix brachista*.

File Name: Supplementary Data 14

Description: Population parameters statistics for *Salix brachista* populations.

File Name: Supplementary Data 15

Description: Environmental factor values for highland and lowland *Salix brachista*.
