# Supplementary material for: Genome-wide analysis of Cushion willow provides insights into alpine plant divergence in a biodiversity hotspot
Source: Nat Commun. 2019 Nov 19;10:5230. doi: 10.1038/s41467-019-13128-y (PMC6864086; doi:10.1038/s41467-019-13128-y)
Supplement: Supplementary file 2 — Peer Review [file 41467_2019_13128_MOESM2_ESM.pdf]

Reviewers' comments:

Reviewer #1 (Remarks to the Author):

Review of "Origins of the Hengduan Mountains biodiversity hotspot: insights from a genome-wide evolutionary analysis of Cushion willow" for Nature Communications

This manuscript describes a whole genome sequence for a dwarf willow, Salix brachista, together with genome resequencing of 77 individuals from populations in the Hengduan Mountains in China. The genome assembly is impressive and highly contiguous, and some of the analyses are interesting and reveal some insights about the evolutionary history of this species. However, I thought there were several quite serious flaws with the interpretation of the genomic data, as detailed below. In particular, I disagree with the premise that this manuscript demonstrates "alpine environmental responses, e.g., to hypoxia and DNA damage." (lines 47-48). Also, given that the manuscript focuses on a single species with potentially unusual life history, I disagree with the assertion that the manuscript provides new, unambiguous insights about species diversification in the Hengduan Mountains. I recommend that the authors restrict their range of inference to this single species, reduce their reliance on hand-selected categories of genes to weave adaptationist stories, and tighten up their interpretation of the genomic data. The basics of an excellent manuscript are here, but it needs further work.

Major Issues

1. The GO enrichment analyses were not convincing. The discussion centers around phenotypes that can be interpreted in terms of adaptation to high elevation, but the cited categories are hand-selected from among many categories with significant enrichment, and the cited categories also have marginal statistical significance and do not involve a large number of genes. This is not a robust analysis and it inappropriately layers interpretations onto the data that are not well supported by statistics. At the very least, the authors should find a way to support their interpretations more directly, rather than referring the reader to six supplementary tables in which the cited GO categories are buried among a multitude of categories that are irrelevant to their interpretations.
2. The evidence for whole genome duplications (WGD) within the willow lineage, as indicated in Fig. 2C, is very weak and poorly supported by the presented analyses. If the two Salix species share a very recent whole genome duplication, the peak should be higher than the older Salicacae whole genome duplication. It should also be very obvious from the dotplot comparison to P. trichocarpa. Rather than representing a true WGD, this likely reveals large-scale segmental duplications, or the presence of divergent haplotypes that have been included in the main genome assembly erroneously. I think the latter is the most likely explanation, as explained above.
3. There does not appear to be any robust protection from Type I error in the Fst outlier analysis, which was apparently done without protection for multiple testing or any of the other artifacts that plague such analyses.
4. The outlier Fst analysis also suffers from the same limitation as the GO enrichment gene family analysis. The highlighted genes and categories are the ones that support the premise, not the ones with the most robust statistical significance. At the very least, some phenotypic data supporting these hypotheses would be necessary to demonstrate the robustness of the conclusions.
5. There is no justification provided for the filtering of the SNP data from GATK, and this is a critical issue since it has a dramatic impact on the LD estimates and coalescent simulations that were performed with these data. Hard filtering of SNPs, as applied here, is generally not recommended, particularly when downstream analyses depend upon low false positive and false negative rates in SNP identification.
6. The estimated genome size is 486 Mb based on flow cytometry, and this closely matches the total length of the assembly, which is good. However, this result conflicts drastically with the kmer-based size estimate of 401 Mb. As stated in the manuscript, this discrepancy could be due in part to Illumina bias, but this would mean that large portions of the assembly would be derived from PacBio only

sequence. Given that the authors must have aligned the Illumina reads back to the assembly, they should be able to estimate how much of the genome was not covered by Illumina. This should be reported.
7. If it is true that 80 Mb of the genome has no Illumina coverage, then the error correction would not have been applied as in the rest of the genome. This would result in an overestimate of polymorphisms for those parts of the genome. This could result in several artifacts, including failure to remove alternative haplotypes with the very stringent filtering that was applied (98% identity). I suspect that this genome assembly still contains alternative haplotypes, and that accounts for the discrepancy in kmer and flow cytometry genome size estimates.
8. The dot plot in Fig. S7 is quite interesting, and seems to show some large-scale duplications in cushion willow, and an apparent lack of the large discrepancy found between Chr1 and Chr16 in S. suchowensis. However, it is very hard to tell due to the high background (the vertical lines). Please produce a clearer version of this figure and discuss the genome comparisons more thoroughly. It would be quite interesting if cushion willow also has the Chr1 fusion that is present in Populus but absent in S. suchowensis and S. purpurea.

Minor Issues

9. Lines 112-114. The rationale for concluding that the genome is highly repetitive based on the heterozygosity rate of 0.2% is not clear. Are the authors concluding that this level of polymorphism is too low to account for the second peak in the kmer distribution? This has not been demonstrated, and in my experience this assumption would be untrue. In any case, the rationale for this conclusion needs to be more clearly stated.
10. Lines 137-138. The manuscript states that 97.85% of genes were annotated based on sequence similarity to annotated proteins in public databases. This is likely to be misleading, since many of the "annotations" in public databases are probably "gene of unknown function" or something similar. I think the authors mean that 97.85% of the predicted proteins matched a predicted protein in public databases. "Gene of Unknown Function" is not an annotation. The functional annotations should be quantified more precisely, or the statement should be clarified.
11. Lines 160-165. The analysis reported here is not a robust demonstration of monophyly of Salix and Populus, which is not really in doubt in any case. I recommend deleting this paragraph. However, I agree that the phylogeny is a useful framework for presenting the gene family results, so Fig. 2b is helpful.

Reviewer #2 (Remarks to the Author):

Comments to the authors

The authors report a high-quality, chromosome-scale genome of Cushion willow (Salix brachista), which they use as a reference when resequencing 77 individuals from 14 populations across the species’ distribution range (incl. sites in the Hengduan Mountains region and neighbouring regions), to characterize the species´ genetic diversity, population structure, demographic history, and footprints of adaptive evolution to the subnival environment. The authors interpret their results in the context of explaining high plant species richness within the HDM.

I would like to express my great appreciation to the authors for this extensive study, which aims at bridging the gap between genomics, population genetics/genomics, and species richness patterns, at the backbone of a geologically active mountainous region. I found the manuscript very interesting to read, but also came across several issues that will deserve some careful attention. A very recent paper (Muellner-Riehl, 2019), dealing with the geographic region which is also the focus of this manuscript, tackles many of these issues and provides suggestions for future research avenues, some of which ideally could be taken up by this study.

In the following, where appropriate, I will refer to the line numbers as given in the merged pdf file. I hope that my comments will be helpful for the authors.

Major issues

1. The authors interpret their population-level, infra-specific results in the context of explaining high plant species richness within the HDM. The connection between the two (which would be speciation) is currently not convincingly presented in the manuscript. There have been some recent attempts to view speciation and the origination of high levels of species richness in the same context. For example, the recently presented Mixing-Isolation-Mixing (MIM) model of speciation (He et al. 2018; compare comments by Abbott, 2018, and Rieseberg, 2018), supports that speciation may involve repeated cycles of admixture, followed by geographic isolation until complete isolation is finally achieved. By permitting intermittent gene flow during speciation, MIM cycles can potentially generate species at higher rates than just by geographic isolation, which in turn means that speciation and the origination of high biodiversity levels in a region may be viewed in the same context framework (He et al., 2018). Translated to the Hengduan Mountains region and Salix, one would postulate that the climatic fluctuations of the Quaternary would have forced populations to shift their distribution ranges (thus being repeatedly connected and disconnected again), possibly with a species-pump acting. See Muellner-Riehl (2019) for more details.

2. The authors argue for uplift-driven diversification (as many other authors have done before) in an ad hoc manner, but they don´t test this. What could be alternative explanations for the patterns observed? How could different scenarios be compared and tested? What is the most likely explanation? (see Muellner-Riehl 2019 for some suggestions). Connected to the issue raised above, the relation of population-level, infra-specific differentiation and (species-level) diversification is not apparent.

3. Caution needs to be taken concerning the naming and delimitation of the mountain regions. The Qinghai-Tibetan Plateau (QTP) proper, the Himalayas, and the Hengduan Mountains region have to be viewed separately, as they have quite different geological (uplift) histories, different orientation, were/are differently impacted by climatic conditions (e.g., monsoonal influence; glaciation), and have different mountain morphology, the latter resulting in contrasting scenarios for population connectivity during either cooling or warming phases through time. See Muellner-Riehl (2019) for more details.

4. The authors investigated GO enrichment for pathways related to DNA damage and repair for Salix brachista, and looked for footprints of adaptive evolution in the genome. It would be interesting to know how Salix brachista does compare to the other Salix species investigated in this study.

5. In several instances, the authors make claims about a connection between adaptation and "climate change during QTP uplift" and "the onset of harsh subnival environmental conditions". This is problematic for two reasons: First, the QTP uplift predates the Hengduan Mountains uplift (see my previous comment about correct naming and delimitation of mountains regions of the Tibeto-Himalayan Region (THR); see also Muellner-Riehl, 2019), and second, it is not clear what kind of "climate change" the authors are referring to. This is one of several examples where care needs to be taken for a proper use of terminology and handling of abiotic factors.

6. Of a somewhat similar problematic is the discussion of population bottlenecks with regard to glaciations. Information needs to be provided about the regional extent of these glaciations (into the species distribution range), how these glaciations would have possibly impacted vegetation cover in the HDM, either directly by glacial cover, or a drop in temperature. The age for the LGM given is too young, and it is known that the maximum glacial extent in the THR was not at the time of the LGM, but pre-dated the LGM (see Muellner-Riehl 2019 and references therein for details), thus rendering the time of the global LGM as not specifically relevant for the THR.

7. More details should be given concerning the molecular dating analysis (use of priors, age of fossils, setting of constraints).

8. Concerning environmental heterogeneity between high and lower elevation sites, more information should be given about the rationale behind using the climatic parameters finally chosen, and not other abiotic parameters, like soil, or other measures of geodiversity.

Minor issues (and details)

Abstract:

29-31: "The Hengduan Mountains (HDM) is a biodiversity hotspot exhibiting exceptional plant diversity, particularly for alpine plants. However, the origins of this high alpine plant richness remain largely unknown". This is not entirely true (N-S orientation of HDM enabling easier migration during climatic changes, less glaciation impact, monsoonal influence, steep gradients with shorter migration distance during climatic fluctuations in the Quaternary, etc.). See Muellner-Riehl 2019 and references therein. Apply necessary text changes in all other relevant parts of the ms as well.
35: genome "sequence"? re-phrase
38: "topographic features of the HDM are associated with…" Which features exactly? Be more precise. Testable?
40: use of "sky islands" here and elsewhere - Please provide information which definition you apply for the term "sky islands". Not all readers will be familiar with this term, and it is applied in differently strictly ways of its definition.
41: "heterogeneous environments across different elevations" Be more precise (heterogeneity measured by? Which elevations?).
44: Use of "subnival" versus "alpine" – please check for consistency of the use of these terms throughout the ms
47-50: "These findings indicate that the HDM served as an evolutionary cradle and driver of diversification of alpine species during its history, which contributed to the very high level of plant diversity characterizing this region today." Strictly speaking, this was not investigated here in the study, the latter which focused on a single species. See my comment under "major issues".

Introduction:

53: "Hengduan Mountains (HDM) biodiversity hotspot" This is not the official name of the biodiversity hotspot. It is called "Mountains of Southwest China", and includes the Hengduan Shan.
57: provide citation for species number
58: alpine and subnival – see my comment before
61-63: Be more specific about the hypothesis. The hypothesis also includes a temporal component and climatic fluctuations, see the "mountain-geobiodiversity hypothesis" by Mosbrugger et al. 2018 and an updated version in Muellner-Riehl 2019.
65-66: Important reference missing here, where potential drivers were actually tested:
Ebersbach, J., Muellner-Riehl, A.N., Favre, A., Paule, J., Winterfeld, G. & Schnitzler, J. (2018). Driving forces behind evolutionary radiations: Saxifraga sect. Ciliatae in the region of the Qinghai-Tibet Plateau. Botanical Journal of the Linnean Society 186, 304-320.
Ebersbach, J., Schnitzler, J., Favre, A & Muellner-Riehl, A.N. 2017. Evolutionary radiations of the species-rich mountain genus Saxifraga L. BMC Evolutionary Biology 17, 119.
67: "important questions" – please state which ones.
68-73: Population genomics analysis and "causes of high alpine plant diversity within the HDM" are two pairs of shoes. See my comment under major issues.
74-75: Provide information about how many species of Salix do occur in the HDM. What are the closest relatives of S. brachista and what is known about hybridization?

Results and discussion:

165: You may want to consult Peter Stevens´s Angiosperm Phylogeny Website (go to "Salicaceae") at http://www.mobot.org/MOBOT/Research/APweb/welcome.html to take a look at other studies which have been conducted on Salicaceae phylogenetics and molecular clock dating.
179-198: How do patterns compare to other species of Salix included in this study?
207: alpine – see my previous comment about the use of "subnival" and "alpine"
208: provide information and citation for this high temperature
220: WGD events – at this stage is is unclear to the reader where (which specimen/species) this was investigated
224-225: unclear to which level this refers to (ancestor of ?)
231: genetic structures – which ones?
234: citation 28 missing here
238: recent burst of further gene duplication – what is exactly meant by this?
240-241: "footprints of adaptive evolution in the Cushion willow genome." – anything known about the other Salix species included in this study? How do they compare in terms of ecology/biology?
243-244: „may have facilitated species-specific adaptations driven by climate change during QTP uplift and the onset of harsh subnival environmental conditions" – what kind of climate change are you referring to? (attention: uplift of the QTP and adjacent mts. was not the cause of the origination of the monsoon system, and the QTP had an average elevation of 3-4 km already since the Eocene; see reviews by Favre et al. 2015, Renner et al., 2016, Spicer, 2017; Botsyun et al., 2019).
Favre, A., Päckert, M., Pauls, S.U., Jähnig, S.C., Uhl, D., Michalak, I., et al. (2015). The role of the uplift of the Qinghai-Tibetan Plateau for the evolution of Tibetan biotas. Biol. Rev. Camb. Philos. Soc. 90, 236–53. doi:10.1111/brv.12107.
Renner, S.S. (2016). Available data point to a 4-km-high Tibetan Plateau by 40 Ma, but 100 molecular-clock papers have linked supposed recent uplift to young node ages. J. Biogeogr. 43, 1479–1487.
Spicer, R. A. (2017). Tibet, the Himalaya, Asian monsoons and biodiversity - In what ways are they related? Plant Div. 39, 233–244.
Botsyun et al. (2019). Revised paleoaltimetry data show low Tibetan Plateau elevation during the Eocene. Science Vol. 363, Issue 6430, eaaq1436. DOI: 10.1126/science.aaq1436
278: monsoon system in the QTP – please specify. The QTP proper and the western Himalayas are almost entirely beyond the reach of the summer monsoon.
292: generation time of three years – seems rather low given the harsh conditions under which these plants grow. Please provide citation, upon which literature (or personal observation) this is based?
295: environmental change in the QTP – see my comments under major issues. The populations investigated don´t occur in the QTP proper. Please check - there is no Fig. 3f (should this be 3e?).
303-304: LGM age given is too young. If this refers to the global LGM (terrestrial), see Hughes, P.D., and Gibbard, P.L. (2015) A stratigraphical basis for the Last Glacial Maximum (LGM), Quat. Int. 383, 174–185. doi.org/10.1016/j.quaint.2014.06.006.
306-307: adapted to environmental change – please re-phrase. Species are not adapted to change, but to certain conditions. Uplift of the HDM – which uplift are you referring to? Specify.
307-311: Needs re-phrasing, see Muellner-Riehl 2019. 10 is not a glaciation reference (see Muellner-Riehl 2019 for glaciation information and literature). Also, it is not clear how this would fit with Fig. 3e.
317: robust clade – definition of „robust"?
326: How do the age estimates of this study compare with previous age estimates by other authors?
328-329: "This overlaps with the confidence interval for the divergence time of Cushion willow, suggesting that the species origin was associated with tectonic changes in the HDM." This is an ad hoc assumption, do you have real evidence for this? How could this be tested, what are alternative explanations? See Muellner-Riehl 2019 ("clearly defined phylogeographic hypotheses may be tested rigorously, instead of applying explanations that fit the observed results ad hoc (i.e., after analysis of the data), as has been done in the past.").

336-338: See some of my previous comments about the connection between population divergence within species and species richness. As presented now, the direct link (if it does exist at all – not proven yet for this region, but some studies pointing towards this) is not convincingly presented. The study by Xing and Ree investigates species richness / is based on species-level and above, not popuation divergence, but they provide interesting thoughts concerning the HDM. See also Muellner-Riehl 2019.
353: some unique terms – meaning unclear
355: Where appropriate in the text, it would be interesting to learn more about the potentially different impact of competition (from other co-occurring plant species) that the more lowland versus subnival populations of S. brachista might be subjected to, also given the fact that the selective pressure observed seems weaker in high elevation sites.
374 and following: The situation described is the one of nowadays, formerly it was colder, populations moved down and were better connected. See Muellner-Riehl 2019.
384: elevational gradients
386: diverse heterogeneous habitats – meaning unclear, specify
388: and following: Again, please see the problematic with this statement as outlined previously. It is not merely populations being uplifted with the mountain as it rises, it is climatic fluctuations making plant populations move and being separated or connected at various times on and across mountains.

Methods:

393: Please provide voucher information on the specimen investigated (sequenced), collector and collector number, date, location, deposited at which herbarium, etc.
442: Here and elsewhere in the ms, please take care of properly setting spaces
455: artefact (though artifact also possible)
518: with the GTRGAMMA
519: Here and elsewhere in the ms, please take care of using hyphens in compound words
526: generated a maximum; which evolutionary model was used?
549-550: This sentence is not clear, please clarify and re-phrase. Certainly, you would have first run the actual NJ analysis, and then the independent boostrap analysis (the latter which is based not on the original data matrix, but the pseudo-replicates).
560 and following: Something is wrong with this sentence; phrasing and meaning of sentence unclear. Please provide the age of the fossils, based on the original primary literature.
564: Unclear – was a maximum root age set? Was a range of between 48 and 52 tested? Please specify in the text the rationale behind the setting of temporal constraints, and provide more detailed information about the priors.
602 and following: Provide information about the rationale behind considering only climatic parameters (and the ones you have chosen). What about soil or other geodiversity parameters? What would be the potentially most important determinants of occurrence for S. brachista?
609: Shannon, Simpson

References:

Need to be carefully checked for typos (diacritics in author names), formatting, use of upper and lower case, etc. throughout.

Figure captions:

Figure 1: a) and b) flowers, not flower; m a.s.l.?
1f) Scheme or schematic? (both terms possible) This picture is very simplified, and does not consider mountain morphology (which is different for HDM versus Himalayas or QTP). You may consider using a real cross-section (compare Muellner-Riehl 2019) and then simplify it (smoothing the lines). Be aware that in the Himalayas/QTP, area size for alpine plants will increase at higher elevation.
Figure 2: c) Please check whether the legend in the right-hand upper corner is correct. Insert "events"

after “(WGD)”.
Figure 3: “tines” correct? Last Glacial Maximum. Concerning timing of the global terrestrial LGM, see my previous comments (for 303-304) and Hughes and Gibbard 2015.

Figures:

Improve readability of fonts used (e.g., Fig. 1e, river and mountain names)

Final:
- Carefully read, check and correct for clarity and logic of English language and use of scientific terms throughout the manuscript.
- You may want to read (and cite) the following publication for comparison with your study:
Cai, L. [et al. 2018], Xi, Z., Amorim, A. M., Sugumaran, M., Rest, J. S., Liu, L., & Davis, C. C. 2018. Widespread ancient whole genome duplications in Malpighiales coincide with Eocene global climatic upheaval. https://nph.onlinelibrary.wiley.com/doi/epdf/10.1111/nph.15357

Alexandra Muellner-Riehl

Reviewer #3 (Remarks to the Author):

This is a very interesting and impressive study in which the authors conduct an ambitious population genomic analysis for a plant species native to an understudied biodiversity hotspot. The authors generated the first reference genome for Salix brachista and it important to note that they have been successful in scaffolding their reference genome into what appear to be the 19 chromosome of this species. To do so they have used state-of.the-art methodology such combining long and short reads for the assembly and HiC for the scaffolding. They also propose a very interesting set of 77 re-sequenced accessions sampled in localities with very different environmental and climatic conditions.

I would like to felicitate the authors for having undertaken this comprehensive analysis of an interesting non-model plant species. I am convinced that these new genomic resources will be of great interest to the community. However, I have been disappointed by a series of imprecisions, inaccuracies through my reading of the analyses of this new data. But I am convinced that if the authors can address the issues listed in my review to meet the standard of the field, they could finalize a great contribution to the plant population genomic literature.

Genome assembly.

l. 114: The authors state here that given the measured heterozygosity of 0.2%, the genome must be highly repetitive. It seems that this is deduced from the joint observation of the k-mer distribution and the observed heterozygosity but it is unclear from the text what justifies making this statement. Please describe the causal relations implied here.

Figure 2c: The annotation of this figure suggest that each peak is associated to a whole-genome duplication. I wonder, however, what effect the duplication of a single or two chromosomes would have on such distributions. For example, could it be that the most recent peaks in the S. brachista distribution be caused by the duplication of the Hic groups 8/19 and 9/12 only? Is the term whole-genome duplication applied loosely to refer to a duplication of a substantial part of the genome? Because the locations of each pairs of paralogues used to calculate the Ks distribution are known, I assume it should be possible to find out which chromosomes are associated with each of the three peaks.

Phylogenetics

PAML analyses: the authors write in the M&Ms that they have estimated the Ka/Ks ratio for each branch in the phylogeny (note that there is more than one ratio per branch in both models). How does the number of positively selected gene in S. brachita (1582, l. 175) compares to the distribution of equivalent numbers on all other branches. This is important to help the reader evaluate whether there is a particular signal of adaptation on the external branch leading to S. brachita or whether this very large proportion (1582/11288) could be an artefact of the statistical method employed here.

l.180: significant GO enrichment: what set is enriched, PSG? The authors are not being precise here.

l.182-183: in Sup Table 8 (line 252), the GO term "regulation of response to stimulus to DNA damage stimulus" is associated with a q-value of 0.96, that is non-significant. At line 180 the author claim this to be significant. Is there a mistake somewhere?

l.183-185: same here, all these terms are associated with q-values > 0.05 according to Sup. Table 9). It looks like the authors have based their assessment of significance on p-values not corrected for multiple testing.

l.185-again, same problem for the PSG class. None of these GO terms seems to be significant at the 5% level after correcting for multiple testing. Furthermore, the classes reported by the authors in this paragraph are not even the one associated with lowest q-values (see Sup Table 8,9,10). Rather, it seems that the authors have cherry-picked these classes to be consistent with their a priori hypothesis of an adaptation to UV-radiation-induced damage. My conclusion for this paragraph is that either the authors manage to make sense of the list of significant GO terms (i.e. q-value < 0.05) or the whole paragraph should be removed. My personal recommendation is to remove this paragraph.

l-189-192: this result has no relevance given my comments above.

l.193-195: the GO term "anthocyanin accumulation […]" is not even present in Sup Table 8 and 9".

No single sentence in this paragraph is backed by the results presented in the manuscript.

l. 240-241: This assertion should be reformulated to clearly indicate that it is based on results from PAML only. Given the very large number of genes identified as significant and the poor treatment of the GO analysis, I am not sure how much confidence can be given to this statement.

l-241->end of paragraph: no results are presented that show a significant enrichment of PSG genes in species-specific or species-specific-expanded gene families. The conclusions reached here a pure speculation.

Genetic structure and demographic analysis

-) Please add a table with population specific values of theta, Taj D , Fst calculated by excluding genic regions from the data.

-) authors should report the cross-validation error plot for the structure results.

-) Model-based phylogenetic inference as presented in sup Figure 9 is not an acceptable representation of intraspecific variation data. This is because recombination can occur within each locus/gene and violate the assumption of a bifurcating tree assumed by the method. Based on this, I suggest to remove this analysis from the manuscript. Also it does not provide additional information to

the structure and pca results. (I feel less strongly about the NJ tree because it can be seen as a clustering method and not an explicit model-based inference method)

-) tree mix does not infer a phylogenetic tree because the units here are not species but populations. Rather it infers the patterns of split and mixture in the history of a set of populations.

-) line 286. I agree that the Fst can be influenced by the distance/migration between populations in this study. But it could also be affected by the history of recent population splits (see Figure 3d). See my comment below about model-based demographic inference .

-) stairway plot analysis: this is a very interesting analysis, which seems to have been conducted very carefully (SFS estimated with ANGSD, re-estimation of the mutation rate). How is this method affected by the significant population differentiation reported in this manuscript? The authors do not specify which populations/accessions have been used to create the SFS used for the stairway analysis. I would like to see a figure with one stairway plot for each identified cluster in the structure analysis (Figure 3a).

-) Figure 3e: please add a panel showing the paleoclimatic record for the time range presented on the x-axis of figure 3e (a continuous variable indicating temperature or a proxy of temperature). This will allow a better appreciation of whether the estimated Ne values really correlate with past climatic events.

Line 295: there is no figure 3f

Line 302: The confidence interval around the Ne estimates shrinks to 0 for that period. Is there a good explanation for this? Can we trust the results of this method for the time interval [0;20000 generations]?

Line 307-311: this sentence sounds like the HD mountains were adapted to subnival conditions. Is this what the authors meant to write? Did they mean that the Cushion willow was well adapted to the HD mountains during glacial periods?

Origin of cushion willow associated with HDM tectonic events.

It is not acceptable to use phylogenetic reconstruction to evaluate the demographic history of these populations. As mentioned before this is because there is not a single bifurcating tree underlying this genomic data but many (due to recombination), see https://www.nature.com/articles/s41559-018-0717-x for an example. Instead the authors should estimate the age of the splits between the different genetic clusters (or main clusters) using either the software dadi (https://bitbucket.org/gutenkunstlab/dadi) or fastsimcoal2 (http://cmpg.unibe.ch/software/fastsimcoal2/). Note that the authors should try to construct the SFS using neutral sites (i.e excluding genic regions for example)

Selection on Cushion willow at high and low altitudes.

In its current form the genome scan for selection to higher altitudes does not meet the standards of the field. The authors should compare the distribution of the statistic(s) used for scanning the genome to a null distribution obtained from a neutral demographic model. Such a model can estimated using one of the two tools mentioned above (dadi or fastsimcoal2). The authors should use these models to define a statistical threshold indicating how extreme the genome scan statistics should be expected to be given the demographic history of the cushion willow. Also note that tests like HapFLK, EHH (his, XP-EHH, implemented in selscan) and the CLR test implemented in the software sweepfinder are likely to be more powerful to detect selective sweeps than the statistics used by the authors so far.

Please, also indicate what is known about the breeding system of the species. Is it an outcrosser or a selfer?

Minor

149: Where are the references to these datasets?

Figure 2a: The tracks “iii” and “iV” of the circus plot can not be read from a printed version. Even by zooming in track “iv” does not seem to display any information.

Dear editor and reviewers:

We would like to thank the three reviewers for their positive and constructive comments and suggestions which have been very helpful in revising and improving our manuscript. We have carefully revised our manuscript and provide below point-by-point responses (in blue) to all comments received. A major change in our revised manuscript is the inclusion of an updated Cushion willow genome to address the problem of the large discrepancy between *k*-mer-based estimated genome size (~400 Mb) and assembled genome size (~480 Mb). We present a PCR-free based *k*-mer estimate of the genome size of Cushion willow which confirms it to be ~400 Mb. We further conducted Oxford Nanopore Technology (ONT) long read sequencing, and re-assembled the Cushion willow genome based on ONT long reads and polished by PacBio and Illumina reads. As a result, we found multiple small fragments of redundancy in the previous assembly, which might be caused by alternative haplotypes. The contiguity of the updated Cushion willow genome assembly is significantly enhanced, with contig N50 of ~9.5 Mb and scafford N50 of ~17.9 Mb, and in line with *k*-mer-based genome size estimation. We replaced all statistics and results of the updated genome assembly in the revised genome. We then repeated all analyses based on the updated reference Cushion willow genome and yielding results similar to those reported previously, addressing concerns and comments of reviewers in the process (e.g., stricter variant and SNP calling criteria). All revised portions of the manuscript are marked in red.

Point-by-point response to reviewer comments:

Reviewer #1 (Remarks to the Author):

This manuscript describes a whole genome sequence for a dwarf willow, Salix brachista, together with genome resequencing of 77 individuals from populations in the Hengduan Mountains in China. The genome assembly is impressive and highly contiguous, and some of the analyses are interesting and reveal some insights about the evolutionary history of this species. However, I thought there were several quite serious flaws with the interpretation of the genomic data, as detailed below. In particular, I disagree with the premise that this manuscript demonstrates "alpine environmental responses, e.g., to hypoxia and DNA damage." (lines 47-48). Also, given that the manuscript focuses on a single species with potentially unusual life history, I disagree with the assertion that the manuscript provides new, unambiguous insights about species diversification in the Hengduan Mountains. I recommend that the authors restrict their range of inference to this single species, reduce their reliance on hand-selected categories of genes to weave adaptationist stories, and tighten up their interpretation of the genomic data. The basics of an excellent manuscript are here, but it needs further work.

Response: Thanks very much for your comments. Regarding the issue of alpine environmental response, Cushion willow is a typical alpine plant in the HDM and adjacent areas which encounters a subnival environment; it is reasonable to expect, therefore, that adaptive footprints related to alpine adaptation might be present in its genome. We therefore searched for such genomic signals of adaptation based on an analysis of gene families. In the former version of the manuscript, we included some GO or KEGG terms with significant p values (< 0.05) but not significant q values (< 0.05). In the revised version, we only discuss possible alpine adaptive footprints based on terms with significant p values (< 0.05) corrected for multiple comparisons using the method of Benjamini and Hochbergsignificant. Since there were no significant enriched GO/KEGG terms in positively selected genes identified by the branch-site model using CodeML in PAML package, we removed this analysis as suggested by reviewer #3.

Regarding the origin of HDM biodiversity richness, it is true that we only conducted a case study of one species - Salix brachista - a dominant component of many subnival assemblages in the HDM. Our results indicate that diverse isolated heterogeneous habitats on sky islands, together with climatic fluctuations that have possibly caused recurrent cycles of admixture and isolation between some populations (MIM cycles), were likely important drivers of intra-specific population diversification in Cushion willow, and by extension, other alpine species with similar distributions and histories in the HDM. Because intraspecific divergence is often a first step in speciation, we propose that such factors were also likely important contributors to the generation of high alpine species diversity in the HDM.

Major Issues

Comment 1: The GO enrichment analyses were not convincing. The discussion centers around phenotypes that can be interpreted in terms of adaptation to high elevation, but the cited categories are hand-selected from among many categories with significant enrichment, and the cited categories also have marginal statistical significance and do not involve a large number of genes. This is not a robust analysis and it inappropriately layers interpretations onto the data that are not well supported by statistics. At the very least, the authors should find a way to support their interpretations more directly, rather than referring the reader to six supplementary tables in which the cited GO categories are buried among a multitude of categories that are irrelevant to their interpretations.

Response: In the revised manuscript, we only report and discuss alpine adaptation based on enriched GO/KEGG terms with significant p values (<0.05) corrected for multiple comparisons. We consider it is logical to do this because Cushion willow is an alpine plant which grows in subnival habitats and is likely, therefore, to have evolved adaptations to such conditions with signatures of such adaptation in its genome. Morphological variation between Cushion willow populations is minor, except for redness of branchlets, which differs between elevations. We measured the anthocyanin concentration in lowland and highland Cushion willow, as well as greenhouse maintained individuals, and observed it to be higher in high elevation individuals., where it may function to protect plants against UV radiation.

Following the suggestion of Reviewer #2, we also compared GO/KEGG enrichment results of Cushion willow with two other *Salix* species (*S. purpurea, S. suchowensis*) analyzed in this study, and did not find significant enriched GO/KEGG terms related to alpine adaptation (i.e., DNA repair, response to hypoxia) in either of these species (supplementary tables 10-13).

Comment 2: The evidence for whole genome duplications (WGD) within the willow lineage, as indicated in Fig. 2C, is very weak and poorly supported by the presented analyses. If the two Salix species share a very recent whole genome duplication, the peak should be higher than the older Salicacae whole genome duplication. It should also be very obvious from the dotplot comparison to P. trichocarpa. Rather than representing a true WGD, this likely reveals large-scale segmental duplications, or the presence of divergent haplotypes that have been included in the main genome assembly erroneously. I think the latter is the most likely explanation, as explained above.

Response: Using the updated genome assembly, we re-identified orthologous genes and repeated the WGD analysis. We only observed an obvious peak representing the common WGD event in Salicaceae, indicating that the additional peaks observed previously in Cushion willow were caused by alternative haploid or redundancy in the previous genome assembly (please see detail explanation in response below). Consequently, we modified the text accordingly.

Comment 3: There does not appear to be any robust protection from Type I error in the Fst

outlier analysis, which was apparently done without protection for multiple testing or any of the other artifacts that plague such analyses.

Response: In the revised version, we did not use *Fst* and θπ outliers to identifiy genomic regions under selection. As suggested by Reviewer #3, we used instead a composite likelihood ratio statistic (CLR) method implemented in Sweepfinder2.

Comment 4: The outlier Fst analysis also suffers from the same limitation as the GO enrichment gene family analysis. The highlighted genes and categories are the ones that support the premise, not the ones with the most robust statistical significance. At the very least, some phenotypic data supporting these hypotheses would be necessary to demonstrate the robustness of the conclusions.

Response: We did GO and KEGG enrichment and reported enriched terms with significant *p* values (<0.05) corrected for multiple comparisons in the revised manuscript. We also added some phenotypic data, i.e., anthocyanin concentration data for Cushion willow individuals from two different elevation sites (2950 and 3950 m), as well as individuals maintained in a greenhouse.

Comment 5: There is no justification provided for the filtering of the SNP data from GATK, and this is a critical issue since it has a dramatic impact on the LD estimates and coalescent simulations that were performed with these data. Hard filtering of SNPs, as applied here, is generally not recommended, particularly when downstream analyses depend upon low false positive and false negative rates in SNP identification.

Response: We agree that filtering of the SNP data has a dramatic impact on subsequent analyses. In the manuscript, we therefore did not use hard filtering anymore; instead, we used much stricter variant and SNP calling and filtering criteria in the manuscript. These criteria were used in a recent similar genome-wide analysis in *Populus* (Wang, J. et al. 2018. A major locus controls local adaptation and adaptive life history variation in a perennial plant. Genome Biology 19:72.). We found a similar number of SNPs (~6.5 M) by SNP calling as before, but using the stricter filtering criteria, we obtained ~1.6 M high quality SNPs instead of the previous ~2.9 M.

Comment 6: The estimated genome size is 486 Mb based on flow cytometry, and this closely matches the total length of the assembly, which is good. However, this result conflicts drastically with the kmer-based size estimate of 401 Mb. As stated in the manuscript, this discrepancy could be due in part to Illumina bias, but this would mean that large portions of the assembly would be derived from PacBio only sequence. Given that the authors must have aligned the Illumina reads back to the assembly, they should be able to estimate how much of the genome was not covered by Illumina. This should be reported.

Comment 7: If it is true that 80 Mb of the genome has no Illumina coverage, then the error correction would not have been applied as in the rest of the genome. This would result in an overestimate of polymorphisms for those parts of the genome. This could result in several artifacts,

including failure to remove alternative haplotypes with the very stringent filtering that was applied (98% identity). I suspect that this genome assembly still contains alternative haplotypes, and that accounts for the discrepancy in kmer and flow cytometry genome size estimates.

Response to comments 6 and 7: We also considered the genome size of our assembly might be a serious problem. We therefore conducted PCR-free Illumina sequencing that is not affected by PCR biases to obtain a more accurate estimate of genome size. This also yielded an estimate of ~ 400 Mb, as before, so we considered the flow cytometry estimation, which had a wide range (431-539 Mb), could be erroneous. We therefore repeated the flow cytometry analysis again using another machine and obtained a genome size estimate of ~421±8 Mb based on 17 replicates (supplementary table 1), which is closer to the *k*-mer based estimate.

Following this, we conducted Oxford Nanopore Technology (ONT) sequencing to obtain longer reads to try to solve the problem of alternative haplotypes and redundancy in our genome assembly. A new version of the Cushion willow genome was obtained based on ONT long reads and polished by PacBio long reads and Illumina short reads. The contiguity of the updated genome was significantly enhanced, with a size of 339.6 Mb, containing 78 contigs, with contig N50 = 9.52 Mb and scafford N50 = 17.92 Mb. We found there were large amounts of high frequency (> 1×10^5) *k*-mers in the PCR-free reads (1705 kinds of 17-*k*-mers sum to ~1.626 Gb), which might originate from highly repetitive regions such as centromeres and telomeres. These high frequency *k*-mers amount to ~47.8 Mb, meaning that the genome size is reduced to ~350 Mb if these high frequency *k*-mers are excluded. It is very difficult to successfully assemble the centromeres and telomers based on current sequence data, because the sequence read length is still not long enough, and since in subsequent analysis repeat regions are masked. Consequently, we did not attempt to assemble these highly repetitive sequences.

The reasons for the large difference in genome size between the updated genome assemby and the previous one might be: (1), when the there are bubbles in the assembly graph caused by heterozygosity, HGAP/CANU tend to assembly all allelic haplotigs, while SMATRTdenovo/CANU tend to combine allelic hplotigs into one; (2), long reads generate by ONT can successfully sequencing high teterozygous regions more effectively and therefore combine allelic haplotigs more effective.

When we mapped all types of sequence reads (ONT, PacBio and Illumina) back to the updated genome assembly, 98.49% of Illumina short reads were mapped successfully back to the genome assembly, and only 1.18% of the genome assembly was not covered by Illumina short read (supplementary Table 6), implying that the current assembly covered most unique genomic regions and high accuracy of the genome assembly. We report this result in the revised manuscript.

Comment 8: The dot plot in Fig. S7 is quite interesting, and seems to show some large-scale duplications in cushion willow, and an apparent lack of the large discrepancy found between Chr1

and Chr16 in S. suchowensis. However, it is very hard to tell due to the high background (the vertical lines). Please produce a clearer version of this figure and discuss the genome comparisons more thoroughly. It would be quite interesting if cushion willow also has the Chr1 fusion that is present in Populus but absent in S. suchowensis and S. purpurea.

Response: Sorry for the unclear figure. We present a clear version of dotplot (Fg. 2c, Supplementary Fig. 8) based on the updated genome assembly. We compared not only Cushion willow with *Populus trichocarpa*, but also *Populus deltodies* and *Salix purpurea*. We show that genome synteny within *Salix* and *Populus* species are both high, but some large rearrangements were evident in *Salix* chromosomes 4, 15 and 19. The fission and fushion events between choromosomes 1 and 16 were also observed (Fig 3d, Supplementary Fig. 8) when comparing *Salix* species with *Populus* species. We therefore added these results to the revised manuscript.

Minor Issues

Comment 9: Lines 112-114. The rationale for concluding that the genome is highly repetitive based on the heterozygosity rate of 0.2% is not clear. Are the authors concluding that this level of polymorphism is too low to account for the second peak in the kmer distribution? This has not been demonstrated, and in my experience this assumption would be untrue. In any case, the rationale for this conclusion needs to be more clearly stated.

Response: We agree that the conclusion is not appropriate and therefore deleted it from the revised manuscript. We estimated heterozygosity rate based on the updated genome assembly, it is 0.71%, i.e. higher than the previously version.

Comment 10: Lines 137-138. The manuscript states that 97.85% of genes were annotated based on sequence similarity to annotated proteins in public databases. This is likely to be misleading, since many of the "annotations" in public databases are probably "gene of unknown function" or something similar. I think the authors mean that 97.85% of the predicted proteins matched a predicted protein in public databases. "Gene of Unknown Function" is not an annotation. The functional annotations should be quantified more precisely, or the statement should be clarified.

Response: Thanks for this suggestion, we therefore rephrased this as: “Most of the predicted proteins (97.4%) matched a predicted protein in public databases (Supplementary Table 9)”.

Comment 11: Lines 160-165. The analysis reported here is not a robust demonstration of monophyly of Salix and Populus, which is not really in doubt in any case. I recommend deleting this paragraph. However, I agree that the phylogeny is a useful framework for presenting the gene family results, so Fig. 2b is helpful.

Response: It’s true that the monophyly of Salicaceae as well as *Salix* and *Populus* is solid, as proved by previous studies including ours, so we deleted this content from the revised manuscript.

Reviewer #2 (Remarks to the Author):

Comments to the authors

The authors report a high-quality, chromosome-scale genome of Cushion willow (Salix brachista), which they use as a reference when resequencing 77 individuals from 14 populations across the species' distribution range (incl. sites in the Hengduan Mountains region and neighbouring regions), to characterize the species´ genetic diversity, population structure, demographic history, and footprints of adaptive evolution to the subnival environment. The authors interpret their results in the context of explaining high plant species richness within the HDM.

I would like to express my great appreciation to the authors for this extensive study, which aims at bridging the gap between genomics, population genetics/genomics, and species richness patterns, at the backbone of a geologically active mountainous region. I found the manuscript very interesting to read, but also came across several issues that will deserve some careful attention. A very recent paper (Muellner-Riehl, 2019), dealing with the geographic region which is also the focus of this manuscript, tackles many of these issues and provides suggestions for future research avenues, some of which ideally could be taken up by this study.

In the following, where appropriate, I will refer to the line numbers as given in the merged pdf file. I hope that my comments will be helpful for the authors.

Response: Thanks very much for your positive comments to our study and highlighting the recent paper by Muellner-Riehl .

Major issues

Comment 1: The authors interpret their population-level, infra-specific results in the context of explaining high plant species richness within the HDM. The connection between the two (which would be speciation) is currently not convincingly presented in the manuscript. There have been some recent attempts to view speciation and the origination of high levels of species richness in the same context. For example, the recently presented Mixing-Isolation-Mixing (MIM) model of speciation (He et al. 2018; compare comments by Abbott, 2018, and Rieseberg, 2018), supports that speciation may involve repeated cycles of admixture, followed by geographic isolation until complete isolation is finally achieved. By permitting intermittent gene flow during speciation, MIM cycles can potentially generate species at higher rates than just by geographic isolation, which in turn means that speciation and the origination of high biodiversity levels in a region may be viewed in the same context framework (Heet al., 2018). Translated to the Hengduan Mountains region and Salix, one would postulate that the climatic fluctuations of the Quaternary would have forced populations to shift their distribution ranges (thus being repeatedly connected and

disconnected again), possibly with a species-pump acting. See Muellner-Riehl (2019) for more details.

Response: Thanks for these valuable suggestions and comments. First, we want to emphasize that we have updated the Cushion willow genome assembly (see above) and performed all analyses again, yielding results similar to those reported previously.

Our results indicate that *S. brachista* split from one of its close relatives around 13 Ma, and subsequently underwent intraspecific divergence more recently (around 0.18-0.42 Ma), i.e. after the intense uplift of the HDM. In the revised ms, we propose that our results are consistent with the hypothesis that diverse isolated heterogeneous habitats on sky islands, together with climatic fluctuations that have possibly caused recurrent cycles of admixture and isolation between some populations (MIM cycles), were important drivers of intra-specific population diversification in Cushion willow, and by extension, other alpine species with similar distributions and histories in the HDM. We further speculate from our results that geographic isolation and environmental variation associated with elevation resulting from the uplift of THR, together with the effects of Quaternary climate fluctuation, have contributed to the high species richness observed in the HDM by driving intraspecific population divergence, a common precursor of speciation.

Comment 2: The authors argue for uplift-driven diversification (as many other authors have done before) in an ad hoc manner, but they don't test this. What could be alternative explanations for the patterns observed? How could different scenarios be compared and tested? What is the most likely explanation? (see Muellner-Riehl 2019 for some suggestions). Connected to the issue raised above, the relation of population-level, infra-specific differentiation and (species-level) diversification is not apparent.

Response: As we have mentioned in response to comment 1, the split time of six population pairs ranges between 0.18 and 0.42 Ma, which is relatively young and after the intense uplift of the HDM. So we propose the main drivers of intraspecific diversification include geographical isolation across sky island habitats and possibly MIM cycles driven by Quaternary climate fluctuations.

Comment 3: Caution needs to be taken concerning the naming and delimitation of the mountain regions. The Qinghai-Tibetan Plateau (QTP) proper, the Himalayas, and the Hengduan Mountains region have to be viewed separately, as they have quite different geological (uplift) histories, different orientation, were/are differently impacted by climatic conditions (e.g., monsoonal influence; glaciation), and have different mountain morphology, the latter resulting in contrasting scenarios for population connectivity during either cooling or warming phases through time. See Muellner-Riehl (2019) for more details.

Response: In the revised manuscript, we use the term "Tibeto-Himalayan" (THR) instead of "Qinghai-Tibetan Plateau" (QTP), and delimit the three separate mountain regions of the THR in

accordance with Mullner-Riehl (2019), i.e., QTP, the Himalayas, and the Hengduan Mountains (HDM).

Comment 4: The authors investigated GO enrichment for pathways related to DNA damage and repair for Salix brachista, and looked for footprints of adaptive evolution in the genome. It would be interesting to know how Salix brachista does compare to the other Salix species investigated in this study.

Response: Thanks for this valuable suggestion. We now point out that GO/KEGG enrichment analyses for two other *Salix* species with genome data (*Salix purpurea, S. suchowensis*) revealed no significant enriched GO/KEGG terms relating to alpine adaptation (i.e., DNA repair, response to hypoxia) (supplementary table 10-13), in contrast to what was found for Cushion willow.

Comment 5: In several instances, the authors make claims about a connection between adaptation and "climate change during QTP uplift" and "the onset of harsh subnival environmental conditions". This is problematic for two reasons: First, the QTP uplift predates the Hengduan Mountains uplift (see my previous comment about correct naming and delimitation of mountains regions of the Tibeto-Himalayan Region (THR); see also Muellner-Riehl, 2019), and second, it is not clear what kind of "climate change" the authors are referring to. This is one of several examples where care needs to be taken for a proper use of terminology and handling of abiotic factors.

Response: In the revised manuscript, we estimated the divergence time of Cushion willow from one of its closest relatives. Crown age estimation of Cushion willow was deleted (Reviewer #3 pointed out it is not appropriate to estimate divergence time of a species using individuals of it in the context of phlygenetic tree construction). We also estimated the split times of Cushion willow populations as suggested by Reviewer #3 using fastisimcoal2. Our results indicate that *S. brachista* split from one of its closest relatives around 13 Ma, in accordance with the intense uplift of the QTP and Himalayas, whereas populations of Cushion willow diverged from each other much more recently (around 0.18-0.42 Ma), i.e. after the intense uplift of HDM. Therefore, the origin of *S. brachista* might be associated with tectonic events, while its population divergence was likely driven by habitat isolation (across sky islands) and possibly MIM cycles as mentioned previously.

Comment 6: Of a somewhat similar problematic is the discussion of population bottlenecks with regard to glaciations. Information needs to be provided about the regional extent of these glaciations (into the species distribution range), how these glaciations would have possibly impacted vegetation cover in the HDM, either directly by glacial cover, or a drop in temperature. The age for the LGM given is too young, and it is known that the maximum glacial extent in the THR was not at the time of the LGM, but pre-dated the LGM (see Muellner-Riehl 2019 and

references therein for details), thus rendering the time of the global LGM as not specifically relevant for the THR.

Response: We have re-analyzed the demographic history of Cushion willow based on the genetic groups identified by genetic structure analysis as suggested by Reviewer #3. We found multiple bottlenecks to occur for most of these groups, but their timing and number differed. Such variation in demographic history between these groups is not unexpected given that glaciation of the THR region was highly variable, with strong microclimatic variation present within individual mountain ranges. Each bottleneck event was usually followed by a population expansion, reflecting major climatic oscillations during the Pleistocene. Since bottleneck events are population or mountain specific and do not accord with the five distinct glaciation periods in the THR, we deleted these glaciation periods from figures and present a $\delta^{18}O$ curve (which is an indicator of global ice volume and temperature) instead (Fig. 3f, Supplementary Fig. 10).

Comment 7: More details should be given concerning the molecular dating analysis (use of priors, age of fossils, setting of constraints).

Response: We have added these details in Methods. In the new version of the ms, we estimate the divergence time of Cushion willow from another dwarf willow in THR (*Salix souliei,* also placed in section *Lindleyanae*). Since the earliest reliable fossil of Salicaceae is in the early Middle Eocene, about 48 Ma, and another study estimated the divergence time of *Populus* and *Salix* lineage as ~52 Ma, we calibrated the root age as 48-52 Ma.

Comment 7: Concerning environmental heterogeneity between high and lower elevation sites, more information should be given about the rationale behind using the climatic parameters finally chosen, and not other abiotic parameters, like soil, or other measures of geodiversity.

Response: *Salix* is one of the main groups of woody plants in the North Temperate Zone. Previous studies suggest that habitat heterogeneity and contemporary climate may primarily act as environment filtering forces in shaping individual *Salix* distributions and influence species composition by filtering species according to their different climate preferences (Wang et al, 2017, Scientific Reports 7: 42038; Blonder B, et al., 2016, Ecology 96: 972-985). We therefore chose the parameters of climate and habitat heterogeneity according to the above references to test the environmental differences between high and lower elevation sites.

Wang Q, et al. Historical factors shaped species diversity and composition of *Salix* in eastern Asia. Scientific Reports 7, 42038 (2017).

Blonder B, et al. Linking environmental filtering and disequilibrium to biogeography with a community climate framework. Ecology 96, 972-985 (2016).

Minor issues (and details)

Abstract:

Comment 8: 29-31: “The Hengduan Mountains (HDM) is a biodiversity hotspot exhibiting exceptional plant diversity, particularly for alpine plants. However, the origins of this high alpine plant richness remain largely unknown”. This is not entirely true (N-S orientation of HDM enabling easier migration during climatic changes, less glaciation impact, monsoonal influence, steep gradients with shorter migration distance during climatic fluctuations in the Quaternary, etc.). See Muellner-Riehl 2019 and references therein. Apply necessary text changes in all other relevant parts of the ms as well.

Response: We agree with this and re-wrote as “The Hengduan Mountains (HDM) biodiversity hotspot exhibits exceptional alpine plant diversity”.

Comment 9: 35: genome “sequence”? re-phrase

Response: We deleted “sequence” here.

Comment 10: 38: “topographic features of the HDM are associated with…“ Which features exactly? Be more precise. Testable?

Response: We rephrased this as: “shaped by a landscape of isolated sky island-like habitats in addition to environmental heterogeneity across elevational gradients”, please see the Abstract section for detail. See also our response to comment 1.

Comment 11: 40: use of “sky islands” here and elsewhere - Please provide information which definition you apply for the term “sky islands”. Not all readers will be familiar with this term, and it is applied in differently stricty ways of its definition.

Response: We use Heald’s concept of “sky island”, we added the corresponding reference in the revised manuscript: Heald, W. Sky islands of Arizona. Nat Hist 60 56–63 (1951).

Comment 12: 41: “heterogeneous environments across different elevations” be more precise (heterogeneity measured by? Which elevations?).

Response: We rephrased as “....environmental heterogeneity across elevational gradients”. Environmental heterogeneity refers to heterogeneous environments between high and lower elevation sites. We have described the method to measure this heterogeneity in “Environment heterogeneity between high and lower elevation sites occupied by Cushion willow” (Method section).

Comment 13: 44: Use of “subnival” versus “alpine” – please check for consistency of the use of these terms throughout the ms

Response: Cushion willow is a typic alpine plant of THR, mainly distributed in subnival zones and seldom in lower elevation area (~3000 m). We checked throughout the manuscript to

make the use of “subnival” and “alpine” appropriate depend on specific context.

Comment 14: 47-50: “These findings indicate that the HDM served as an evolutionary cradle and driver of diversification of alpine species during its history, which contributed to the very high level of plant diversity characterizing this region today.“ Strictly speaking, this was not investigated here in the study, the latter which focused on a single species. See my comment under “major issues“.

Response: We re-wrote this part emphasizing intraspecific divergence, often a first step in speciation: “These factors, together with population size fluctuations indicated to have occurred during the Quaternary, are likely important drivers of intraspecific divergence within Cushion willow and possibly other alpine plants similarly distributed in the HDM. Because intraspecific divergence is often a first step in speciation, we propose that such factors were important contributors to the generation of high alpine species diversity in the HDM.”

Introduction:

Comment 15: 53: “Hengduan Mountains (HDM) biodiversity hotspot“ This is not the official name of the biodiversity hotspot. It is called “Mountains of Southwest China”, and includes the Hengduan Shan.

Response: We prefer to use “Hengduan Mountains” instead of “Mountains of Southwest China”, because it is widely used in China and in most references including some recent publications listed below:

Favre, A. et al. The role of the uplift of the Qinghai-Tibetan Plateau for the evolution of Tibetan biotas. Biol Rev Camb Philos Soc 90, 236-53 (2015).

Boufford, D.E. Biodiversity Hotspot: China's Hengduan Mountains. Arnoldia 72, 24-35 (2014).

Xing, Y.W. & Ree, R.H. Uplift-driven diversification in the Hengduan Mountains, a temperate biodiversity hotspot. Proceedings of the National Academy of Sciences of the United States of America 114, E3444-E3451 (2017).

Sun, H., Zhang, J., Deng, T. & Boufford, D.E. Origins and evolution of plant diversity in the Hengduan Mountains, China. Plant Diversity 39, 161-166 (2017).

Comment 16: 57: provide citation for species number

Response: Done.

Comment 17: 58: alpine and subnival – see my comment before

Response: We have carefully checked the use of “alpine” and “subnival” throughout the manuscript.

Comment 18: 61-63: Be more specific about the hypothesis. The hypothesis also includes a temporal component and climatic fluctuations, see the "mountain-geobiodiversity hypothesis" by Mosbrugger et al. 2018 and an updated version in Muellner-Riehl 2019.

Response: We have changed the text to make the hypothesis more specific, please see the revised manuscript (line 58-63).

Comment 19: 65-66: Important reference missing here, where potential drivers were actually tested:

Ebersbach, J., Muellner-Riehl, A.N., Favre, A., Paule, J., Winterfeld, G. & Schnitzler, J. (2018). Driving forces behind evolutionary radiations: Saxifraga sect. Ciliatae in the region of the Qinghai-Tibet Plateau. Botanical Journal of the Linnean Society 186, 304-320.

Ebersbach, J., Schnitzler, J., Favre, A & Muellner-Riehl, A.N. 2017. Evolutionary radiations of the species-rich mountain genus Saxifraga L. BMC Evolutionary Biology 17, 119.

Response: We have added these references to the manuscript.

Comment 20: 67: "important questions" – please state which ones.

Response: We mean here studies based on few DNA markers have limited power for biogeographical and phylogeographical analysis. We think this sentence is not needed and deleted it.

Comment 21: 68-73: Population genomics analysis and "causes of high alpine plant diversity within the HDM" are two pairs of shoes. See my comment under major issues.

Response: We agree this, and we rephrased this as : "interpret our results in the context of explaining rapid intraspecific divergence leading to high plant species richness within the HDM".

Comment 22: 74-75: Provide information about how many species of Salix do occur in the HDM. What are the closest relatives of S. brachista and what is known about hybridization?

Response: There are ~152 Salix species occur in HDM, we mentioned this in Introduction. *S. brachista* belong to *Salix* subg. *Chamaetia* sect. *Lindleyana*, all members (~21 species) of this section are dwarf willows in subnival zone of THR, but we don't know which species is its closest relatives, because our previous studies and many other studies failed to resolve the relationship inside the largest clade consist of *Salix* subgenus *Chamaetia* and *Vetrix* (*Chamaetia*-*Vetrix* clade, account for ~73% species of *Salix*) revealed by phylogenetic analysis, this is one of the main reason that we sequenced Cushion willow genome, we hope this would facilitated phylogenomic studies to unravel the relationships and evolution of HDM willows in the future (we think evolutionary radiation is quite possibly involved). Hybridization is considered to be frequent in willows, in sect. *Lindleyana*, *S. brachista* has distinct and stable morphology traits, and we did not find intermediate individuals between it and other related species, that's why we chose this species

for whole genome sequencing.

Results and discussion:

Comment 23: 165: You may want to consult Peter Stevens´s Angiosperm Phylogeny Website (go to "Salicaceae") at http://www.mobot.org/MOBOT/Research/APweb/welcome.html to take a look at other studies which have been conducted on Salicaceae phylogenetics and molecular clock dating.

Response: The Salicaceae family have reliable fossils with flowers and/or fruits (Boucher et al., 2003, American Journal of Botany 90:1389-1399; Manchester et al, 2006, International Journal of Plant Science), so we use calibration based on these fossil records in molecular dating, this method was also used in divergence time estimation in a former study of ours (Wu et al., 2015, BMC Evolutionary Biology 2015:15).

Comment 24: 179-198: How do patterns compare to other species of Salix included in this study?

Response: We have made comparisons with two other *Salix* species - please see our response to comment 4.

Comment 25: 207: alpine – see my previous comment about the use of "subnival" and "alpine"

Response: We have carefully checked the use of "alpine" and "subnival" throughout the manuscript.

Comment 26: 208: provide information and citation for this high temperature

Response: In analysis based on our updated genome assembly, we did not find significant GO/KEGG enriched terms related to heat and cold response, so we deleted this paragraph.

Comment 27: 220: WGD events – at this stage is is unclear to the reader where (which specimen/species) this was investigated

Response: The WGD analysis was based on the whole genome sequenced individual of Salicaceae species, we used these genome assembles to identify orthologous genes shared by these Salicaceae species.

Comment 28: 224-225: unclear to which level this refers to (ancestor of ?)

Response: We refer to Cushion willow here, we have corrected this in the revised manuscript.

Comment 29: 231: genetic structures – which ones?

Response: We mean genetic structure between *Populus* and *Salix*, we have changed this in

the revised manuscript.

Comment 30: 234: citation 28 missing here

Response: Corrected.

Comment 31: 238: recent burst of further gene duplication – what is exactly meant by this?

Response: We meant that there may be a recent WGD event in Cushion willow, but this peak is weak, and WGD analysis based on the updated genome assembly did not show the peak. The weak peak likely resulted from redundancy in the previous genome assembly. We have modified this paragraph based on the results of the new WGD analysis .

Comment 32: 240-241: "footprints of adaptive evolution in the Cushion willow genome.“ – anything known about the other Salix species included in this study? How do they compare in terms of ecology/biology?

Response: Cushion willow is a typical subnival species in THR which encounters harsh alpine environmental conditions. As we mentioned in response to comment 4, we did not find the GO/KEGG enriched terms (present in Cushion willow) that might be related with alpine adaptation (i.e., DNA repair, response to hypoxia) in the other two *Salix* species (*S. purpurea, S. suchowensis*) examined. The most distinctive morphological feature of subnival (and also high latitude willows such as those in arctic tundra region) compared with plain willow is their dwarf/cushion shaped habit.

Comment 33: 243-244: „may have facilitated species-specific adaptations driven by climate change during QTP uplift and the onset of harsh subnival environmental conditions" – what kind of climate change are you referring to? (attention: uplift of the QTP and adjacent mts. was not the cause of the origination of the monsoon system, and the QTP had an average elevation of 3-4 km already since the Eocene; see reviews by Favre et al. 2015, Renner et al., 2016, Spicer, 2017; Botsyun et al., 2019).

Favre, A., Päckert, M., Pauls, S.U., Jähnig, S.C., Uhl, D., Michalak, I., et al. (2015). The role of the uplift of the Qinghai-Tibetan Plateau for the evolution of Tibetan biotas. Biol. Rev. Camb. Philos. Soc. 90, 236–53. doi:10.1111/brv.12107.

Renner, S.S. (2016). Available data point to a 4-km-high Tibetan Plateau by 40 Ma, but 100 molecular-clock papers have linked supposed recent uplift to young node ages. J. Biogeogr. 43, 1479–1487.

Spicer, R. A. (2017). Tibet, the Himalaya, Asian monsoons and biodiversity - In what ways are they related? Plant Div. 39, 233–244.

Botsyun et al. (2019). Revised paleoaltimetry data show low Tibetan Plateau elevation during the Eocene. Science Vol. 363, Issue 6430, eaaq1436. DOI: 10.1126/science.aaq1436

Response: We agree that it is arbitrary to state that the WGD of Cushion willow is driven by climate change during QTP uplift and the onset of harsh subnival environmental conditions. But in the WGD analysis based on the updated genome assembly, this Cushion willow specific WGD event was not found anymore (most likely attributed to redundancy in the previous genome assembly). Therefore we deleted this sentence.

Comment 34: 278: monsoon system in the QTP – please specify. The QTP proper and the western Himalayas are almost entirely beyond the reach of the summer monsoon.

Response: Thanks for this reminder, we specified it as "HDM and the eastern Himalayas".

Comment 35: 292: generation time of three years – seems rather low given the harsh conditions under which these plants grow. Please provide citation, upon which literature (or personal observation) this is based?

Response: We use the generation time of three years based on the reference of Smart et al.(Genetic improvement of shrub willow (Salix spp.) crops for bioenergy and environmental applications in the United States, (2005)). We have cited this in the Method but missing here, we then added it here. There is no research on generation time of willows in harsh conditions, so we think it's better to use a generation time based on a published reference.

Comment 36: 295: environmental change in the QTP – see my comments under major issues. The populations investigated don´t occur in the QTP proper. Please check - there is no Fig. 3f (should this be 3e?).

Response: We have re-analyzed the demographic history for genetic groups identified by Structure analysis of Cushion willow instead of for the whole species. We found population-specific demographic history, with timing and number of bottlenecks and expansions fluctuating through time. We re-wrote this part and addressed the issue. Fig. 3f should have been fig. 3e. However, in the new version it is Fig. 3f, due to a modification of this figure..

Comment 37: 303-304: LGM age given is too young. If this refers to the global LGM (terrestrial), see Hughes, P.D., and Gibbard, P.L. (2015) A stratigraphical basis for the Last Glacial Maximum (LGM), Quat. Int. 383, 174–185. doi.org/10.1016/j.quaint.2014.06.006.

Response: In the revised ms we use the interval of 0.0275-0.0233 Ma for LGM.

Comment 38: 306-307: adapted to environmental change – please re-phrase. Species are not adapted to change, but to certain conditions. Uplift of the HDM – which uplift are you referring to? Specify.

Response: We rephrased it as "adapted to subnival conditions".

Comment 39: 307-311: Needs re-phrasing, see Muellner-Riehl 2019. 10 is not a glaciation reference (see Muellner-Riehl 2019 for glaciation information and literature). Also, it is not clear

how this would fit with Fig. 3e.

Response: We have rephrased here according to our new demographic analysis results (please see the revised manuscript for detail). We changed the incorrect reference to Owen et al (2014).

Comment 40: 317: robust clade – definition of „robust“?

Response: We mean a clade with 100% bootstrap value. But since Reviewer #3 advised that it is not acceptable to infer relationships of individuals or populations of a species using other species as outgroup in a model-based phylogenetic tree, we omitted this ML tree in the revised manuscript.

Comment 41: 326: How do the age estimates of this study compare with previous age estimates by other authors?

Response: There are no convincing divergence time estimates for inner clades of Salix subgenus Chamaetia-Vetrix clade. Support value were low in previous studies based on few DNA markers. Since it is not appropriate to conduct divergence time using different individuals of a species, we estimated the divergence time of Cushion willow from one of its closest relatives (Salix souliei) using 390 single copy genes.

Comment 42: 328-329: "This overlaps with the confidence interval for the divergence time of Cushion willow, suggesting that the species origin was associated with tectonic changes in the HDM.“ This is an ad hoc assumption, do you have real evidence for this? How could this be tested, what are alternative explanations? See Muellner-Riehl 2019 ("clearly defined phylogeographic hypotheses may be tested rigorously, instead of applying explanations that fit the observed results ad hoc (i.e., after analysis of the data), as has been done in the past.").

Response: As mentioned in above responses, we now estimated the divergence time of *S. brachista* and *S. souliei*, which is ~13 Ma, and the split times of six populations pairs using fastsimcoal2. The crown age of *S. brachista* is currently impossible to estimate. We have revised the discussion of the origin of Cushion willow and intraspecific divergence in the new version of the ms (see "Temporal origins and onset of intraspecific diversification" section).

Comment 43: 336-338: See some of my previous comments about the connection between population divergence within species and species richness. As presented now, the direct link (if it does exist at all – not proven yet for this region, but some studies pointing towards this) is not convincingly presented. The study by Xing and Ree investigates species richness / is based on species-level and above, not popuation divergence, but they provide interesting thoughts concerning the HDM. See also Muellner-Riehl 2019.

Response: Since we cannot estimate the crown age of Cushion willow in the revised manuscript (please see above responses for the reason), we have deleted these sentences in the

revised manuscript.

Comment 44: 353: some unique terms – meaning unclear

Response: Because there are no significantly enriched GO/KEGG terms for genes in selective sweep region we have deleted this sentence.

Comment 45: 355: Where appropriate in the text, it would be interesting to learn more about the potentially different impact of competition (from other co-occurring plant species) that the more lowland versus subnival populations of S. brachista might be subjected to, also given the fact that the selective pressure observed seems weaker in high elevation sites.

Response: Thanks for this valuable suggestion. *S. brachista* only grows in open habitats: habitats in subnival scree slope are completely open (Fig. 1d), while in low elevation localities around 3000 m, *S. brachista* is only found on open river banks and never occurs in forest near the river (Fig. 1c). Therefore, it seems that competition of lowland and subnival populations of Cushion willow with co-occuring plant species are unlikely to be different.

Comment 46: 374 and following: The situation described is the one of nowadays, formerly it was colder, populations moved down and were better connected. See Muellner-Riehl 2019.

Response: We have rephrased the conclusion part and addressed this issue, please see the Conclusion section for detail in the revised manuscript.

Comment 47: 384: elevational gradients

Response: We have changed “elevation”to “elevational”.

Comment 48: 386: diverse heterogeneous habitats – meaning unclear, specify

Response: We have rephrased this as “Our results are consistent with the hypothesis that diverse isolated heterogeneous habitats on sky islands”.

Comment 49: 388: and following: Again, please see the problematic with this statement as outlined previously. It is not merely populations being uplifted with the mountain as it rises, it is climatic fluctuations making plant populations move and being separated or connected at various times on and across mountains.

Response: We re-wrote these sentences, pointing out not only fragmentation and isolation, but also Quaternary climate fluctuation may result in intraspecific diversification: “We therefore speculate from our results that geographic isolation and environmental variation associated with elevation resulting from the uplift of THR, in combination with the effects of Quaternary climate fluctuation, have contributed to the high species richness observed in the HDM by driving intraspecific population divergence, a precursor of speciation.”

Methods:

Comment 50: 393: Please provide voucher information on the specimen investigated (sequenced), collector and collector number, date, location, deposited at which herbarium, etc.

Response: We provide the voucher information in Supplementary Table 14.

Comment 51: 442: Here and elsewhere in the ms, please take care of properly setting spaces

Response: Sorry for this, we have check the ms carefully for space setting.

Comment 52: 455: artefact (though artifact also possible)

Response: We mean contaminated (artefact) contig here, since we did not find contaminated contig in the updated genome assembly, we delete "artefact".

Comment 53: 518: with the GTRGAMMA

Response: Sorry for this grammatical error, since the positive selection did not find significantly enriched GO/KEGG terms, we deleted this part as suggested by reviewer #3.

Comment 54: 519: Here and elsewhere in the ms, please take care of using hyphens in compound words

Response: Sorry for this, we have checked the throughout the manuscript for hyphens in compound words and made corrections

Comment 55: 26: generated a maximum; which evolutionary model was used?

Response: It is the GTRGAMM model, we have added it.

Comment 56: Comment 54: 549-550: This sentence is not clear, please clarify and re-phrase. Certainly, you would have first run the actual NJ analysis, and then the independent boostrap analysis (the latter which is based not on the original data matrix, but the pseudo-replicates).

Response: We rephrased it as : "...clade support was calculated with1000 bootstraps".

Comment 57: 560 and following: Something is wrong with this sentence; phrasing and meaning of sentence unclear. Please provide the age of the fossils, based on the original primary literature.

Response: Both of the fossils cited were from the early Middle Eocene (~48 Ma) based on the original primary literature.

Comment 58: 564: Unclear – was a maximum root age set? Was a range of between 48 and 52 tested? Please specify in the text the rationale behind the setting of temporal constraints, and provide more detailed information about the priors.

Response: Yes, we used a range here. As indicated in the manual of MCMCtree, if the

minimum bound is based on good fossil data, the true time of divergence may be close to the minimum bound. And since the oldest reliable fossil age is early Middle Eocene around 48 Ma, and Dai et al (2014) estimated the divergence time of Populus and Salix as 52 Ma, we think it is reasonable to calibrate the root age to 48~52 Ma in the MCMCtree analysis. We also used this similar calibration (root age calibrated as around 48 Ma) in one of our former papers (Wu J, Nyman T, Wang D-C, Argus GW, Yang Y-P, Chen J-H. Phylogeny of Salix subgenus Salix s.l. (Salicaceae): delimitation, biogeography, and reticulate evolution. BMC Evol Biol 15, 31 (2015)).

Comment 59: 602 and following: Provide information about the rationale behind considering only climatic parameters (and the ones you have chosen). What about soil or other geodiversity parameters? What would be the potentially most important determinants of occurrence for S. brachista?

Response: Please see the explanation of how we chose parameters in response to comment 7, and that previous studies indicate the distribution of *Salix* is mainly affected by climate factors and environmental heterogeneity.

Comment 60: 609: Shannon, Simpson

Response: We have capitalized the first letter for these words in the revised ms.

References:

Comment 61: Need to be carefully checked for typos (diacritics in author names), formatting, use of upper and lower case, etc. throughout.

Response: We have checked the references section for these issues carefully and made corresponding corrections.

Figure captions:

Comment 62: Figure 1: a) and b) flowers, not flower; m a.s.l.? 1f) Scheme or schematic? (both terms possible) This picture is very simplified, and does not consider mountain morphology (which is different for HDM versus Himalayas or QTP). You may consider using a real cross-section (compare Muellner-Riehl 2019) and then simplify it (smoothing the lines). Be aware that in the Himalayas/QTP, area size for alpine plants will increase at higher elevation.

Response: Thanks, we have made corresponding corrections as suggested. We also have changed to a map which shows mountain morphology.

Comment 63: Figure 2: c) Please check whether the legend in the right-hand upper corner is correct. Insert “events” after “(WGD)”.

Response: Since we only found the common WGD event in Salicaceae, we do not show the Ks distribution in Fig. 2, and only present it in Supplement Fig. 7 without this legend.

Comment 64: Figure 3: “tines” correct? Last Glacial Maximum. Concerning timing of the global terrestrial LGM, see my previous comments (for 303-304) and Hughes and Gibbard 2015.

Response: Sorry for the typo, it is “time”. Since bottleneck events are population or mountain specific and do not accord with the five distinct glaciation periods in THR, we deleted these glaciation periods from figures and present a $\delta^{18}O$ curve (which is an indicator of global ice volume and temperature) instead (Fig. 3f, Supplementary Fig. 10). Related content in the manuscript was deleted.

Figures:

Comment 65: Improve readability of fonts used (e.g., Fig. 1e, river and mountain names)

Response: We changed to a map with clear mountain terrain. The mountains are hard to distinguish, so we deleted their names from the map.

Final:

Comment 66: - Carefully read, check and correct for clarity and logic of English language and use of scientific terms throughout the manuscript.

Response: We have checked throughout the manuscript for the issues listed.

Comment 67: - You may want to read (and cite) the following publication for comparison with your study:

Cai, L. [et al. 2018], Xi, Z., Amorim, A. M., Sugumaran, M., Rest, J. S., Liu, L., & Davis, C. C. 2018. Widespread ancient whole genome duplications in Malpighiales coincide with Eocene global climatic upheaval.https://nph.onlinelibrary.wiley.com/doi/epdf/10.1111/nph.15357

Response: Thank for this suggestion, we have read and compared this study with ours, it helped us in revising the manuscript.

Alexandra Muellner-Riehl

Reviewer #3 (Remarks to the Author):

This is a very interesting and impressive study in which the authors conduct an ambitious population genomic analysis for a plant species native to an understudied biodiversity hotspot. The authors generated the first reference genome for Salix brachista and it important to note that they have been successful in scaffolding their reference genome into what appear to be the 19 chromosome of this species. To do so they have used state-of.the-art methodology such combining long and short reads for the assembly and HiC for the scaffolding. They also propose a very interesting set of 77 re-sequenced accessions sampled in localities with very different environmental and climatic conditions.

I would like to felicitate the authors for having undertaken this comprehensive analysis of an interesting non-model plant species. I am convinced that these new genomic resources will be of great interest to the community. However, I have been disappointed by a series of imprecisions, inaccuracies through my reading of the analyses of this new data. But I am convinced that if the authors can address the issues listed in my review to meet the standard of the field, they could finalize a great contribution to the plant population genomic literature.

Response: Thanks very much for your comments on our study. We are sorry for the imprecisions and inaccuracies you encountered. We have addressed these issues when revising the ms.

Genome assembly.

Comment 1: 114: The authors state here that given the measured heterozygosity of 0.2%, the genome must be highly repetitive. It seems that this is deduced from the joint observation of the k-mer distribution and the observed heterozygosity but it is unclear from the text what justifies making this statement. Please describe the causal relations implied here.

Response: We agree that this statement is not convincing, so we delete this. Moreover, we updated the Cushion willow genome assembly based on Nanopore Oxford Technology (ONT) long reads, contiguity of the genome assembly was significantly enhanced, and we also found that the previous genome assembly included many small fragments of redundancy (please also see our response to comment 2). Heterozygosity rate based on the updated genome assembly is 0.71%, higher than previous estimated.

Comment 2: Figure 2c: The annotation of this figure suggest that each peak is associated to a whole-genome duplication. I wonder, however, what effect the duplication of a single or two chromosomes would have on such distributions. For example, could it be that the most recent peaks in the S. brachista distribution be caused by the duplication of the Hic groups 8/19 and 9/12 only? Is the term whole-genome duplication applied loosely to refer to a duplication of a substantial part of the genome? Because the locations of each pairs of paralogues used to calculate the Ks distribution are known, I assume it should be possible to find out which chromosomes are associated with each of the three peaks.

Response: The *S. brachista*-specific peak and the peak shared by *S. brachista* and *S. purpurea* were very weak (especially in *S. pupurea*) (reviewer #1 also addressed this issue). In the WGD analysis based on the updated *S. brachista* genome assembly, we only observed the common WGD event in Salicaceae, indicating that the additional peaks in *S. brachista* resulted from redundant sequence (Supplementary Fig. 7).

Phylogenetics

Comment 3: PAML analyses: the authors write in the M&Ms that they have estimated the

Ka/Ks ratio for each branch in the phylogeny (note that there is more than one ratio per branch in both models). How does the number of positively selected gene in S. brachita (1582, l. 175) compares to the distribution of equivalent numbers on all other branches. This is important to help the reader evaluate whether there is a particular signal of adaptation on the external branch leading to S. brachita or whether this very large proportion (1582/11288) could be an artefact of the statistical method employed here.

Response: We have redone the PSG analysis, we found many PSGs in *S. brachista* and *S. suchowensis*, and much less PSGs in other species and branches (please see the below table for detail). But like previous results, we did find significant enriched GO/KEGG terms in *S. brachista*, so as you suggested in comment 7, we remove this analysis and related contents from the revised manuscript.

**Lineage**	**PSGs** (FDR< 0.05)
Populus deltoides	567
Populus trichocarpa	389
Populus alba var. *pyramidalis*	645
Populus euphratica	116
Salix purpurea	369
Salix suchowensis	1194
Salix purpurea-Salix suchowensis	23
Salix brachista	1163
Ancestral *Salix*	73

Comment 4: l.180: significant GO enrichment: what set is enriched, PSG? The authors are not being precise here.

Response: We re-wrote this paragraph using new gene family enrichment (including expanded, fast evolve and species-specific gene families, and PSG analysis was removed as mentioned in response to comment 3) results based on the updated *S. brachista* genome assembly. See the revised manuscript for details (line 176-200).

Comment 5: l.182-183: in Sup Table 8 (line 252), the GO term "regulation of response to stimulus to DNA damage stimulus" is associated with a q-value of 0.96, that is non-significant. At line 180 the author claim this to be significant. Is there a mistake somewhere?

Comment 6: l.183-185: same here, all these terms are associated with q-values > 0.05 according to Sup. Table 9). It looks like the authors have based their assessment of significance on p-values not corrected for multiple testing.

Response to comments 5 and 6: In the revised manuscript, we only report enriched GO/KEGG terms with significant *p*-value (<0.05) corrected for multiple comparisons. We found some GO/KEGG terms related to DNA repair, please see the revised manuscript (line 176-200) and Supplementary Table 10-12 for details.

Comment 7: l.185-again, same problem for the PSG class. None of these GO terms seems to be significant at the 5% level after correcting for multiple testing. Furthermore, the classes reported by the authors in this paragraph are not even the one associated with lowest q-values (see Sup Table 8,9,10). Rather, it seems that the authors have cherry-picked these classes to be consistent with their a priori hypothesis of an adaptation to UV-radiation-induced damage. My conclusion for this paragraph is that either the authors manage to make sense of the list of significant GO terms (i.e. q-value < 0.05) or the whole paragraph should be removed. My personal recommendation is to remove this paragraph.

Response: Yes, there are no enriched GO/KEGG terms with a significant *q*-value (<0.05). However, the reason we discussed this was because we considered CodelML in PAML has its own statistical methods to determine positively selected genes, and the PSGs should themselves potentially indicate adaptive evolution. We have now removed this paragraph, as suggested (see also response to comment 3), because we detected no significantly enriched GO/KEGG terms for PSGs based on the updated *S. brachista* genome assembly.

Comment 8: l-189-192: this result has no relevance given my comments above.

Response: We found significantly (correct p-value < 0.05) enriched GO/KEGG terms related to DNA repair in *S. brachista* expanded, fast-evolve and species-specific gene families based on the updated genome assembly (Supplementary Table 10-12), e.g., "Base excision repair" (ko03410), "misfolded protein binding" (GO:0051787), "unfolded protein binding" (GO:0051082).

Comment 9: l.193-195: the GO term "anthocyanin accumulation […]" is not even present in Sup Table 8 and 9". No single sentence in this paragraph is backed by the results presented in the manuscript.

Response: This GO term was not in the table containing enrichment results, but the table contains a summary of GO terms (i.e., number of GO terms in gene families and PSG datasets) (Sup Table 11-13). Reviewer #1 considers it is not appropriate to use this method. In the revised manuscript, we therefore removed these tables. The discussion related to anthocyanin/flavonoids are based on significantly enriched GO/KEGG.

Comment 10: l. 240-241: This assertion should be reformulated to clearly indicate that it is based on results from PAML only. Given the very large number of genes identified as significant and the poor treatment of the GO analysis, I am not sure how much confidence can be given to this statement.

Comment 11: l-241->end of paragraph: no results are presented that show a significant enrichment of PSG genes in species-specific or species-specific-expanded gene families. The conclusions reached here a pure speculation.

Response to comments 10 and 11: As we mentioned in response to comment 3, we have removed positive analysis using PAML and related contents as suggested. Our results of the WGD reanalysis differs from the former analysis, with only the common WGD event in Saliaceae observed). We therefore deleted this paragraph.

Genetic structure and demographic analysis

Comment 12: -) Please add a table with population specific values of theta, Taj D , Fst calculated by excluding genic regions from the data.

Response: Ok, we have added this table in the revised manuscript (Supplementary Table 24), containing θπ, Tajima's D, *Fst* of each population with and without genic region.

Comment 13: -) authors should report the cross-validation error plot for the structure results.

Response: The cross-validation error plot is provided as follows, but fastSTRUCTURE chose optimal ancestral clusters (*K*) using the chooseK.py function rather than cross-validation error, which was 7 chosen by this function. We therefore added how fastSTRUCTURE determine the optimal *K* in Method section of the revised manuscript.

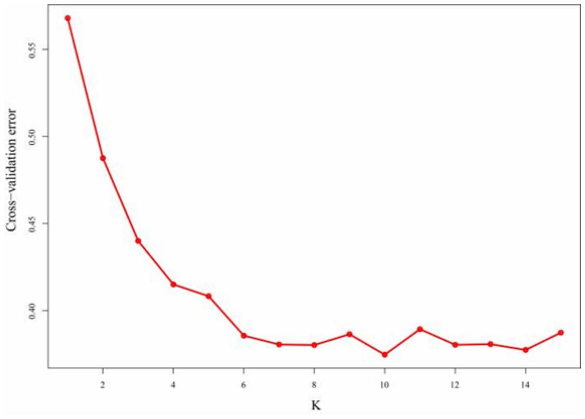

Comment 14: -) Model-based phylogenetic inference as presented in sup Figure 9 is not an acceptable representation of intraspecific variation data. This is because recombination can occur within each locus/gene and violate the assumption of a bifurcating tree assumed by the method. Based on this, I suggest to remove this analysis from the manuscript. Also it does not provide

additional information to the structure and pca results. (I feel less strongly about the NJ tree because it can be seen as a clustering method and not an explicit model-based inference method)

Response: We agree and have removed this model-based phylogenetic inference for intraspecific relationships.

Comment 15: -) tree mix does not infer a phylogenetic tree because the units here are not species but populations. Rather it infers the patterns of split and mixture in the history of a set of populations.

Response: We rephrased this as : "A population graph (Fig. 3e) inferred by Treemix27 indicated similar relationships between populations..."

Comment 16: -) line 286. I agree that the Fst can be influenced by the distance/migration between populations in this study. But it could also be affected by the history of recent population splits (see Figure 3d). See my comment below about model-based demographic inference .

Response: We have estimated split time and gene flow for six populations pairs (because this analysis is time consuming, we chose six representative pairs) using fastisimcoal2 based on neutral SFS as suggested, we found split time of these population pairs is about 0.18-0.42 Ma (which is not recent), with very weak gene flow among them (Supplementary Table 19). Structure analysis revealed admixed individuals mainly occurred between nearby populations, so we think it's safe to deduce this statement.

Comment 17: -) stairway plot analysis: this is a very interesting analysis, which seems to have been conducted very carefully (SFS estimated with ANGSD, re-estimation of the mutation rate). How is this method affected by the significant population differentiation reported in this manuscript? The authors do not specify which populations/accessions have been used to create the SFS used for the stairway analysis. I would like to see a figure with one stairway plot for each identified cluster in the structure analysis (Figure 3a).

Response: We have consulted with the author of stairway plot software. He said that an assumption of the stairway plot is random mating within the population. Thus, as we found a distinct population structure in *S. brachista*, we performed the analysis on each of the genetic groups identified by Structure analysis (Fig. 3f, Supplementary Fig. 10). The text has been revised accordingly.

Comment 18: -) Figure 3e: please add a panel showing the paleoclimatic record for the time range presented on the x-axis of figure 3e (a continuous variable indicating temperature or a proxy of temperature). This will allow a better appreciation of whether the estimated Ne values really correlate with past climatic events.

Response: OK, we have added a $\delta^{18}O$ curve, which is based on composite stable oxygen isotope ratios from benthic foraminifera and is an indicator of global ice volume and temperature

(Fig. 3f, Supplementary Fig. 10). Moreover, since bottleneck events are population or mountain specific and do not accord with the five distinct glaciation periods, we deleted these glaciation periods from figures and text.

Comment 19: Line 295: there is no figure 3f

Response: Sorry for this mistake, it should have been Fig 3e. However, we added a panel to Figure 3, so there is Figure 3f in the revised manuscript.

Comment 20: Line 302: The confidence interval around the Ne estimates shrinks to 0 for that period. Is there a good explanation for this? Can we trust the results of this method for the time interval [0;20000 generations]?

Response: In our new stairway plot analysis based on genetic groups, some have confidence intervals for *Ne* estimates shrinking to 0 for some period while others do not. We think this is a normal output from the analysis.

Comment 21: Line 307-311: this sentence sounds like the HD mountains were adapted to subnival conditions. Is this what the authors meant to write? Did they mean that the Cushion willow was well adapted to the HD mountains during glacial periods?

Response: We mean Cushion willow was well adapted to subnival conditions in HDM, so we rephrased it as: "... it was well adapted to subnival conditions...".

Origin of cushion willow associated with HDM tectonic events.

Comment 22: It is not acceptable to use phylogenetic reconstruction to evaluate the demographic history of these populations. As mentioned before this is because there is not a single bifurcating tree underlying this genomic data but many (due to recombination), see https://www.nature.com/articles/s41559-018-0717-x for an example. Instead the authors should estimate the age of the splits between the different genetic clusters (or main clusters) using either the software dadi (https://bitbucket.org/gutenkunstlab/dadi) or fastsimcoal2 (http://cmpg.unibe.ch/software/fastsimcoal2/). Note that the authors should try to construct the SFS using neutral sites (i.e excluding genic regions for example)

Response: Thank you. In the revised manuscript, we have estimated split time and gene flow for six populations pairs (because this analysis is time consuming, we chose six representative pairs) by fastisimcoal2 based on SFS constructed using neutral sites.

Selection on Cushion willow at high and low altitudes.

Comment 23: In its current form the genome scan for selection to higher altitudes does not meet the standards of the field. The authors should compare the distribution of the statistic(s) used for scanning the genome to a null distribution obtained from a neutral demographic model. Such a model can estimated using one of the two tools mentioned above (dadi or fastsimcoal2). The

authors should use these models to define a statistical threshold indicating how extreme the genome scan statistics should be expected to be given the demographic history of the cushion willow. Also note that tests like HapFLK, EHH (his, XP-EHH, implemented in selscan) and the CLR test implemented in the software sweepfinder are likely to be more powerful to detect selective sweeps than the statistics used by the authors so far.

Response: In the revised manuscript, we have not used *Fst* and θπ outliers to scan for selective sweep regions. Instead, we scan for selective sweep region using CLR implemented in sweepfinder2 based on site ancestral state as suggested. We identify selected region using the top5% CLR values, this kind of criteria, that using genome-wide distribution as a null for identifying selective sweep was also used in some recent studies (see references below). We did not found significantly enriched GO/KEGG terms for the genes residue in the sweep region, we therefore discuss some genes with the very high CLR values.

(1) Zhou X. M., et al. Population genomics of finless porpoises reveal an incipient cetacean species adapted to freshwater. Nature Communications 9, (2018).

(2) Liang Z. C., et al. Whole-genome resequencing of 472 Vitis accessions for grapevine diversity and demographic history analyses. Nature Communications 10, (2019).

(3) Zeng X., et al. Origin and evolution of qingke barley in Tibet. Nature Communications 9, 5433 (2018).

Comment 24: Please, also indicate what is known about the breeding system of the species. Is it an outcrosser or a selfer?

Response: The Saliaceae family is dioecious, so *S. brachista* is an outcrosser. We have added this information in the Introduction (line 69).

Minor

Comment 25: 149: Where are the references to these datasets?

Response: We have added the URL and reference for the genome datasets used in our study (Supplementary Table 23).

Comment 26: Figure 2a: The tracks “iii” and “iV” of the circus plot can not be read from a printed version. Even by zooming in track “iv” does not seem to display any information.

Response: Sorry, we have added these tracks (i-vi) in the circus plot (Fig. 2a) in the revised manuscript.

Reviewers' comments:

Reviewer #1 (Remarks to the Author):

Alpine plant divergence in the Hengduan Mountain biodiversity hotspot: insights from a genome-wide evolutionary analysis of Cushion willow

This manuscript is greatly improved over the original submission. The manuscript describes the genome of the cushion willow and makes inferences about adaptation to high elevation based on gene family expansion and population genomics analyses. They also infer that the sky island distribution of the populations has contributed to diversification of this and other species in the Hengduang Mountain region of China. The manuscript is well written and the results are quite interesting. The authors have also made many satisfactory responses to the original reviews. The genome assembly is much-improved and the authors have satisfactorily addressed concerns about the previous analyses of genome size and heterozygosity. The selective sweep analysis is also greatly improved, and yielded some very interesting results. I also think that the authors have sufficiently reduced their range of inference and the statements they make about diversification in the THR and HDM are now much more consistent with the data that they present in the manuscript. Nevertheless, I have detected some persistent and additional flaws in the analyses and interpretations, and these greatly dimmed my enthusiasm for this work.

1. The authors do not specify the comparative database used for the GO and KEGG enrichment analyses. What is enrichment being compared to? These methods need to be expanded, because this is absolutely critical to proper interpretation of these tests. As presented, I cannot tell what is being compared to what. To be more specific, the authors initially report on gene orthology analyses involving 10 Malpighiales species, which should give a very robust picture of which genes are exclusive or enriched in cushion willow. They then report the GO enrichment results. What is the input gene list for this analysis, and what is the background? For the questions being posed, I think the optimal analysis would focus on genes that are unique or expanded (or rapidly evolved) in cushion willow compared to the other Malpighiales, and the background set should be the full complement of genes in cushion willow. Other gene lists or reference databases could also be appropriate, but the question being posed needs to be clearly stated, and the reference database should be clearly identified. Based on the legends of the supplementary tables I guess that the input list is correct, though this needs to be explicitly stated in the manuscript. I can't find any information about the background set(s).
2. Even if the GO enrichment analyses are conducted correctly, I still do not find them to be convincing evidence of adaptation to high altitude conditions. The authors are highlighting a small subset of the categories that are significantly enriched based on their a priori biological expectations that this enrichment will reflect high altitude adaptation. There are 54 and 26 over-represented GO categories reported in Supplemental Tables 11 and 12, respectively, and only 4 of these are picked out to show evidence of adaptation to high elevations, and reasonable alternative interpretations could be provided for each of these four categories. The analysis is slightly improved over the original version by eliminating categories that are not significant after correction for multiple testing, and the comparison to the other Salix species is certainly an improvement. However, this approach would be much stronger if there were a more direct comparison of the differences in gene family size between the closely-related species and a focus on expansion of genes for which there is a reasonable expectation that the selective force could be high elevation. The GO analysis lacks specificity in this regard, and therefore is insufficient for a journal of this prominence.
3. The authors have missed the opportunity to present some potentially interesting comparative analyses. For example, they report the repeat composition of the genome, but they do not put it into context. The genome consists of ~9% Gypsy and ~9% Copia elements. I believe this number is quite low compared to other sequenced Salix and Populus, but the authors do not make these comparisons. Are the repeats different or shared with other sequenced Salix? Since there are at least 3 public Salix genomes now (S. purpurea, S. suchowensis, S. viminalis) and at least 3 public Populus genomes (P. trichocarpa, P. euphratica, and P. tremula), there is really no reason not to do this, and it would add

substantial value to the manuscript.
4. The selective sweep analysis is greatly improved over the original version, and it yielded some interesting results that lend more credence to the claim of high altitude adaptation than the GO/KEGG analyses. However, I think this analysis is flawed because it suffers from a severe Wahlund effect based on combining populations that are well differentiated into false groups based on their elevation. The neutral population structure analyses clearly show that the low elevation populations LJ and MN are strongly differentiated, yet the selective sweep analysis is performed on merged populations. This is likely to flatten the site frequency spectrum, which will affect the null distribution and have unpredictable impacts on individual loci. If this effect is differentially affecting the merged lowland versus highland populations, inferences about differential selection are likely to be flawed due to possible artifacts driven by different power in the two analyses. It would be preferable to run these analyses on undifferentiated populations with normalized sizes (i.e., the same number of individuals for the populations that are being compared).
5. The authors do not specify the criteria they used to define "rapid evolution" of gene families (lines 172-173)
6. Do the authors have permission to publish the Populus deltoides comparative analysis? As far as I know, this is still protected by the JGI data embargo. In fact, the authors do not specify where they acquired any of the comparative data that they used in this thesis. At the very least they should specify the source and version of the genomes used for the analyses.
7. The use of color for the population dots in Fig. 1e is potentially confusing because some of the same colors are also used in the FastStructure plots, where they have a different meaning. I suggest just using one color for the population dots, which is not reused for the FastStructure charts. The first two letters of the population names are sufficient to represent the groupings of the collections
8. The Supplementary Materials are not well organized within the provided files. It is not obvious from the file names where to find particular figures and tables, and figures and tables are mislabeled and mixed within Excel files. For example, the file 198041_1_related_ms_3888373_ptdwfy.xlsx contains a sheet labeled Figure 3d that is actually a table, and Supplementary Table 1 is actually an excel sheet with a bunch of figures. And Supplementary Figure 13 is actually a table. The file 198041_1_data_set_3888370_ptdw3c.xlsx is much better organized, but the supplemental figures are not labeled, so it is difficult to know if they are in the correct order. This may have been due to upload problems during review, but in any case I recommend that these be improved for the published manuscript.
9. Lines 260-261: The authors discuss long-distance dispersal by seed as an explanation for shared genome composition between distantly-related populations. I expect that long-distance pollen dispersal is more likely, but of course this depends on the pollination vector (wind vs insect, and which insects).
10. As pointed out by a previous reviewer, the assumption of a 3 year generation time is probably not justified. First, the citation provided is apparently not in the peer-reviewed literature, and it was provided by a willow breeder for the purpose of planning a breeding program. There is a big difference between flowering time in a nursery bed with abundant resources and flowering time in a harsh wild environment. Second, flowering time is not the same as generation time in the wild. In reality, seed establishment opportunities may only occur a handful of times over the lifetime of a successful plant, and for a clonal plant like cushion willow, this could mean an effective generation time of decades. A better estimate is needed, or at the very least a range should be tested. Nevertheless, since the authors are no longer trying to relate the timing of bottlenecks to geological events, this criticism is not fatal for the manuscript, since the relative comparisons among populations should mostly be valid.
11. Although it is generally well-written, some of the text, particularly the newer sections, have not undergone sufficient language editing. I have marked up the pdf of the manuscript to facilitate these revisions.

Reviewer #2 (Remarks to the Author):

My review to R1

I am very pleased with the answers to my questions, comments and suggestions regarding the original version of the manuscript.

I am attaching an annotated pdf version of the main manuscript text, with some minor, mostly editorial corrections and comments.

With regard to the response to my “comment 45” in the first revision, I would like to suggest to the authors to also include one or two short sentences in their main ms text, providing some of the information they have given in their reply (“Thanks for this valuable suggestion. S. brachista only grows in open habitats: habitats in subnival scree slope are completely open (Fig. 1d), while in low elevation localities around 3000 m, S. brachista is only found on open river banks and never occurs in forest near the river (Fig. 1c). Therefore, it seems that competition of lowland and subnival populations of Cushion willow with co-occuring plant species are unlikely to be different.”).

Alexandra Muellner-Riehl

Reviewer #3 (Remarks to the Author):

I am pleased by how the authors addressed my comments. There is only one aspect that I would recommend to improve:

In their selective sweep analysis, the authors consider as significant (i.e. putative sweeps) all regions with CLR values falling within the top 5% of the distribution.

The standard in the field, however, is too calculate the statistical threshold for the CLR statistic using neutral simulations obtained from the best demographic model (as obtained by fastsimcoal2 in this study for example).

This is actually relatively easy to do because fastsimcoal2 creates a file called "project_maxL.par" that can be used as input file for fastsimcoal2 to SIMULATE DNA data under your best demographic mdoel for the high and low populations. A bit of scripting may be required to transform the simulated data to the correct input for Sweepfinder2.

this procedure will allow the authors to identify "how high" the CLR value can be in their system in the absence of any positive selection/ adaptation

minor: what do the author mean by "based on SAS" at line 614

congratulations for this very nice work

Dear Editor and Reviewers:

We would like to thank you for your positive and constructive comments and suggestions which have been very helpful in revising and improving our manuscript. We have carefully revised our manuscript and provide below point-by-point responses (in blue) to all comments received. The main change are that: (1) in order to improve presentation, we have changed sections around to improve the sequence of analyses presented and overall flow; (2) We have modified the enrichment analysis section, and discuss only top enriched terms. We further compare gene numbers for gene families involved in these terms between the *Salix* species examined; (3) We added a comparative analysis for LTR/Copia and LTR/Gyspy retrotransposon repeat families as suggested by reviewer #1; (4) We used three different (3, 10 and 15 years) generation times for demographic analysis as suggested by reviewer #1, which yield similar results; (5) We redid the selective sweep analysis based on undifferentiated populations as addressed by reviewer #1, and we used neutral simulations to determine CLR value thresholds for identifying sweep regions as suggested by reviewer #3.

Revised portions of the manuscript are marked in red (which are according to revisions of the annotated PDF file provided by reviewer #1 and #2) or blue (which are revisions to address each reviewer's concerns, or those based on new analysis).

Reviewers' comments:

Reviewer #1 (Remarks to the Author):

Alpine plant divergence in the Hengduan Mountain biodiversity hotspot: insights from a genome-wide evolutionary analysis of Cushion willow

This manuscript is greatly improved over the original submission. The manuscript describes the genome of the cushion willow and makes inferences about adaptation to high elevation based on gene family expansion and population genomics analyses. They also infer that the sky island distribution of the populations has contributed to diversification of this and other species in the Hengduang Mountain region of China. The manuscript is well written and the results are quite interesting. The authors have also made many satisfactory responses to the original reviews. The genome assembly is much-improved and the authors have satisfactorily addressed concerns about the previous analyses of genome size and heterozygosity. The selective sweep analysis is also greatly improved, and yielded some very interesting results. I also think that the authors have sufficiently reduced their range of inference and the statements they make about diversification in the THR and HDM are now much more consistent with the data that they present in the manuscript. Nevertheless, I have detected some persistent and additional flaws in the analyses and interpretations, and these greatly dimmed my enthusiasm for this work.

Response: Thanks for positive comments for our study. We accepted all the revisions in the

attached annotated PDF file, which are marked as red in the revised manuscript. And we made revisions according to concerns addressed, please see responses below.

1. The authors do not specify the comparative database used for the GO and KEGG enrichment analyses. What is enrichment being compared to? These methods need to be expanded, because this is absolutely critical to proper interpretation of these tests. As presented, I cannot tell what is being compared to what. To be more specific, the authors initially report on gene orthology analyses involving 10 Malpighiales species, which should give a very robust picture of which genes are exclusive or enriched in cushion willow. They then report the GO enrichment results. What is the input gene list for this analysis, and what is the background? For the questions being posed, I think the optimal analysis would focus on genes that are unique or expanded (or rapidly evolved) in cushion willow compared to the other Malpighiales, and the background set should be the full complement of genes in cushion willow. Other gene lists or reference databases could also be appropriate, but the question being posed needs to be clearly stated, and the reference database should be clearly identified. Based on the legends of the supplementary tables I guess that the input list is correct, though this needs to be explicitly stated in the manuscript. I can't find any information about the background set(s).

Response: We agree with Rev 1 that we did not describe the GO and KEGG enrichment analysis in sufficient detail in the previous version. We tested GO terms and KEGG pathway enrichments using expanded, rapidly-evolved or species-specific sets of genes for the species examined (*Salix brachista*, *Salix purpurea* and *Salix suchowensis*) as input gene lists, and using all the genes of the genome with GO or KEGG annotations of the examined species (*Salix brachista*, *Salix purpurea* and *Salix suchowensis*) as background. We explain our approach in the revised manuscript (Lines 232-235 and Lines 586-588).

2. Even if the GO enrichment analyses are conducted correctly, I still do not find them to be convincing evidence of adaptation to high altitude conditions. The authors are highlighting a small subset of the categories that are significantly enriched based on their a priori biological expectations that this enrichment will reflect high altitude adaptation. There are 54 and 26 over-represented GO categories reported in Supplemental Tables 11 and 12, respectively, and only 4 of these are picked out to show evidence of adaptation to high elevations, and reasonable alternative interpretations could be provided for each of these four categories. The analysis is slightly improved over the original version by eliminating categories that are not significant after correction for multiple testing, and the comparison to the other Salix species is certainly an improvement. However, this approach would be much stronger if there were a more direct comparison of the differences in gene family size between the closely-related species and a focus on expansion of genes for which there is a reasonable expectation that the selective force could be high elevation. The GO analysis lacks specificity in this regard, and therefore is insufficient for a journal of this prominence.

Response: Our expectation is that since *Salix brachista* is an alpine plant, we should find some hints for adaptation to alpine conditions, such as response to UV radiation (that can cause DNA damage), and low oxygen level. Some of the enriched terms may seem cherry-picked, and we agree there may be reasonable alternative interpretations for enrichment of these categories. So, we now only discuss the top two enriched KEGG terms in expanded gene families (base excision repair, flavonoid biosynthesis), and the GO terms related to flavonoid/anthocyanin biosynthesis. We further compare the gene numbers of the gene families involved in these terms between the three *Salix* species examined. We found that *Salix brachista* has more genes for most of the gene families than the other two non-alpine *Sali*x species (supplementary table 15). These modifications to the manuscript are presented in "Genomic footprints of alpine adaptationlines" start from Line 232 in the revised manuscript.

We think these results provide indications of genomic footprints of alpine adaptation in *Salix brachista*. Of course further in-depth studies are required to prove this, but our results could provide indications and candidate genes for such future research.

3. The authors have missed the opportunity to present some potentially interesting comparative analyses. For example, they report the repeat composition of the genome, but they do not put it into context. The genome consists of ~9% Gypsy and ~9% Copia elements. I believe this number is quite low compared to other sequenced Salix and Populus, but the authors do not make these comparisons. Are the repeats different or shared with other sequenced Salix? Since there are at least 3 public Salix genomes now (S. purpurea, S. suchowensis, S. viminalis) and at least 3 public Populus genomes (P. trichocarpa, P. euphratica, and P. tremula), there is really no reason not to do this, and it would add substantial value to the manuscript.

Response: Thanks for this suggestion. We did the LTR/Copia and LTR/Gypsy analysis for six Saliaceae species (we did not use *S. viminalis* because the genome assembly is too fragmented and still not publically available). Our results show that in comparison to genomes of closely related other Salicaceae species, Copia elements appear to have expanded in Cushion willow. In contrast, Gypsy elements have contracted in Salix species examined, although Cushion willow has the highest proportion among the Salix species. A phylogenetic analysis of Copia and Gypsy families across the six Salicaceae species revealed no evidence for any species-specific family members, demonstrating that Copia/Gypsy divergence predates the divergence of these species. (See Lines 132-141).

4. The selective sweep analysis is greatly improved over the original version, and it yielded some interesting results that lend more credence to the claim of high altitude adaptation than the GO/KEGG analyses. However, I think this analysis is flawed because it suffers from a severe Wahlund effect based on combining populations that are well differentiated into false groups based on their elevation. The neutral population structure analyses clearly show that the low elevation populations LJ and MN are strongly differentiated, yet the selective sweep analysis is

performed on merged populations. This is likely to flatten the site frequency spectrum, which will affect the null distribution and have unpredictable impacts on individual loci. If this effect is differentially affecting the merged lowland versus highland populations, inferences about differential selection are likely to be flawed due to possible artifacts driven by different power in the two analyses. It would be preferable to run these analyses on undifferentiated populations with normalized sizes (i.e., the same number of individuals for the populations that are being compared).

Response: We agree with these comments. So we use undifferentiated high (LJ_XF and LJ_LS) and low (LJ_BS and LJ_GH) elevation LJ populations, which are located on the same Yulong mountain in Lijiang county. Structure analysis and PCA analysis indicate these high and low populations are undifferentiated and therefore appropriate for selective sweep analysis. Nine individuals from each of the high and low elevation groups were included in the analysis. Furthermore, as suggested by reviewer #3, we used neutral simulation to determine the threshold of CLR scores to identify sweep regions. We revised the corresponding figures and tables (Fig 4c, 4d, Supplementary Fig. 14, Supplementary Table 22) based on results of the new analysis.

5. The authors do not specify the criteria they used to define "rapid evolution" of gene families (lines 172-173)

Response: We use the default parameters for rapidly evolved gene family identification. The criteria for this is as the manual of CAFE software describes:

"The branch-specific *p*-values are obtained by the Viterbi method with the randomly generated likelihood distribution. This method calculates exact *p*-values for transitions between the parent and child family sizes for all branches of the phylogenetic tree. A low *p*-value indicates a rapidly evolving branch. This information is reported only for the families with an overall *p*-value less than the *p*-value cutoff set with the load command."

Since we use the default parameter of CAFE, we chose not to report it in detail in the Method section, and we just state that we used the default parameters.

6. Do the authors have permission to publish the Populus deltoides comparative analysis? As far as I know, this is still protected by the JGI data embargo. In fact, the authors do not specify where they acquired any of the comparative data that they used in this thesis. At the very least they should specify the source and version of the genomes used for the analyses.

Response: We have contacted (by email) the principal investigator of the genome project of *Populus deltoides*, professor Gerald A. Tuskan. He told us that there would be no restrictions in the use of this data in a comparative gene family analysis focused on *Salix* and cite JGI and Phytozome as our source of data. If necessary, we can provide these emails.

7. The use of color for the population dots in Fig. 1e is potentially confusing because some of the same colors are also used in the FastStructure plots, where they have a different meaning. I

suggest just using one color for the population dots, which is not reused for the FastStructure charts. The first two letters of the population names are sufficient to represent the groupings of the collections.

Response: Sorry for the confusion, we have changed all the population dots to black in the new version of Fig. 1e.

8. The Supplementary Materials are not well organized within the provided files. It is not obvious from the file names where to find particular figures and tables, and figures and tables are mislabeled and mixed within Excel files. For example, the file 198041_1_related_ms_3888373_ptdwfy.xlsx contains a sheet labeled Figure 3d that is actually a table, and Supplementary Table 1 is actually an excel sheet with a bunch of figures. And Supplementary Figure 13 is actually a table. The file 198041_1_data_set_3888370_ptdw3c.xlsx is much better organized, but the supplemental figures are not labeled, so it is difficult to know if they are in the correct order. This may have been due to upload problems during review, but in any case I recommend that these be improved for the published manuscript.

Response: We are sorry for the organization of the Supplementary Material. The name of these files seem to be automatically renamed by the submission system. We will carefully check this problem when we upload the revised manuscript files.

9. Lines 260-261: The authors discuss long-distance dispersal by seed as an explanation for shared genome composition between distantly-related populations. I expect that long-distance pollen dispersal is more likely, but of course this depends on the pollination vector (wind vs insect, and which insects).

Response: Willow species are mainly insect-pollinated, but some willows are also pollinated by wind. In a recent study by Perdereau et al. (High levels of gene flow and genetic diversity in Irish populations of *Salix caprea* L. inferred from chloroplast and nuclear SSR markers. BMC Plant Biology, 2014, 14:1-12.), it was observed that gene flow via willow seeds and pollen can extend over more than 200 km. We therefore added in Lines 288-289: “long-distance dispersal is feasible via pollen”.

10. As pointed out by a previous reviewer, the assumption of a 3 year generation time is probably not justified. First, the citation provided is apparently not in the peer-reviewed literature, and it was provided by a willow breeder for the purpose of planning a breeding program. There is a big difference between flowering time in a nursery bed with abundant resources and flowering time in a harsh wild environment. Second, flowering time is not the same as generation time in the wild. In reality, seed establishment opportunities may only occur a handful of times over the lifetime of a successful plant, and for a clonal plant like cushion willow, this could mean an effective generation time of decades. A better estimate is needed, or at the very least a range should be tested. Nevertheless, since the authors are no longer trying to relate the timing of

bottlenecks to geological events, this criticism is not fatal for the manuscript, since the relative comparisons among populations should mostly be valid.

Response: In the revised ms we now use a range of generation times in analyses, i.e. 3, 10 and 15 years. Three years, because according to the literature we listed, willows are thought to have short generation time, for example, *Salix viminalis* has a generation of 1-2 years according to Hallingbäck et al (Genome Wide Associations of Growth, Phenology, and Plasticity Traits in Willow [*Salix viminalis* (L.)]. Frontiers in Plant Science, 2019, 10: 753). Ten years was used by Ikeda et al (Post-glacial East Asian origin of the alpine shrub *Phyllodoce aleutica* (Ericaceae) in Beringia. Journal of Biogeography, 2018, 45: 1261-1274) in *Phyllodoce aleutica*, which is also a shrub distribute in harsh environment of alpine and arctic. Fifteen years, was used for *Populus tremula* and *Populus tremuloides* by Wang et al (Variation in Linked Selection and Recombination Drive Genomic Divergence during Allopatric Speciation of European and American Aspens. Molecular Biology and Evolution, 2016, 33:1754-1767).

Similar population demographic histories are produced for each generation time, although the timing of bottlenecks etc. does of course vary. Most bottlenecks occurred within the last 3 millions years, during which there has been intensive climate fluctuation. A few of the bottlenecks date to the late Miocene and Pliocene with a generation time of 15 years, which is not unexpected given that climate fluctuations started from the middle Miocene (15-7 Ma, weak climate fluctuations; 7-3 Ma, more pronounced climate fluctuations; 3 Ma intensive climate fluctuations, according to review of Muellner-Riehl: Mountains as Evolutionary Arenas, Frontiers in Plant Science, 2019, 10: 195).

We think the use of 15 years as the maximum generation time is reasonable, because by using this generation time, time of demographic events of the DC population were estimated to start from around 16.4 Ma (Supplementary Fig 13c), which is close to the upper bound of the estimated divergence time of *Salix brachist*a with one of its close relative *Salix souliei*, i.e., 11.73 Ma (95% 95% confidence intervals: 6.83-18.34 Ma) (Supplementary Fig 9).

Our main result is that demographical history was affected by climate fluctuation, so although we used different generations times, our conclusions still stands.

11. Although it is generally well-written, some of the text, particularly the newer sections, have not undergone sufficient language editing. I have marked up the pdf of the manuscript to facilitate these revisions.

Response: Sorry for this, we have checked thoroughly for language improvement in the revised manuscript.

Reviewer #2 (Remarks to the Author):

My review to R1

I am very pleased with the answers to my questions, comments and suggestions regarding the original version of the manuscript.

I am attaching an annotated pdf version of the main manuscript text, with some minor, mostly editorial corrections and comments.

With regard to the response to my “comment 45” in the first revision, I would like to suggest to the authors to also include one or two short sentences in their main ms text, providing some of the information they have given in their reply (“Thanks for this valuable suggestion. S. brachista only grows in open habitats: habitats in subnival scree slope are completely open (Fig. 1d), while in low elevation localities around 3000 m, S. brachista is only found on open river banks and never occurs in forest near the river (Fig. 1c). Therefore, it seems that competition of lowland and subnival populations of Cushion willow with co-occuring plant species are unlikely to be different.”).

Alexandra Muellner-Riehl

Response: Thanks very much for positive comments for our study. We added sentences to describe the competition of high and low elevation Cushion willow plant in Lines 367-368. We also accepted all the revisions in the attached annotated PDF file, which are marked as red in the revised manuscript.

Reviewer #3 (Remarks to the Author):

I am pleased by how the authors addressed my comments. There is only one aspect that I would recommend to improve:

In their selective sweep analysis, the authors consider as significant (i.e. putative sweeps) all regions with CLR values falling within the top 5% of the distribution.

The standard in the field, however, is too calculate the statistical threshold for the CLR statistic using neutral simulations obtained from the best demographic model (as obtained by fastsimcoal2 in this study for example).

This is actually relatively easy to do because fastsimcoal2 creates a file called "project_maxL.par" that can be used as input file for fastsimcoal2 to SIMULATE DNA data under your best demographic mdoel for the high and low populations. A bit of scripting may be required to transform the simulated data to the correct input for Sweepfinder2. this procedure will allow the authors to identify "how high" the CLR value can be in their system in the absence of any positive selection/ adaptation

minor: what do the author mean by "based on SAS" at line 614

congratulations for this very nice work

Response: Thanks very much for positive comments for our study. As advised, we used

neutral simulation to determine CLR thresholds of sweep region, please see the Method section (Lines 693-701) in the revised manuscript. Moreover, we did the selective analysis as suggested by reviewer #1, that we used undifferentiated populations of high and low elevation LJ populations, which located in the same mountain.

“based on SAS” here means SFS (site frequency spectrum) are estimated by SAS (site ancestral state), all abbreviations including these two used in this manuscript were defined at first use.

REVIEWERS' COMMENTS:

Reviewer #1 (Remarks to the Author):

The authors have done an excellent job of responding to my previous criticisms, and I have no further substantive suggestions about this manuscript. I noticed three very minor issues when reading the latest draft.

line 366: although can also-> although it can also
line 377: ~4.24.6 Mb -> ~4.2-4.6

Availability of data: The GenBank accession number appears to be a placeholder. The authors should be sure to update this with an actual accession number in the final manuscript.

Reviewer #3 (Remarks to the Author):

I am satisfied with the authors response to my comments. congratulations

Point-by-point response to reviewer's comments for rev#2

REVIEWERS' COMMENTS:

Reviewer #1 (Remarks to the Author):

The authors have done an excellent job of responding to my previous criticisms, and I have no further substantive suggestions about this manuscript. I noticed three very minor issues when reading the latest draft.

Response: Thanks very much for positive comments for our study and we greatly appreciate reviewers' time and expertise.

line 366: although can also-> although it can also

line 377: ~4.24.6 Mb -> ~4.2-4.6

Response: We have corrected these issues in the revised manuscript.

Availability of data: The GenBank accession number appears to be a placeholder. The authors should be sure to update this with an actual accession number in the final manuscript.

Response: These are actual NCBI GenBank accession numbers which are not publicly released yet. Now data relate with all of these NCBI accession numbers are released and publicly available.

Reviewer #3 (Remarks to the Author):

I am satisfied with the authors response to my comments. congratulations

Response: Thanks very much for positive comments for our study and we greatly appreciate reviewers' time and expertise.
